# A neuropeptide, Substance-P, directly induces tissue-repairing M2 like macrophages by activating the PI3K/Akt/mTOR pathway even in the presence of IFNγ

**DOI:** 10.1038/s41598-017-09639-7

**Published:** 2017-08-25

**Authors:** Ji Eun Lim, Eunkyung Chung, Youngsook Son

**Affiliations:** 1 Department of Genetic Engineering, College of Life Science and Graduate School of Biotechnology, Kyung Hee University, Yong In, 17104 Republic of Korea; 2 Kyung Hee Institute of Regenerative Medicine, Kyung Hee University Hospital, Seoul, Republic of Korea; 3 BIO R&D center, L&K BIOMED CO. LTD., Seoul, Republic of Korea

## Abstract

Macrophage polarization plays an important role in tissue damage and repair. In this study, we show that Substance-P (SP) can directly induce M2 polarization of inflammatory macrophages. SP induced the differentiation of GM-CSF-differentiated pro-inflammatory macrophages into alternatively activated phagocytic M2 like macrophages (M2^SP^) through direct activation of the PI3K/Akt/mTOR/S6kinase pathway and induction of Arginase-1, CD163, and CD206, all of which were nullified by pretreatment with the neurokinin-1 receptor (NK-1R) antagonist RP67580 and specific signaling pathway inhibitors. M2^SP^ were distinct from IL-4/IL-13-induced M2a and IL-10-induced M2c subtypes; they did not show STAT activation and exhibited high phagocytic and endothelial adhesive activity. Furthermore, SP had a dominant effect on M2 polarization over Interferon gamma (IFNγ), a potent M1-skewing cytokine, and effectively induced the M2 phenotype in monocytes and the human THP-1 cell line. Finally, adoptively transferred M2^SP^ migrated to a spinal cord injury (SCI) lesion site and improved functional recovery. Collectively, our findings show that SP, a neuropeptide, plays a role as a novel cytokine by inducing tissue-repairing M2^SP^ macrophages and thus may be developed for pharmacological intervention in diseases involving chronic inflammation and acute injury.

## Introduction

Macrophages are essential components of the innate and adaptive immune systems and play central roles in inflammation and host defense^1, 2^. These cells are functionally classified into two major types: classically activated, proinflammatory (M1) macrophages and alternatively activated (M2) macrophages^3, 4^. M1 macrophages are induced by Th1 cytokines such as IFNγ and Granulocyte macrophage colony stimulating factor (GM-CSF) or lipopolysaccharide (LPS) and are characterized by cytotoxic activity against bacterial and viral infections and high expression levels of proinflammatory cytokines and chemokines. By contrast, M2 macrophages are induced by Th2 cytokines, such as IL-4, IL-13, IL-10, and TGF-βs, and they are characterized by efficient phagocytosis of dead cells and strong scavenger receptor expression with resolution of inflammation, tissue remodeling, fibrosis and tumor progression. In the acute inflammation phase during the early stage of tissue injury, neutrophils and monocytes heavily infiltrate the injured tissue from the blood to reach high M1/M2 ratios, and this is followed by the resolution of inflammation and the remodeling phase with an M2 macrophage-enriched environment. Several reports have indicated that the pathology of chronic inflammatory diseases, such as type 2 diabetes and atherosclerosis, and impaired healing is closely associated with the M1 and M2 macrophage balance^5–7^. Especially in tissue repair, M2 macrophages may terminate tissue-destructive proinflammatory responses but create a reparative environment by cleaning up apoptotic dead cells and stimulating angiogenesis and cell proliferation. This event also seems to be an important step toward the acquisition of tolerance to self-antigens of apoptotic cells and avoidance of the induction of an autoimmune response, especially in IL-10-induced deactivating M2c-type macrophages^8–11^. However, the origin and classification of these late-arriving, tissue-repairing M2 macrophages from the plastic transition of M1 macrophages^12^, infiltration of newly generated M2-skewed monocytes or local proliferation of tissue macrophages in response to the Th2 cytokine IL-4, independently of monocytes^13, 14^, remain controversial. Although molecular signatures for M1/M2 macrophages have not yet been clearly resolved in human, mouse, and rat systems, the manipulation of M2 polarization could be a tempting pharmacological target for the treatment of chronic inflammation-associated metabolic disease and tissue repair.

Multiple intracellular signaling pathways, such as the JAK/STAT, PKC/ERK, and PI3K/Akt/mTOR pathways, function in parallel or convergently in M2 polarization of macrophages or monocytes under a variety of pathophysiological conditions. The Th2 cytokines IL-4 and IL-13 (IL-4/13) induce M2 polarization by activating STAT6, and these macrophages are defined as M2a subset. The anti-inflammatory cytokine IL-10 induces the activation of STAT3 and leads to the M2c subtype^6, 12, 15–17^. Alternatively, activation of the PI3K/Akt/mTOR signaling pathway also leads to M2 polarization in steady-state macrophages or monocytes by skewing M1 macrophages to M2-type macrophages^18–20^, and PI3K/Akt/mTOR inhibitors can prevent this M2 polarization of human macrophages and redirect their differentiation toward an M1 state^21^. Bone morphogenic protein-7 (BMP-7) mediates monocyte polarization into M2 macrophages by activating SMAD/PI3K/Akt/mTOR^22^. Recently, glucose metabolism and protein metabolism have been shown to regulate macrophage polarization^19, 23^, and the involvement of AMP-activated protein kinase (AMPK) α1 in M2 polarization has been noted in a muscle regeneration model^24^. In addition, lipid metabolism is also involved in M2 polarization, as evidenced by the important mediator effects of PPAR family members in IL-4-induced M2 polarization^25, 26^. Additionally, the intracellular arginine balance seems to be an important regulator of M1/M2 polarization; nitric oxide (NO) is produced from arginine by inducible NO synthase (iNOS) in M1 macrophages, or ornithine is produced from arginine by Arginase-1 as a substrate for polyamines in M2 macrophages^27–29^. Therefore, many intracellular signaling pathways and cellular metabolic states act together during M2 polarization.

SP, an undecapeptide, is a member of the tachykinin peptide family and acts as a sensory neurotransmitter and neuromodulator related to the nociceptive pain pathway in the central nervous system. SP has generally been known to activate immune cells into proinflammatory ones^30, 31^. However, in our previous study, SP treatment enhanced recovery from spinal cord injury in rats^32, 33^. As supporting evidence, a decrease in pro-inflammatory M1 markers such as iNOS and CD86, but an increase in the anti-inflammatory M2 markers Arginase-1 and CD206, was detected at an early stage in the lesion site of SP-treated SCI rats, which was also accompanied by reduced apoptosis of neurons and oligodendrocytes and stimulation of axon outgrowth. It has been proposed that SP may modulate injury-provoked acute inflammation at an early stage of SCI and thereby increase the proportions of CD11b^+^IL-10^+^CD206^+^ M2-like macrophages in the SCI microenvironment^32, 33^. Accordingly, we hypothesized that SP may play a role as a novel M2 cytokine to modulate bone marrow-derived monocytes or inflamed tissue macrophages to polarize M2 subtype macrophages, which may contribute positively to tissue repair and healing processes by resolving inflammation, cleaning up dead cells, and creating a more receptive microenvironment for tissue repair.

To test this hypothesis, we explored whether SP can directly induce M2 type macrophages from bone marrow-derived monocytes, GM-CSF-differentiated macrophages similar to inflammatory macrophages, and the THP-1 human monocytic cell line based on the expression of M1 and M2 subtype-specific markers such as Arginase-1, iNOS, CD163 scavenger receptor, CD206 mannose receptor, CCR7, CD68, and STAT phosphorylation. For subtype classification of SP-induced macrophages, IL-4/13 for M2a, IL-10 for M2c, and IFNγ for M1 polarization were used as representative standards. To simulate the role of SP in the injured tissue microenvironment, the synergistic or antagonistic effects of combinations of cytokines, as well as the phagocytic and extravasation capacity, were analyzed. Finally, adoptive transfer of macrophages was performed in the rat SCI model to evaluate macrophage tissue infiltration capacity and tissue repair functions.

## Results

### SP regulates macrophage plasticity toward the M2 phenotype through early induction of Arginase-1 activity instead of NO production

**Figure 1:**
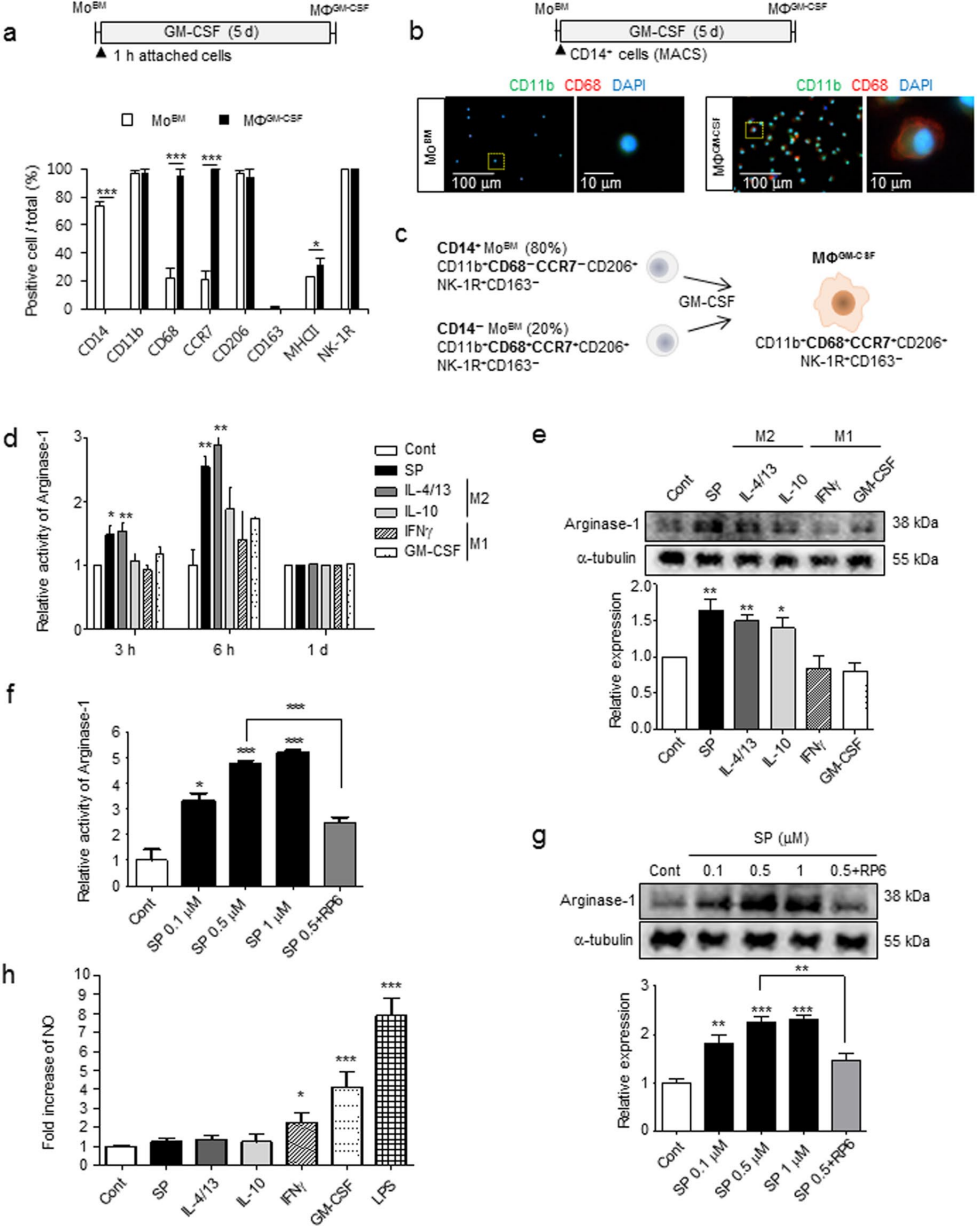
SP induces M2 polarization through early induction of Arginase-1. (**a**) Mo^BM^ were collected after 1-h of attachment and differentiated into MΦ^GM-CSF^ by GM-CSF treatment for 5 d. MΦ^GM-CSF^ (CD14^−^CD11b^+^CD68^+^CCR7^+^CD206^+^) were defined as M1-like precursors that were differentiated from Mo^BM^ (CD14^+^CD11b^+^CD68^−^CCR7^−^CD206^+^). For the immunofluorescence staining results (Supplementary Fig. 1a), the number of positive-cells was counted (5 random fields/coverslip) at 100 × magnification (0.85 mm^2^) (n = 3). (**b**) CD14^+^ MACS-isolated monocytes were differentiated with GM-CSF treatment for 5 d, and cells were further characterized as CD11b^+^CD68^+^ macrophages. (Green = CD11b, Red = CD68, Blue = DAPI) (n = 3). (**c**) Proposed model for MΦ^GM-CSF^ from Mo^BM^ with 80% CD14^+^ and 20% CD14^−^ phenotypes. (**d**) Increased Arginase-1 activity was detected at the indicated time point of SP treatment using the Arginase-1 activity assay, and the results were compared with typical M1/M2-inducing cytokines (n = 3). (**e**) The increased expression of Arginase-1 was detected after 6 h of SP treatment. The western blot samples derived from the same experiment and were quantified with ImageJ. Full-length blots are presented in Supplementary Figure 16a (n = 4). (**f**–**g**) Dose-dependent increased activity and expression of Arginase-1 determined after 6 h of SP treatment. This was completely blocked by pretreatment with the NK-1R antagonist RP67580 (1 μM). (**f**) Arginase-1 activity assay (n = 3). (**g**) The western blot samples derived from the same experiment and were quantified with ImageJ. Full-length blots are presented in Supplementary Figure 16b (n = 4). (**h**) Increased NO was detected in culture with IFNγ, GM-CSF, or LPS but not with SP, IL-4/13, or IL-10. Quantitative analysis of NO production was performed in conditioned medium from MΦ^GM-CSF^ that had been treated with SP or cytokines for 1 d. LPS treatment served as a positive control (n = 6). Data represent the mean ± SEM (*p < 0.05, **p < 0.01, ***p < 0.001, unpaired t test). Scale bar = 100 μm (**b**) and 10 μm (high-magnification image of inset in **b**).

To explore the role of SP in macrophage plasticity under the tissue injury microenvironment, monocytes derived from bone marrow mononuclear cells (Mo^BM^) were differentiated into macrophages by GM-CSF treatment for 5 d to mimic inflamed tissue macrophages since GM-CSF treatment is reported to transforms bone marrow-derived macrophages to a proinflammatory M1 type^34^. Adherent Mo^BM^ cells expressed monocyte markers such as CD14 (~80%) and CD11b (~95%), activated macrophage markers such as CD68 (~20%), CCR7 (~20%), and MHC class II (MHC II, ~20%) in a subset of adherent CD11b^+^ cells, and weak levels of a typical M2 macrophage marker, CD206 mannose receptor (Fig. 1a, Supplementary Fig. 1a). Following GM-CSF-induced differentiation, GM-CSF differentiated macrophages (MΦ^GM-CSF^) completely lost CD14 expression, while all of these macrophages showed enhanced expression of activated macrophage markers such as CD68 and CCR7 as well as CD206. However, CD163, a scavenger receptor and known typical M2 marker in rat macrophages^35–37^, was not expressed in either Mo^BM^ or MΦ^GM-CSF^. All the CD14^+^ cells, even after CD14-positive selection, were differentiated into CD14^−^CD11b^+^CD68^+^ activated macrophage after 5 d of treatment with GM-CSF (Fig. 1b). The SP receptor, NK-1R, was equally expressed in both Mo^BM^ and MΦ^GM-CSF^. The cellular morphology was visualized by actin staining. Mo^BM^ had a small round shape, and MΦ^GM-CSF^ were enlarged with an unequal distribution. Furthermore, the lack of lymphocytes, bone marrow stromal cells, osteoclasts (OC), osteoblasts (OB) in MΦ^GM-CSF^ was further confirmed by the absence of CD45, CD29, tartrate-resistant acid phosphatase (TRAP), and alkaline phosphatase (ALP) staining, respectively (Supplementary Fig. 1b,c) Accordingly, MΦ^GM-CSF^ can be defined as CD11b^+^CD68^+^CCR7^+^CD206^+^NK-1R^+^CD163^−^ pro-inflammatory activated M1-like precursors rather than anti-inflammatory M2 macrophages, despite the persistent expression of CD206 in MΦ^GM-CSF^ ^34, 38–41^ (Fig. 1c).

Next, the role of SP in the control of the macrophage phenotype was examined based on cellular usage of arginine. Pro-inflammatory M1 macrophages produce NO from arginine through the action of iNOS, but alternatively activated or tissue-repairing M2 macrophages produced ornithine and urea from arginine through the action of Arginase-1^42–44^. Accordingly, Arginase-1 activity and its protein levels were analyzed in SP-treated MΦ^GM-CSF^ and compared with IL-4/13 as a M2a cytokine, IL-10 as an M2c cytokine, and IFNγ and GM-CSF as M1 cytokines (Fig. 1d,e). Both SP and IL-4/13 elevated Arginase-1 activity in MΦ^GM-CSF^ starting at 3 h, which increased approximately 3-fold at 6 h and decreased to basal levels at 1 d (Fig. 1d). Based on western blot analysis, Arginase-1 protein was clearly elevated at 6 h in SP, IL-4/13, and IL-10-treated MΦ^GM-CSF^ (Fig. 1e) but not in IFNγ or GM-CSF-treated MΦ^GM-CSF^. The SP-stimulated elevation of Arginase-1 activity and protein levels occurred in a dose-dependent manner, and this effect was clearly blocked by pretreatment with an NK-1R antagonist, RP67580 (Fig. 1f,g). In contrast, SP did not NO production similarly to other M2 cytokines, IL-4/13 and IL-10, but IFNγ, GM-CSF, and LPS markedly stimulated NO production (Fig. 1h). Thus, SP can regulate macrophage plasticity toward an M2-specified phenotype through early induction of Arginase-1.

### Identification of SP-induced macrophages as a distinct M2 subtype compared with those stimulated with IL-4/13 and IL-10

**Figure 2:**
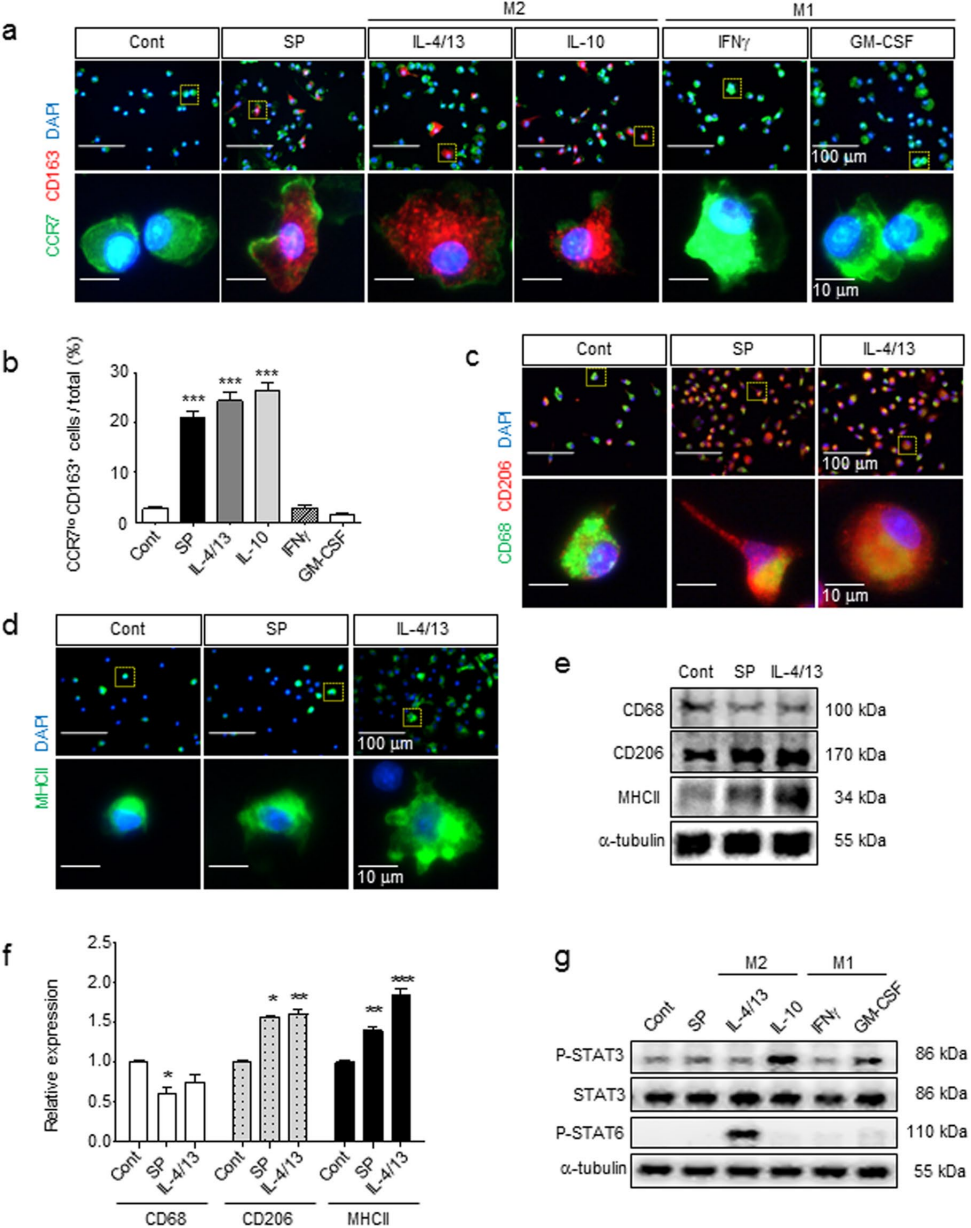
Identification of SP-induced macrophages as a distinct M2 subtype compared with IL-4/13- and IL-10-induced macrophages. (**a**,**b**) After 3 d of culture with SP or cytokines, MΦ were immunostained with specific M1/M2 markers. The induction of CD163 expression and reduction of CCR7 expression were induced to a similar extent in SP, IL-4/13 and IL-10 but not in IFNγ or GM-CSF treated with MΦ^GM-CSF^ at 3 d (Green = CCR7, Red = CD163, Blue = DAPI). The number of CCR7^lo^CD163^+^ M2 cells was counted (5 random fields/coverslip) at 100 × magnification (0.85 mm^2^) (n = 3). (**c**) Decreased expression of CD68 and increased expression of CD206 were detected in response to SP treatment, which was similar to IL-4/13 treatment (Green = CD68, Red = CD206, Blue = DAPI) (n = 3). (**d**) MHCII was up-regulated by SP treatment, but the increased expression was much higher in response to IL-4/13 treatment (Green = MHCII, Blue = DAPI) (n = 3). (**e**–**f**) After 1 d of treatment, CD68, CD206, and MHCII were examined by western blot analysis and quantified using ImageJ. The samples derived from the same experiment and full-length blots are presented in Supplementary Figure 17 (n = 3). (**g**) Neither STAT3 nor STAT6 was activated by SP treatment at 6 h, which are known to involved in the IL-10 and IL-4/13-induced signaling pathway, respectively. The samples derived from the same experiment and full-length blots are presented in Supplementary Figure 18 (n = 2). Data represent the mean ± SEM (*p < 0.05, **p < 0.01, ***p < 0.001, unpaired t test). Scale bar = 100 μm (**a**,**c**,**d**) and 10 μm (high-magnification image inset in **a**,**c**,**d**).

M2 macrophages are also classified into M2a, M2b, and M2c types, which are induced by IL-4/13, LPS, and IL-10, respectively. M2b is known to produce NO and thus more closely resembles M1-like macrophages. To identify the M2 subtype of SP-induced macrophages among the Arginase-1-expressing M2 macrophages, the M2 marker CD163 and the M1 marker CCR7 were examined (Fig. 2a,b). IL-4/13 and IL-10 clearly increased CD163-expressing cells among MΦ^GM-CSF^ (more than 25% of the total cells), but IFNγ and GM-CSF did not induce any CD163 expression (less than 3% of the total cells). Surprisingly, SP treatment significantly up-regulated CD163 expression in MΦ^GM-CSF^ to a similar degree to those of other M2-inducing Th2 cytokines. Furthermore, there was a marked reduction of CCR7, an M1 marker, accompanied by the induction of CD163. However, IFNγ-treated or GM-CSF-treated MΦ^GM-CSF^ maintained high expression levels of CCR7, which are expressed in most activated pro-inflammatory macrophages. Another M2-associated marker, the mannose receptor CD206, was expressed at low basal levels in monocytes. Both SP and IL-4/13 strongly stimulated CD206 expression. Only IL-4/13 strongly stimulated MHCII expression, supporting its role in the enhancement of antigen-presenting capacity. However, SP and IL-4/13 decreased CD68 expression (Fig. 2c–f). According to the marker expression profile, SP can be classified to Th2 cytokines such as IL-4/13 and IL-10.

To further define the SP-induced macrophages from the M2a and M2c subtypes, STAT activation was examined (Fig. 2g). STAT6 activation is involved in IL-4/13-induced M2a polarization, and STAT3 activation is required for IL-10-induced M2c polarization^6, 12, 15–17^. IL-4/13 and IL-10 clearly induced STAT6 and STAT3 activation, respectively. In contrast, neither STAT6 nor STAT3 activation was observed in SP-induced macrophages, suggesting that the SP-stimulated signaling pathway leading to M2 polarization is distinct from the IL-4/13 or IL-10 mediated pathways.

### SP activates the PI3K/Akt/mTOR signaling pathway through NK-1R, ultimately leading to Arginase-1 induction and CD163 expression in macrophages

**Figure 3:**
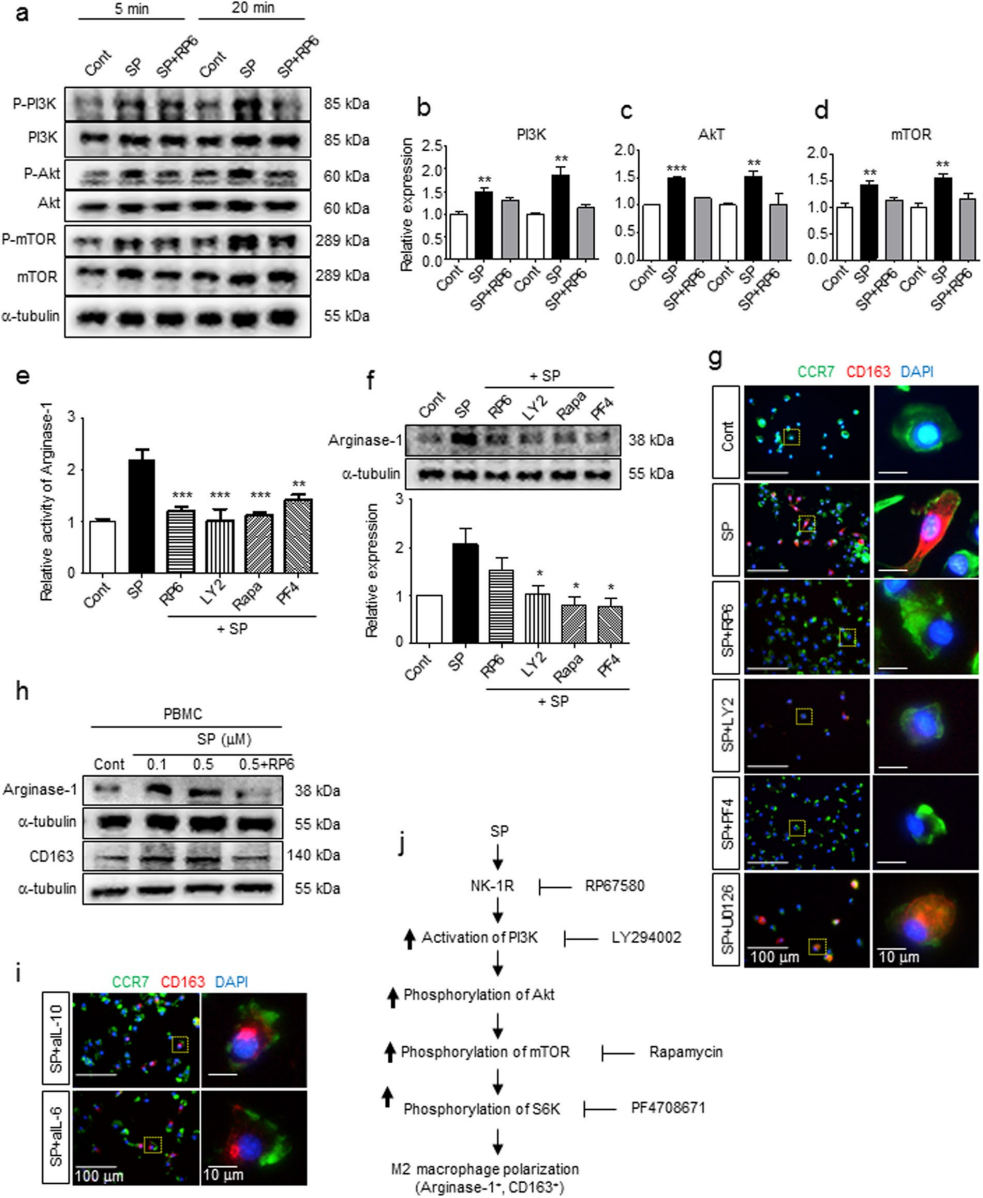
SP activates the NK-1R/PI3K/Akt/mTOR/S6K signaling pathway, leading to the induction of Arginase-1 and CD163 expression. (**a**) Western blot analysis showed that SP-activated PI3K, Akt, and mTOR were detected at 5 min and 20 min. The blockade of NK-1R by pretreatment with RP67580 led to inhibition of PI3K, Akt, and mTOR. The samples derived from the same experiment and full-length blots are presented in Supplementary Figures 19, 20 (n = 3). (**b**–**d**) Relative expression of p-PI3K/PI3K, p-Akt/Akt, and p-mTOR/mTOR was quantified with ImageJ (n = 3). (**e**) All inhibitors, such as the NK-1R antagonist RP67580, PI3K inhibitor LY294002, mTOR inhibitor rapamycin, and S6K inhibitor PF4708671, completely blocked the elevated SP-induced Arginase-1 activity at 6 h (n = 6). (**f**) All inhibitors blocked SP-induced Arginase-1 expression. The western blot results were quantified with ImageJ. The samples derived from the same experiment and full-length blots are presented in Supplementary Figure 21 (n = 3). (**g**) Pretreatment with RP67580 completely blocked CD163 induction in SP-induced M2 at 3 d. Inhibitors of PI3K (LY294002) and S6K (PF4708671), but not MEK/ERK (U0126), prevented the induction of CD163 expression following the administration of SP (Green = CCR7, Red = CD163, Blue = DAPI) (n = 3). (**h**) Rat PBMCs also showed increased expression of Arginase-1 and CD163 at 6 h and 1 d after SP treatment, respectively. Pretreatment with RP67580 blocked the increased SP-induced expression of Arginase-1 and CD163, as demonstrated by western blot analysis. The samples derived from the same experiment and full-length blots are presented in Supplementary Figure 22 (n = 2). (**i**) Treatment of functional blocking antibodies to IL-10 (aIL-10) and IL-6 (aIL-6) failed to block SP-induced CD163 expression at 3 d (Green = CCR7, Red = CD163, Blue = DAPI) (n = 3). (**j**) Schematic representation of Arginase-1^+^CD163^+^ M2 polarization in response to SP via NK-1R/PI3K/Akt/mTOR/S6K activation. Data represent the mean ± SEM (*p < 0.05, **p < 0.01, ***p < 0.001, unpaired t test). Scale bar = 100 μm (**g,i**) and 10 μm (high-magnification image inset in **g,i**).

NK-1R, a G protein-coupled receptor (GPCR), is known to activate PLC-β and, in turn, activate phosphatidylinositol 3 kinase (PI3K)^45–47^. To explore the SP-mediated signaling pathway through NK-1R that results in M2 polarization, PI3K, Akt, and mTOR activation were examined by western blot analysis (Fig. 3a–d). SP immediately stimulated phosphorylation of the PI3K, Akt, and mTOR cascades at 5 min and 20 min, and this effect was blocked by an NK-1R antagonist, RP67580.

Next, downstream signaling linkage of the PI3K/Akt/mTOR pathway to the induction of Arginase-1 and CD163 was investigated by pretreatment with a variety of inhibitors of the signaling cascades (Fig. 3e–g). All inhibitors, such as the NK-1R antagonist RP67580, PI3K inhibitor LY294002, mTOR inhibitor rapamycin, and S6K inhibitor PF4708671, successfully nullified the SP-mediated increase in Arginase-1 activity and protein levels and SP-mediated CD163 expression (Fig. 3e–g). However, a MEK inhibitor, U0126, did not block the effects of SP on CD163-expressing M2 polarization of macrophages, but it significantly reduced the numbers of total macrophages (Fig. 3g). PI3K/Akt/mTOR activation may be mainly involved in SP-induced M2 polarization, but SP-induced ERK activation may be required for the survival and proliferation of macrophages without affecting M2 polarization. In addition, peripheral blood mononuclear cells (PBMCs) also exhibited increased levels of Arginase-1 and CD163 protein in response to SP, which was also blocked by RP67580 pretreatment (Fig. 3h).

Since IL-6 and IL-10 exert similar effects on CD163 expression in human monocytes and macrophages^48^, the potential involvement of other cytokines in SP-induced M2 polarization as a secondary mediator was tested by functional blocking antibodies against either IL-10 or IL-6 along with SP treatment (Fig. 3i). Neither blocking of IL-10 nor IL-6 interfered with SP-mediated induction of CD163 in macrophages. Thus, neither IL-10 nor IL-6 was involved in SP-mediated CD163 expression. Collectively, SP induced Arginase-1 and CD163-expressing M2 macrophages through the NK-1R/PI3K/Akt/mTOR/S6K signal transduction pathway (Fig. 3j).

### SP is a mitogen in macrophages and has a dominant effect on M2 like macrophage polarization even in the presence of the Th1 cytokine IFNγ

**Figure 4:**
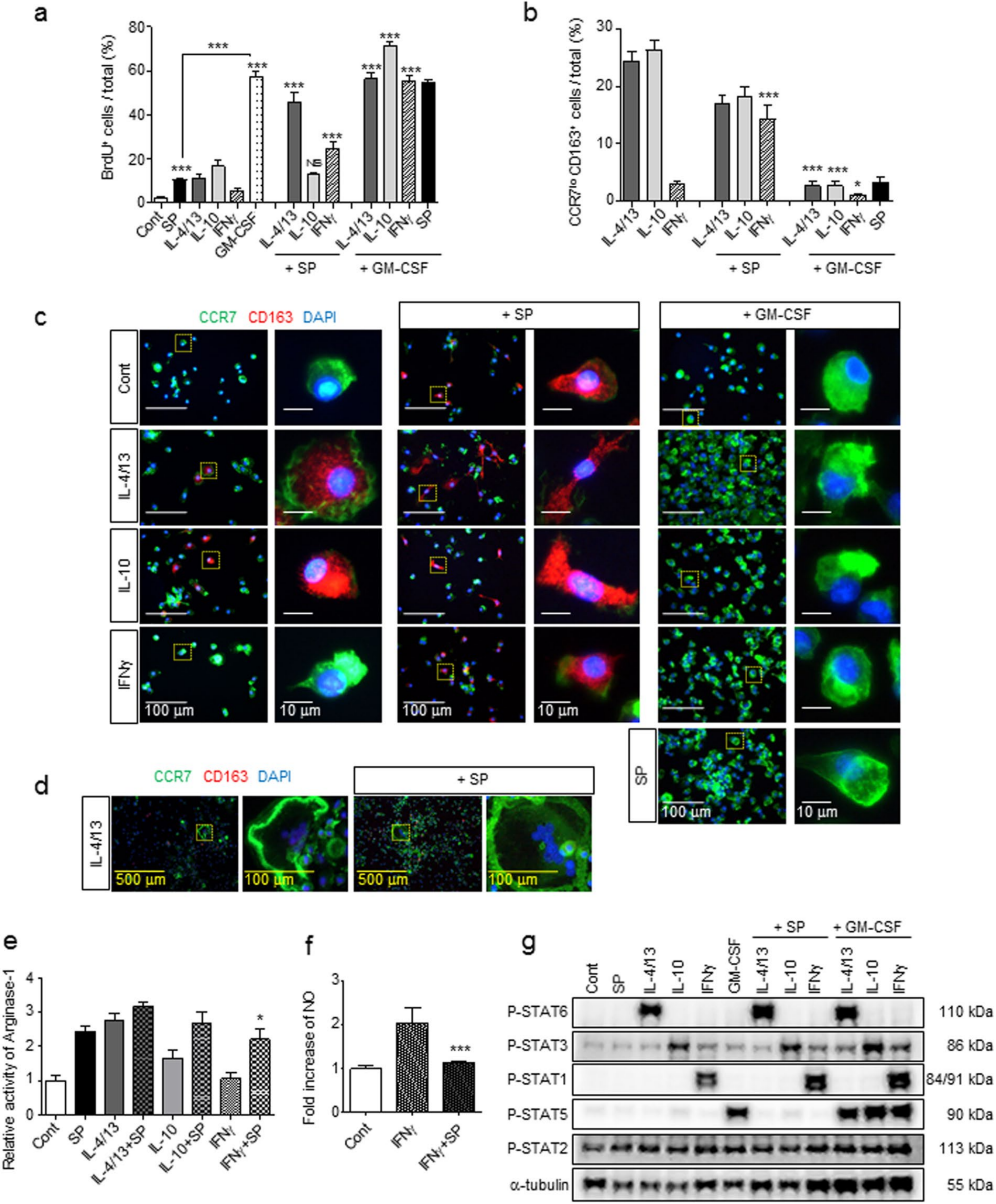
SP is a mitogen for macrophages and affects M2 polarization in a dominant manner even in the presence of the Th1 cytokine INFγ. (**a**) BrdU incorporation analysis revealed that SP was less potent at inducing proliferation than GM-CSF and that SP and GM-CSF had a synergistic effect on proliferation on all types of macrophages at 3 d. Fold change of BrdU incorporation in MΦ^GM-CSF^ was shown in Supplementary Figure 2a. The number of BrdU^+^ cells was counted (5 random fields/coverslip) at 100 × magnification (0.85 mm^2^) (n = 3). (**b**–**c**) The percentage of CCR7^lo^CD163^+^ M2 macrophages was maintained in M2a^IL-4/13^ and M2c^IL-10^ subtypes in the presence of SP but completely diminished after 3 d of GM-CSF treatment. SP-induced M2 macrophages in M1^INFγ^ showed similar proportions of the M2a^IL-4/13^ and M2c^IL-10^ subtypes (Green = CCR7, Red = CD163, Blue = DAPI). The number of CCR7^lo^CD163^+^ M2 cells was counted (5 random fields/coverslip) at 100 × magnification (0.85 mm^2^) (n = 3). (**d**) IL-4/13-induced MGCs were still present in response to SP co-treatment at 3 d (Green = CCR7, Red = CD163, Blue = DAPI) (n = 6). (**e**) The activity of Arginase-1 was increased at 6 h in response to IL-4/13, IL-10 or IFNγ co-treatment with SP, as determined by Arginase-1 activity assay. SP-induced M2 polarization is dominantly working over Th1 cytokines, IFNγ (n = 6). (**f**) The iNOS activity assay revealed a decrease in IFNγ activity after SP co-treatment compared with that after IFNγ treatment at 1 d (n = 3). (**g**) SP did not activate STAT1, STAT3, STAT5, or STAT6, and SP co-treatment did not affect specific cytokine-mediated STAT expression such as STAT6 by IL-4/13, STAT3 by IL-10, STAT1 by IFNγ, and STAT5 by GM-CSF at 6 h. The western blot samples derived from the same experiment and full-length blots are presented in Supplementary Figure 23. (n = 2). Data represent the mean ± SEM (*p < 0.05, ***p < 0.001, unpaired t test). Scale bar = 500 μm (**d**), 100 μm (**c**, high-magnification image inset in **d**) and 10 μm (high-magnification image inset in **c**).

To define the role of SP as a novel cytokine in M2 like macrophage polarization in injured tissue, its effect on cell proliferation was examined in the presence of other Th1 and Th2 cytokines using the BrdU incorporation assay (Fig. 4a and Supplementary Fig. 2a). Withdrawal of GM-CSF in MΦ^GM-CSF^ reduced the number of cells by half in 3-d cultures (Supplementary Fig. 2b,c), suggesting that GM-CSF played a role as a major macrophage mitogen. SP, IL-4/13, IL-10, IFNγ, and GM-CSF increased the numbers of BrdU-incorporating cells in MΦ^GM-CSF^ by approximately 4.7, 4.9, 7.5, 2.4, and 25.6-fold, respectively. Co-treatment with SP and Th1 or Th2 cytokines, such as a combination treatment with IL-4/13 or IFNγ, synergistically increased cell proliferation. Among the tested cytokines, GM-CSF alone or in combination with other cytokines provided the most potent mitogen capacity in macrophages.

To simulate the injured tissue microenvironment, the capacity of SP to stimulate M2 polarization of MΦ^GM-CSF^ in the presence of other Th1 and Th2 cytokines was examined (Fig. 4b,c). Notably, SP activated MΦ^GM-CSF^ to differentiate into M2 macrophages even in the presence of the Th1 cytokine IFNγ. Other Th2 cytokines, such as IL-4/13 and IL-10, seemed to function redundantly in M2 polarization with SP, but none of them stimulated osteoclast differentiation of MΦ^GM-CSF^ based on TRAP staining (Supplementary Fig. 3). Interestingly, most CCR7^−^CD163^+^ M2 macrophages induced by IL-4/13 or IL-10 treatment exhibited an enlarged round shape, but only those co-treated with SP displayed a more elongated shape with ruffles, suggesting an active migrating potential (Supplementary Fig. 4). Furthermore, IL-4/13-induced multinucleated giant cells (MGCs), which typically engulf macromolecules, were not affected by SP and GM-CSF co-treatment (Fig. 4d and Supplementary Fig. 3). Accordingly, SP-mediated M2 like macrophage polarization, which may occur in response to the abundance of pro-inflammatory cytokines in the injured tissue, was distinct from IL-4/13-induced M2a macrophages and MGCs. In contrast, GM-CSF co-treatment completely inhibited Th2 cytokine- and SP-mediated M2 polarization of MΦ^GM-CSF^. GM-CSF was the most potent inducer of M1 macrophages, overriding the action of Th2 cytokines such as IL-4/13 and IL-10 in addition to SP.

To further explore the interplay between SP and IFNγ in M1/M2 polarization of macrophages, Arginase-1 activity and NO production were examined (Fig. 4e,f). Interestingly, IFNγ, a Th1 cytokine, also stimulated Arginase-1 activity in macrophages approximately 2-fold only when combined with SP (Fig. 4e), but it did not generate NO, a typical M1 phenotype (Fig. 4f). Clearly, SP can skew the IFNγ-induced M1 phenotype of macrophages toward the M2 phenotype. However, SP did not activate any STAT1, STAT3, STAT5, and STAT6 signaling, and SP co-treatment did not affect any specific cytokine-mediated STAT expression such as STAT6 by IL-4/13, STAT3 by IL-10, STAT1 by IFNγ, and STAT5 by GM-CSF (Fig. 4g). It is possible that SP-stimulated induction of CD163 and Arginase-1 and suppression of CCR7 and NO production in the presence of IFNγ may occur independently of the cytokine-stimulated STAT pathway.

### M2^SP^ reveal highly phagocytic and endothelial adhesive activity and a distinct cytokine profile

**Figure 5:**
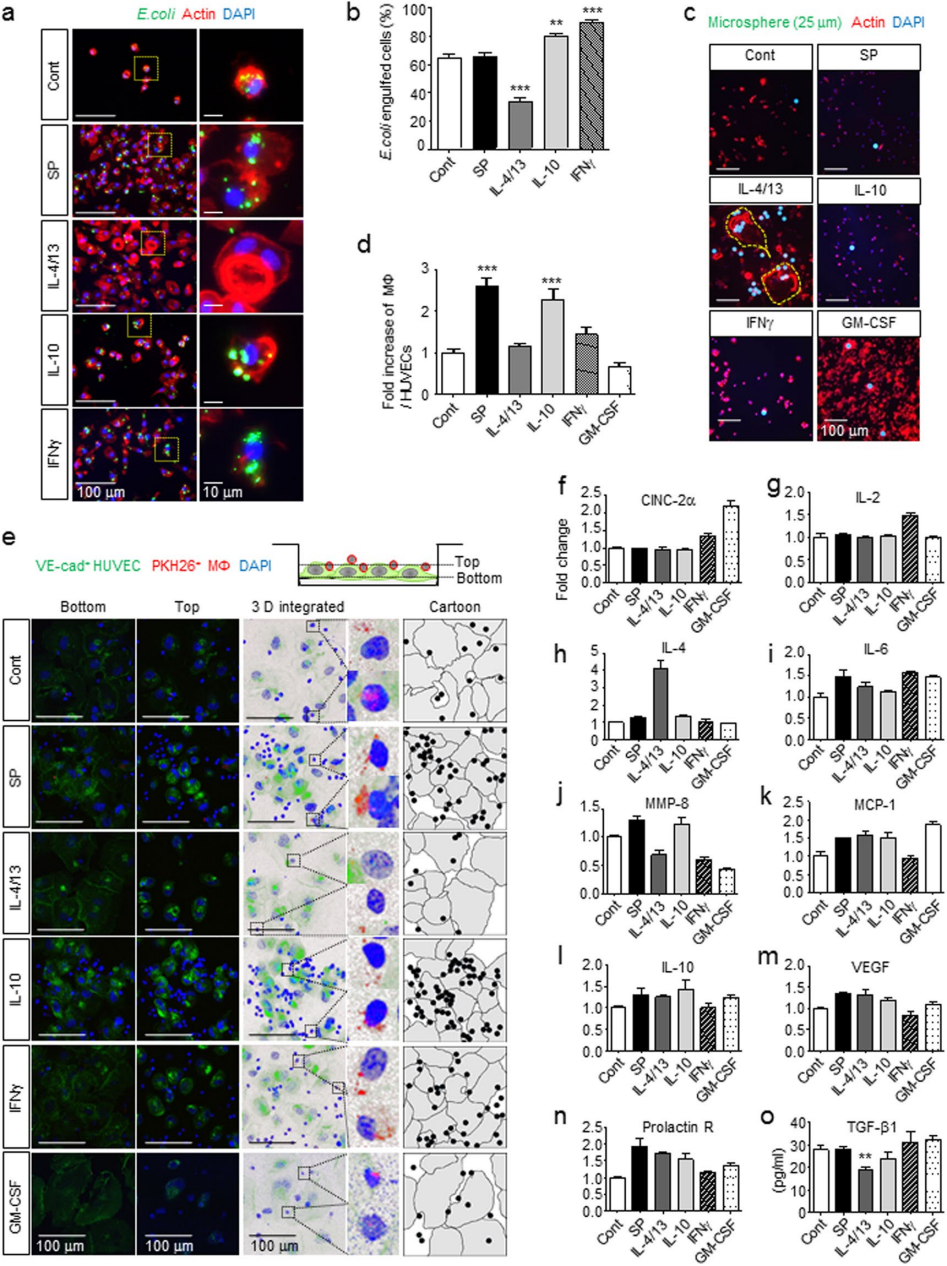
M2^SP^ reveal highly phagocytic and endothelial adhesive activity and a distinct cytokine profile. (**a**,**b**) Anti-microbial activity was determined based on phagocytic activity toward FITC-labeled *E. coli* particles for 30 min. Analysis of *E. coli*-engulfed cells demonstrated strong anti-microbial activity of all types of macrophages, M2^SP^, M2c^IL-10^, and M1^INFγ^-like MΦ^GM-CSF^, except M2a^IL-4/13^ (Green = FITC-*E.coli* particles, Red = Actin, Blue = DAPI). The number of *E. coli*-engulfed cells was counted (10 random fields/coverslip) at 200 × magnification (2.74 mm^2^) (n = 3). (**c**) IL-4/13-induced MGCs engulfed the large microspheres (Green = 25 μm Fluoresbrite microspheres, Red = Actin, Blue = DAPI). Enlarged images were shown in Supplementary Figure 6a and engulfed microspheres were confirmed inside of the MGCs in Supplementary Figure 6b (n = 3). (**d**,**e**) The adhesive activity of M2^SP^ to HUVECs was determined. M2^SP^ demonstrated a high adhesion ability like M2c^IL-10^, which was not detected in M2a^IL-4/13^ (Green = VE-cadherin, Red = PKH26-MΦ, Blue = DAPI). “Bottom” image confirms the VE-cadherin boundary of HUVECs and “Top” image confirms the attachment of macrophages. “3 D integrated image” was acquired from Z stack microscopic images and was presented as cartoons (Gray = HUVECs, Black = MΦ). The number of MΦ and HUVECs was counted (5 random fields/coverslip) at 100 × magnification (0.85 mm^2^) (n = 2). (**f**–**o**) M2^SP^ released IL-6, MMP-8, MCP-1, IL-10, VEGF, and Prolactin R. M2^SP^, M2a^IL-4/13^, and M2c^IL-10^ commonly secreted MCP-1, IL-10, VEGF and Prolactin R after 1 d of treatment. (**f**–**n**) Quantification of cytokine array density was performed with ImageJ (n = 1). (**o**) ELISA (n = 3). Data represent the mean ± SEM (**p < 0.01, ***p < 0.001, unpaired t test). Scale bar = 100 μm (**a**,**c**,**e**) and 10 μm (high-magnification image inset in **a**).

Alternatively activated M2 macrophages are recognized tissue repairing macrophages in a variety of acute tissue injuries or chronic wounds due to their anti-inflammatory effects and active cleanup of dead cells^8–11^. This function may be critical for the transition of the wound microenvironment to become more receptive for tissue repair along with incoming precursor or stem cells. To assess the phagocytic function of macrophage subtypes, SP-induced M2 like macrophages (M2^SP^) were compared with different M2 subtypes, M2a (M2a^IL-4/13^), M2c (M2c^IL-10^) and M1 (M1^IFNγ^ and MΦ^GM-CSF^), using fluorescence-labeled *E. coli* particles, H_2_O_2_-treated PKH-labeled dead brain cells and dead blood cells^49^, and microbeads (25 μm in diameter) (Fig. 5a-c, Supplementary Figs 5–6). MΦ^GM-CSF^, M1^IFNγ^, M2^SP^, and M2c^IL-10^ subtypes showed high phagocytic activity of *E. coli* particles and dead brain and blood cells, suggesting that they were active scavengers of infectious pathogens and dead cells. However, when GM-CSF was continuously supplied to MΦ^GM-CSF^, phagocytic function became deficient in MΦ^GM-CSF^. GM-CSF might maintain actively proliferating NO-producing precursors or an immature state of MΦ^GM-CSF^. Furthermore, M2a^IL-4/13^ did not show high phagocytic activity of *E. coli* particles, but IL-4/13-induced MGCs were able to engulf many 25 μm microspheres (Fig. 5a–c, Supplementary Fig. 6). Taken together, M2^SP^ macrophages exhibited active phagocytic functions toward both bacteria and dead cells, similarly to M1^IFNγ^ and M2c^IL-10^ subtypes, but they were distinct from M2a^IL-4/13^.

To achieve tissue-repairing functions, activated monocytes and macrophages may acquire the capacity to adhere to endothelial cells and exit the injured tissue^50–52^. Macrophages exposed to different cytokines or SP were allowed to adhere to a human umbilical vein endothelial cell (HUVEC) monolayer for 1 h, and the bound macrophages were counted (Fig. 5d–e, Supplementary Fig. 7). Notably, M2^SP^ and M2c^IL-10^ exhibited a strong ability to adhere to endothelial cells. In contrast, M2a^IL-4/13^ and M1^GM-CSF^ showed impaired adhesion to HUVECs. Therefore, along with the high phagocytic function of dead cells and microorganisms, M2^SP^ and M2c^IL-10^ were expected to be tissue-repairing macrophages.

To further define and classify the role of SP in M1/M2 polarization of macrophages, a cytokine array was performed (Fig. 5f-n). IFNγ increased the levels of the Th1 cytokines CINC-2α, IL-2, and IL-6, but IL-4 alone markedly stimulated IL-4 expression. However, neither SP nor IL-10 increased the levels of Th1 cytokines such as CINC-2α and IL-2, but they increased MMP-8, MCP-1, IL-10, VEGF, and Prolactin R, which are important for chemotactic migration and angiogenesis functions of macrophages. TGF-β1 secretion was not affected by SP treatment but was suppressed by IL-4/13 (Fig. 5o). Based on the cytokine expression profiles, M2^SP^ may be similarly classified to the M2c^IL-10^ subtype, supporting a characterization of tissue-repairing macrophages.

### SP can activate bone marrow-derived monocytes to express M2 phenotype

**Figure 6:**
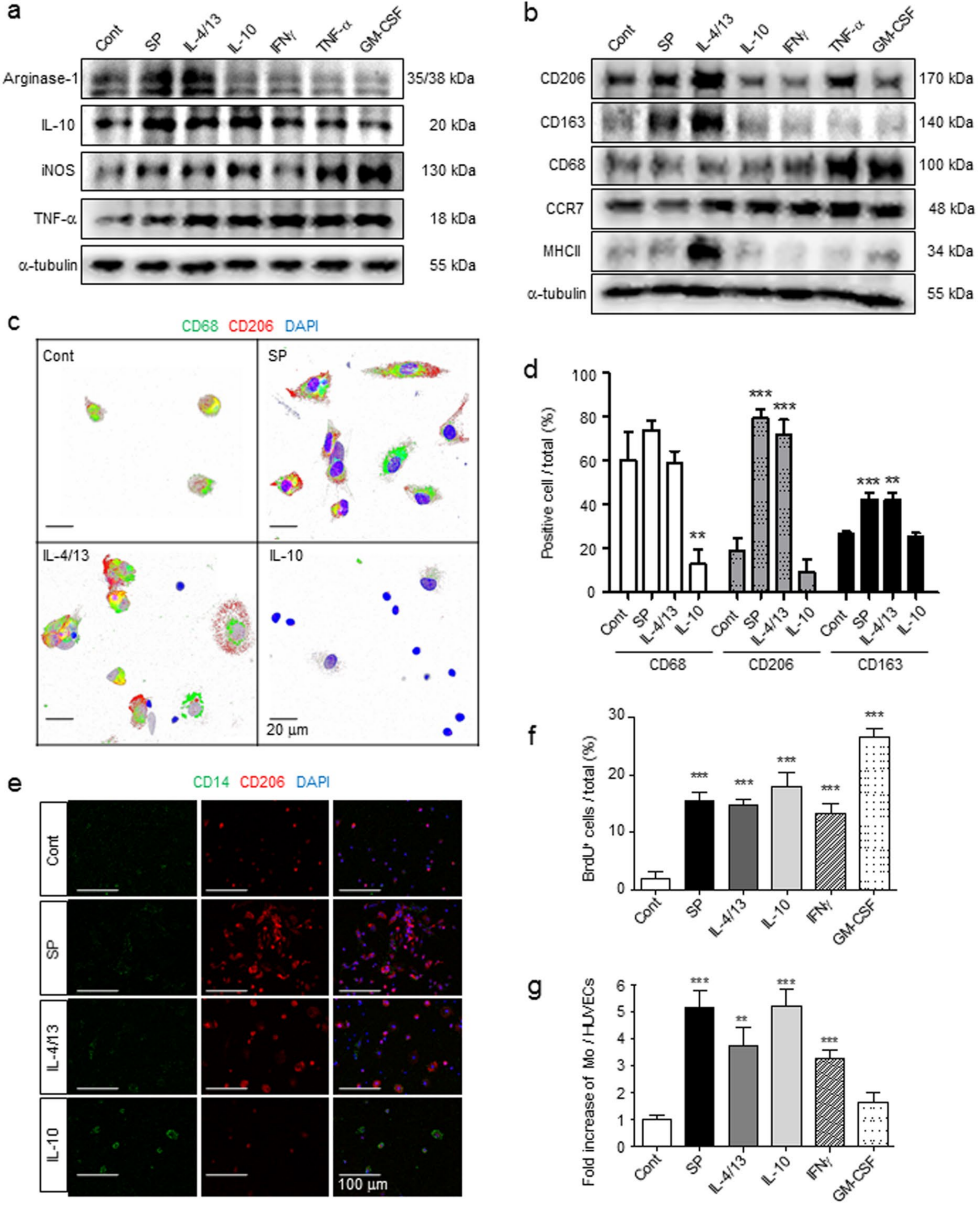
SP can activate bone marrow-derived monocytes to express an M2 phenotype. (**a**) Western blot analysis showed that SP increased the expression of Arginase-1 and IL-10 but not the expression of iNOS or TNF-α in Mo^BM^ after 6 h of treatment. The samples derived from the same experiment and full-length blots are presented in Supplementary Figure 24. (n = 2). (**b**) SP and IL-4/13 increased the expression of CD206 and CD163 but not the expression of CD68 or CCR7 after 1 d of treatment. Only IL-4/13 Strongly increased the expression of MHCII. The samples derived from the same experiment and full-length blots are presented in Supplementary Figure 25. (n = 2). (**c**) Immunofluorescent staining showed that Mo^BM^ differentiated into CD68^+^CD206^+^ macrophages following SP and IL-4/13 treatment at 3 d (Green = CD68, Red = CD206, Blue = DAPI). SP-primed monocytes had an elongated morphology based on confocal microscope Z stack section analysis. (**d**) IL-10 blocked differentiation into CD68^+^-activated macrophages, but SP and IL-4/13 induced CD68^+^CD206^+^CD163^+^ M2-phenotype macrophages at 3 d. For the immunofluorescence staining results (Fig. 6c and Supplementary Fig. 8), the number of positive-cells was counted (5 random fields/coverslip) at 100 × magnification (0.85 mm^2^) (n = 3). (**e**) Immunofluorescent staining showed that Mo^BM^ were maintained as CD14^+^ monocytes only by IL-10 treatment at 3 d (Green = CD14, Red = CD206, Blue = DAPI) (n = 3). (**f**) BrdU incorporation assays revealed a similarly potent proliferating ability of SP to that of cytokines, but a less potent proliferating ability in comparison to GM-CSF. The number of BrdU^+^ cells was counted (5 random fields/coverslip) at 100 × magnification (0.85 mm^2^) (n = 3). (**g**) Mo^SP^ demonstrated high adhesion ability to HUVECs, and eventually were highly migratory like Mo^IL-10^. The numbers of MΦ and HUVECs were counted (5 random fields/coverslip) at 100 × magnification (0.85 mm^2^) (n = 2). Data represent the mean ± SEM (**p < 0.01, ***p < 0.001, unpaired t test). Scale bar = 20 μm (**c**) and 100 μm (**e**).

Since SP activated the inflammatory macrophages MΦ^GMCSF^ to develop the M2 phenotype *in vitro*, it was expected to also exert M2 priming in Mo^BM^. Both SP and IL-4/13 significantly increased the expression of early M2 markers such as Arginase-1 and IL-10 in Mo^BM^ at 6 h after treatment (Fig. 6a) and of M2 surface markers such as CD206 and CD163 at 1 d, based on western blot analysis (Fig. 6b) and immunofluorescence staining (Fig. 6c,d and Supplementary Fig. 8). Only IL-4/13 specifically induced MHCII expression, supporting its role in the enhancement of antigen-presenting capacity. In contrast, TNF-α and GM-CSF clearly increased early M1 markers such as iNOS instead of Arginase-1 induction and increased activated marker, CD68 and M1 surface marker, CCR7. However, IL-10 did not have a prominent effect on the phenotypic change in Mo^BM^, excluding IL-10 and TNF-α, but it sustained a small round monocyte morphology (Fig. 6c, Supplementary Fig. 8b,c) and the CD14 monocyte marker (Fig. 6e). SP again revealed a mitogen potential similar to IL-4/13, IL-10, and IFNγ, which was lower than that of GM-CSF based on the BrdU incorporation assay (Fig. 6f). Markedly, SP enhanced the endothelial cell-adhesion capacity of Mo^BM^, demonstrating a much higher level than that of untreated or GM-CSF-treated Mo^BM^ (Fig. 6g). Thus, SP enables Mo^BM^ to differentiate into an M2-skewed phenotype by functioning as a mitogen and an M2-polarzing cytokine, which is distinct from the known M2 cytokines IL-10 and IL-4/13.

This capacity of SP to induce M2 polarization was further investigated in the human leukemia monocyte cell line THP-1 and PMA-primed macrophages (THP-1 MΦ) (Supplementary Figs 9–11). SP increased Arginase-1 expression in THP-1 cells and THP-1 MΦ in a dose-dependent manner, which was consistently blocked by co-treatment with NK-1R blocker (Supplementary Fig. 9). SP further stimulated the PI3K/Akt/mTOR signaling pathway in THP-1 cells (Supplementary Fig. 10) and THP-1 MΦ (Supplementary Fig. 11).

### SP stimulates peripheral mobilization of CD11b^+^CD206^+^ cells, possibly from the bone marrow, which may contribute to the infiltrating CD11b^+^CD206^+^ cells during early-stage SCI

**Figure 7:**
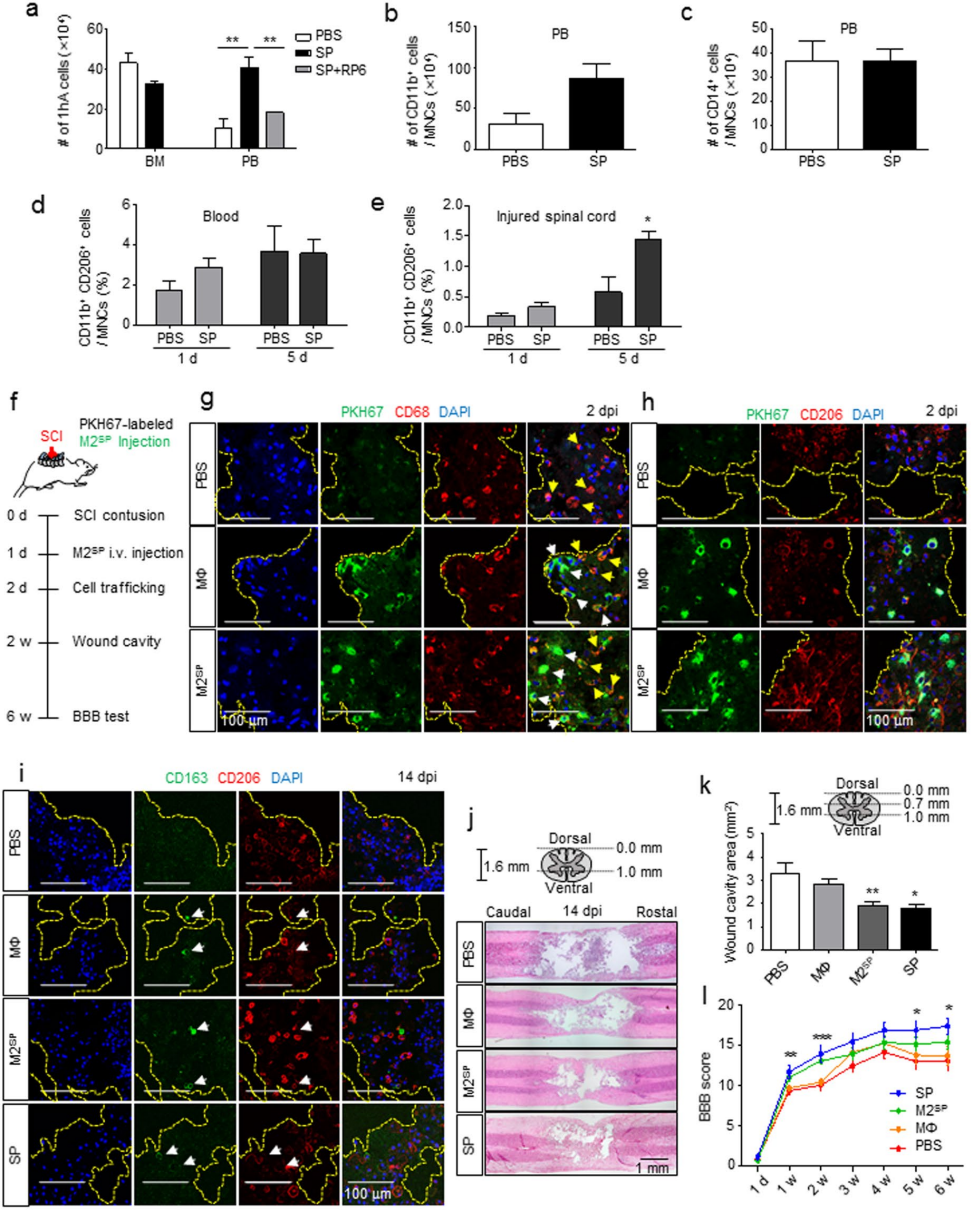
SP stimulates peripheral mobilization of CD11b^+^CD206^+^ cells from bone marrow and M2^SP^ adoptive transfer improves functional recovery from SCI. (**a**) At 4.5 h after SP-injection, the number of 1-h adherent cells from the blood had increased. RP67580 blocked the increased number of 1-h adherent cells in PBMCs (n = 3). (**b**) At 4.5 h after SP-injection, MACS analysis showed that CD11b^+^ cells had increased in the blood (n = 3). (**c**) At 4.5 h after SP-injection, MACS analysis showed that the number of CD14^+^ cells did not differ in the blood (n = 3). (**d**) At 1 dpi, CD11b^+^CD206^+^ cells among the SP-injected PBMCs increased by 1.7-fold compared with that after PBS-injection. At 5 d, CD11b^+^CD206^+^ cells in response to SP-treatment was similar to that after PBS injection (n = 3). (**e**) At 1 dpi, SP treatment increased CD11b^+^CD206^+^ cells in injured spinal cord by 1.7-fold compared with that after PBS treatment. At 5 dpi, the number of CD11b^+^CD206^+^ macrophages increased by 2.5-fold in response to SP compared with that after PBS injection (n = 3). (**f**) A schematic of the procedure used for the adoptive transfer of M2^SP^ in a rat SCI. PKH67-labeled MΦ and M2^SP^ were injected at 1 dpi. (**g**) At 2 dpi, PKH67-labeled cells were observed at the injury core-lesion site. Longitudinal tissue sections were chosen at a distance of ~ 700 μm from the dorsal surface of the injury site. White arrows represent the PKH67^+^CD68^+^ adoptive transferred cells and yellow arrows represent PKH67^−^CD68^+^ resident microglia (Green = PKH67, Red = CD68, Blue = DAPI) (n = 3). (**h**) At 2 dpi, PKH67-labeled cells co-localized with CD206 (Green = PKH67, Red = CD206, Blue = DAPI) (n = 3). (**i**) At 14 dpi, CD163^+^CD206^+^ cells were observed in the MΦ, M2^SP^ and SP groups but not in the PBS group (Green = CD163, Red = CD206, Blue = DAPI) (n = 3). (**j**,**k**) Histology and quantitative analysis of the lesion cavity delineated by H&E staining at 14 dpi. Every 2 stained tissue samples per rat were chosen at a distance of 0.7 mm and 1.0 mm from the dorsal section and lesion cavity was analyzed using ImageJ (PBS, n = 8; MΦ, n = 8; M2^SP^, n = 8; SP, n = 3). (**l**) The BBB score was evaluated for 6 weeks (1 d ~ 2 w- PBS, n = 14; MΦ, n = 14; M2^SP^, n = 15; SP, n = 5/3 w ~ 6 w- PBS group, n = 6; MΦ, n = 6; M2^SP^, n = 6; SP, n = 5). Data represent the mean ± SEM (*p < 0.05, **p < 0.01, ***p < 0.001). (**a-e,k**) Unpaired t test. (**l**) one-way ANOVA. Scale bar = 100 μm (**g**–**i**) and 1 mm (**j**).

The role of SP in M2 polarization of monocytes and macrophages and their endothelial cell adhesion capacity led us to hypothesize that SP might affect bone marrow monocyte trafficking and infiltration to the injured tissue, thereby acting as tissue-repairing macrophages. As reported previously^32, 33^, SP treatment immediately after SCI increased the numbers of IL-10^+^CD11b^+^ cells in the lesion site at 1 d, reduced the numbers of apoptotic cells, and improved functional recovery compared with the non-injected control. Thus, whether SP can regulate the monocyte pool between the bone marrow and blood was explored (Fig. 7a–c). At 4.5 h after SP injection, one-hour adherent cells, mostly representing CD11b^+^ myeloid cells, were reduced in the bone marrow but increased in the blood approximately three-fold compared with those in the non-injected control, and this effect was inhibited by co-injection of an NK-1R blocker, RP67580 (Fig. 7a). In the CD11b and CD14 MACS analysis, CD11b^+^ cells increased approximately three-fold (Fig. 7b) in the blood, but there were no changes in CD14 monocytes (Fig. 7c), suggesting that SP enhanced CD11b^+^CD14^−^ cell trafficking from the bone marrow to the blood.

Next, whether SP can also affect CD11b^+^ cell trafficking in the SCI was examined (Fig. 7d,e). SP injection immediately after SCI increased the numbers of CD11b^+^CD206^+^ cells in the blood approximately 1.7-fold than PBS injection at 1 d (Fig. 7d), which was reflected by the similar increase in CD11b^+^CD206^+^ cells in the SCI lesion site at 1 d (Fig. 7e). At 5 d, approximately 2.5-fold more CD11b^+^CD206^+^ cells were detected in SP-injected SCI rats than in PBS-injected rats, even though similar levels of CD11b^+^CD206^+^ cells were detected in the blood. Accordingly, SP stimulated peripheral mobilization of CD11b^+^CD206^+^ cells during early-stage SCI, which may infiltrate the SCI lesion site and persist longer therein than in the PBS-injected control.

### Intravenously injected M2^SP^ is detected in the SCI lesion site and improves functional recovery from SCI, similarly to SP-injected SCI

To evaluate the role of SP-stimulated peripherally mobilized CD11b^+^CD206^+^ cells in tissue repair, adoptive cell therapy using PKH67-labeled MΦ and M2^SP^ was performed at 1 d post-SCI (Fig. 7f–l). Although both PKH67-labeled MΦ and M2^SP^ were clearly detected in the SCI lesion at 2 d post-SCI, PKH67-labeled M2^SP^ were more frequently detected, which was co-stained with CD68 (Fig. 7g) and CD206 (Fig. 7h). Those SCI-infiltrating M2^SP^ may play crucial roles in anti-inflammatory and wound-healing effects in the lesion site. At 2 d post-SCI, more MAP2^+^ neuron and NeuN^+^TUNEL^−^ neuron were detected in M2^SP^ adoptive transfer, similar to that of SP-injected one (Supplementary Figs 12 and 13). At 14 d post-SCI, more CD206^+^ cells were detected in the lesion, and a smaller wound cavity were detected in M2^SP^-transfused than in MΦ-transfused SCI (Fig. 7i–k, Supplementary Figs 14 and 15). The wound cavity was 3.29 ± 0.45 mm^2^ in PBS-injected, 2.84 ± 0.22 mm^2^ in MΦ-transfused, 1.92 ± 0.14 mm^2^ in M2^SP^-transfused, and 1.78 ± 0.18 mm^2^ in SP-injected animals (Fig. 7j,k). To assess the functional recovery of the hind limb, BBB scores were measured weekly (Fig. 7l). Differences between MΦ and M2^SP^ transfusion were first detected at 1 w post-SCI, and they were maintained up to 6 w post-SCI in escalating order of PBS-injected, MΦ-transfused, M2^SP^-transfused, and SP-injected SCI. Therefore, adoptively transferred M2^SP^ cells infiltrated into the injured spinal cord and might participate in the recovery of SCI, which may be expected based on the SP-stimulated peripheral mobilization of M2-skewed monocytes possibly from the blood and bone marrow.

## Discussion

In the present study, we demonstrated that SP can directly induce a phenotypic change in GM-CSF-activated inflammatory macrophages, promoting their differentiation toward unique phagocytic, tissue-repairing M2 macrophages, which are distinct from IL-4/13-induced M2a macrophages and IL-10-induced M2c macrophages. SP-mediated M2 polarization was initiated by the specific SP receptor, NK-1R, followed within 20 min by the PI3K/Akt/mTOR/S6kinase pathway, which led to Arginase-1 induction at 3 h post-SP treatment and CD163/CD206 expression, all of which were accomplished without Th1 and Th2 cytokine-mediated STAT activation and nullified by pretreatment with an NK-1R blocking antagonist and signaling inhibitors. Clearly, SP, which was previously known as a nociceptive neurotransmitter in sensory nerves, plays a role as a novel M2 cytokine with mitogenic capacity. We defined these SP-induced M2 like macrophages as M2^SP^, and they are distinct from M2a^IL-4/13^ and M2c^IL-10^ (summarized in Supplementary Table 1). M2^SP^ shares most of the features of M2a^IL-4/13^ except STAT6 activation, MGC formation, and the capacity for phagocytosis/endothelial adhesion, but it seems to be functionally more similar to M2c^IL-10^, which is related to tissue-repairing macrophages. In particular, SP-induced M2 polarization had a dominant effect over the Th1 cytokine IFNγ, which is abundant in an inflammatory tissue environment. Thus, we propose that SP could be developed as pharmacological mediator to manipulate the tissue injury microenvironment and the chronic inflammatory microenvironment associated with type 2 diabetes and atherosclerosis.

As explored in this study, STAT activation does not seem to be contingent on M2 polarization. SP induced most phenotypes of M2 macrophages without STAT activation (Figs 2 and 4). Moreover, co-treatment with SP and the Th1 cytokine IFNγ preserved IFNγ-mediated STAT1 activation but successfully converted MΦ^GM-CSF^ to M2-phenotype macrophages (Fig. 4), possibly through the dominant effect of SP over IFNγ. Furthermore, GM-CSF co-treatment with IL-4/13 or IL-10 completely abolished Th2 cytokine-induced M2 polarization, with GM-CSF having a dominant effect over IL-4/13 and IL-10 but preserved IL-4/13 activation of STAT6, IL-10 activation of STAT3, and GM-CSF activation of STAT5 in the co-treatment (Fig. 4). Therefore, the cytokine-specific JAK/STAT pathway may not be crucial, at least to acquire the M2 phenotype, as defined by increased expression of Arginase-1 and CD163, and to lose the M1 phenotype defined by CCR7. Of note, ERK activation may not be essential for acquisition of the M2 phenotype because the MAPK inhibitor did not abolish SP-mediated M2 polarization but rather reduced cell proliferation. Collectively, SP directly induced M2 polarization based on the expression of Arginase-1, CD163, and CD206 in MΦ^GM-CSF^ through the NK-1R/PI3K/Akt/mTOR/S6kinase pathway without STAT activation.

Arginine usage in the cell may be an early decision-making step for SP-induced M2 polarization. Upon SP treatment of MΦ^GM-CSF^, Arginase-1 activity increased as early as 3 h and was much higher at 6 h; however, it was completely down-regulated to basal levels at 24 h post-SP treatment. This SP-mediated Arginase-1 activity and its expression were increased in a dose-dependent manner and inhibited by an NK-1R antagonist and by inhibitors of the PI3K/Akt/mTOR/S6kinase pathway, suggesting that Arginase-1 is a downstream effector molecule in the SP signaling pathway. In contrast, Th1 cytokines such as IFNγ and GM-CSF did not induce Arginase-1 but instead increased NO production. It is likely that the Arginase-1-mediated conversion of arginine to ornithine, which is substrate for polyamine synthesis, may be an early requirement for the anti-inflammatory response and resolution of inflammation in M2 macrophages. This phenomenon is probably incompatible with the usage of arginine for NO production via iNOS, a hallmark of inflammatory M1 macrophages^42–44^. This notion was also supported by the results showing that SP co-treatment with IFNγ blocked IFNγ-mediated NO production but concomitantly increased Arginase-1 activity (Fig. 4e,f). Thus, Arginase-1 might play a critical role in macrophage behavior, either eliciting cytotoxic activity via NO^5–7, 42–44^ or terminating tissue-destructive inflammation in the injured tissue and the production of polyamines, which are essential for tissue repair, cell proliferation, and collagen synthesis^42–44^.

M2^SP^ seems to be more active in cell motility and endothelial transmigration than any other M2 subtype. M2^SP^ had a more elongated morphology with ruffles. M2^SP^s were approximately 2–3-fold longer than macrophages of the M2a, M2c, M1, and MΦ^GM-CSF^ subtypes, and they all had a round shape (Supplementary Fig. 4). This effect was preserved even in the presence of other Th2 cytokines. As demonstrated by the highly adhesive capacity of M2^SP^ onto the endothelial cell monolayer (Fig. 5e), M2^SP^ may be more versatile in endothelial transmigration and tissue infiltration during egress from bone marrow and extravasation to the injured tissue. Importantly, SP injection increased the numbers of CD11b^+^ cells and one-hour adherent cells, possibly representing monocytes, by approximately three-fold in the peripheral blood at 4.5 h in normal rats (Fig. 7a,b). Concomitantly, it decreased the levels of these cells in the bone marrow, which suggested that SP stimulated monocyte egress from the bone marrow. Furthermore, SP injection immediately after SCI increased the numbers of CD11b^+^CD206^+^ cells in the blood and the SCI lesion site at 1 d, strongly supporting the occurrence of SP-stimulated monocyte recruitment to the injured tissue. The mechanism involved in SP-stimulated monocyte trafficking was not clearly elucidated in the present study. However, blockade of the increase in monocytes in the blood using the NK-1R antagonist and successful detection of intravenously transfused PKH67-labeled M2^SP^ in the SCI lesion site along with its improved functional recovery in the SCI rat strongly support a positive role of SP in monocyte trafficking toward the injured site and a subsequent beneficial effect on tissue repair. M2^SP^ adoptive transfer increased MAP2^+^ neuron and NeuN^+^TUNEL^−^ neuron survival at 2 dpi, reduced wound cavity at 14 dpi and enhance hind limb functional recovery by open field behavior test for 6 w similar to SP-injected group.

**Figure 8:**
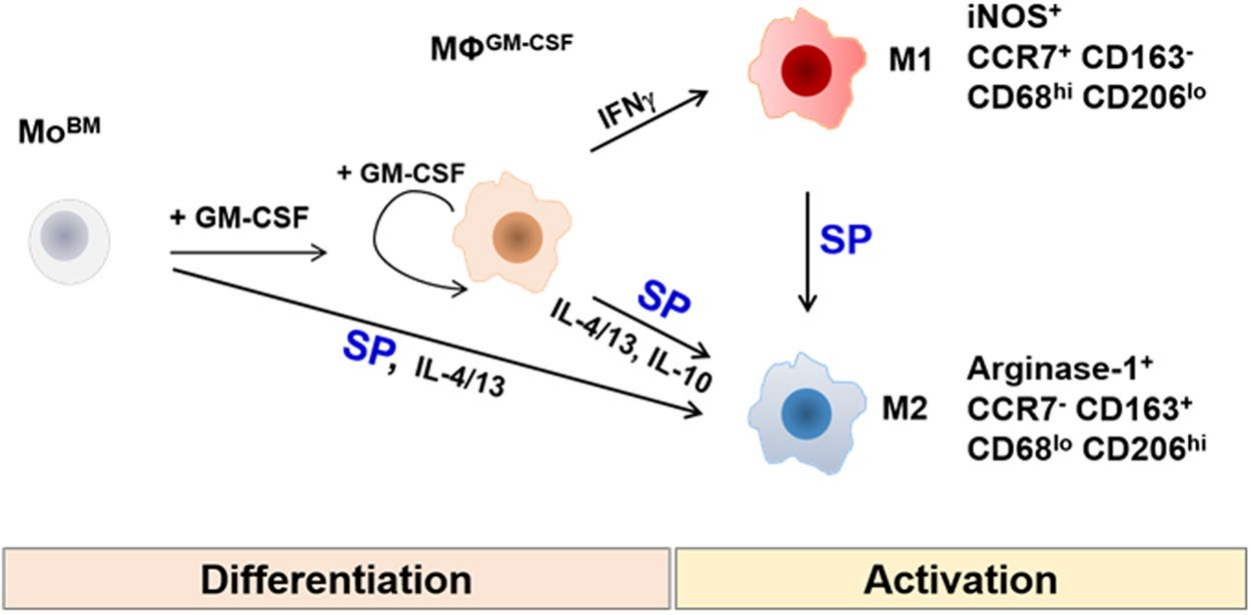
Summary of the characteristics of M2^SP^ compared with other M2 and M1 subtypes.

Cytokines seem to function in a hierarchical manner during the differentiation of monocytes into macrophages. MΦ^GM-CSF^, which are GM-CSF-differentiated macrophages from bone marrow monocytes, are highly proliferating activated macrophage precursors, which can be fully differentiated into pro-inflammatory M1 macrophages in response to Th1 cytokine IFNγ treatment or into M2 macrophages in response to treatment with the Th2 cytokine IL-4/13 and IL-10 in addition to SP (Fig. 8). MΦ^GM-CSF^ expressed most of the activated macrophage surface markers, such as CD68 and CCR7, completely lost the monocyte marker CD14, and displayed rapid self-renewal capacity. However, they did not acquire obvious cell debris phagocytic capacity. Among the tested cytokines, GM-CSF was the most potent mitogen for monocytes and macrophages, and its withdrawal resulted in cell death. Thus, GM-CSF may be commonly required for monocyte differentiation into activated precursors of M1 and M2 macrophages, but its sustained presence may inhibit specific M1/M2 differentiation. However, as shown in monocytes and THP-1 cells (Fig. 6, Supplementary Figs 8–11), SP or IL-4/13, without GM-CSF priming of the MΦ^GM-CSF^ precursor, directly induced M2 polarization from monocytes, as confirmed by the expression levels of Arginase-1, IL-10, CD163, and CD206. MHCII expression was detected only in IL-4/13-induced and not in SP-induced macrophages, clearly suggesting that M2a^IL-4/13^ was alternatively activated in macrophages with an antigen-presenting capacity distinct from M2^SP^. Additionally, IL-10 induced IL-10 but did not induce the expression of M2 markers such as Arginase-1, CD206, and CD163. Furthermore, it did not down-regulated expression of the monocyte marker CD14. Therefore, IL-10, which is known to induce deactivation, did not seem to influence monocytes but induced M2c^IL-10^ only after GM-CSF priming to MΦ^GM-CSF^. In addition, the sustained presence of GM-CSF blocked IL-4/13-stimulated M2 polarization, but it did not block IL-4/13-induced MGC formation, a unique function of M2a that is necessary for the uptake of macromolecules. Collectively, SP and IL-4/13 may induce the entire M2 phenotype repertoire in monocytes and GM-CSF-differentiated macrophages, but GM-CSF is required for the self-renewing capacity of the MΦ^GM-CSF^ precursor phenotype, and its removal may lead to the perception of M1/M2-inducing signals. IL-10 may function in M2c polarization, possibly only in MΦ^GM-CSF^, resulting in the deactivation of inflamed macrophages.

Pharmacological regulation of the M1/M2 balance by SP may represent new targets for the treatment of a variety of conditions, including metabolic diseases such as type 2 diabetes and atherosclerosis, chronic non-healing wounds such as diabetic ulcers, devastating acute CNS injury such as that caused by SCI and stroke, HIV infection, and cancer^53–58^. A high M1/M2 ratio in adipose tissue, which plays a key pathogenic role in the development of obesity-induced insulin resistance, could be manipulated to a more M2-enriched microenvironment by SP or adoptive transfer of M2^SP^. The beneficial role of SP in healing has been described in traumatic CNS injury^32, 33^ and in diabetic ulcers^53^, and it may be a consequence of the reduced tissue damage through mitigation of the mounting inflammatory response. Previously, we reported that SP is an injury-inducible messenger that mobilizes CD29^+^ MSC-like cells from the bone marrow^59^. These SP-mobilized MSCs may, in turn, facilitate the immunosuppressive role of SP or M2 polarization during tissue injury. However, in this study, the direct role of SP in M2 polarization of monocytes and macrophages, without the aid of MSCs, was clearly demonstrated, even though it is possible that SP-mobilized MSCs participation in healing and immune control in the injury. However, SP antagonism may also be considered in HIV infection and tumors since SP facilitates HIV infection of macrophages by inducing CD163 expression^57, 58^, and tumor associated macrophages are more like M2-phenotype macrophages, which could be strategically blocked by an NK-1R antagonist. In conclusion, SP is a novel cytokine that induces the differentiation of anti-inflammatory, phagocytic, tissue-repairing M2 like macrophages, which may be considered in the near future in treatments to regulate a variety of tissue inflammation-related diseases and acute injury.

## Methods

### Animals

All animal experiments were approved by the Animal Studies Committee of Kyung Hee University (KHU #10-006) in Yong In, Korea, and performed under the Institutional Animal Care and Use Committee (IACUC) guidelines. Adult male Sprague-Dawley rats (250 g, 7 w) were purchased from Narabiotec.

### Rat monocyte-derived macrophage culture

Bone marrow mononuclear cells were obtained from femurs by flushing and suspended in RPMI1640 medium (Gibco) containing 10% FBS (Gibco) and 1% penicillin streptomycin (Welgene). 2 × 10^7^ cells ml^−1^ and 1 × 10^6^ cells ml^−1^ total mononuclear cells were plated in 6-well and 24-well plates, respectively, and allowed to adhere for 1 h at 37 °C. After washing three times with PBS, monocytes (Mo^BM^) were enriched on the plates. The adherent monocytes started to differentiate into macrophages after culturing with 10 ng ml^−1^ rat recombinant GM-CSF (R&D Systems) for 5 d in complete medium. At 2 d, the medium was replaced with fresh complete medium containing GM-CSF for another 3 d. After 5 d, the purity of the macrophages was routinely >98%, CD11b^+^ cells was 99%, CD68^+^ cells was 98.5% and CCR7^+^ cells was 99.6%. They were referred to as MΦ^GM-CSF^. MΦ^GM-CSF^ were activated by treatment with either SP (0.1 μM, Calbiochem), IL-4 (10 ng ml^−1^, R&D Systems), IL-13 (10 ng ml^−1^, R&D Systems), IL-10 (10 ng ml^−1^, R&D Systems), IFNγ (20 ng ml^−1^, R&D Systems), TNF-α (10 ng ml^−1^, Sigma-Aldrich), LPS (1 μg ml^−1^, Sigma-Aldrich) or GM-CSF (10 ng ml^−1^, R&D Systems) for 3 d.

### Immunofluorescent staining

Cells and tissues were fixed in 3.7% formaldehyde and blocked with 5% skim milk in PBS containing 0.2% Triton X-100 (Sigma-Aldrich) and exposed to the following primary antibodies at 4 °C overnight: CD14 (R&D Systems, MAB3822, 1:200), CD11b (AbD Serotec, MCA275R, 1:200), CD68 (Millipore, MAB1435, 1:200), CCR7 (Abcam, ab32527, 1:1000), CD206 (Abcam, ab64693, 1:200), CD163 (AbD Serotec, MCA342R, 1:100), MHCII (AbD Serotec, MCA46R, 1:200), and NK-1R (Novusbio, NB300-101, 1:50). The secondary antibodies were goat anti-rabbit Alexa 488 (Jackson ImmunoResearch, 1:500), goat anti-mouse Alexa 488 (Jackson ImmunoResearch, 1:500), goat anti-rabbit Cy3 (Jackson ImmunoResearch, 1:500), and goat anti-mouse Cy3 (Jackson ImmunoResearch, 1:500). The cells were mounted with Vectashield mounting medium containing DAPI (Vector Laboratories). The results were observed under a Leica CTR 4000 fluorescence microscope (Leica Microsystems) or a Zeiss LSM 510 META confocal microscope (Carl Zeiss). Each experimental condition was performed independently in triplicate. The number of positive cells were counted (5 random fields/coverslip) at 100 × magnification (0.85 mm^2^) using the Adobe Photoshop CS6 program.

### Arginase activity assay

The QuantiChrom arginase assay kit (BioAssay Systems, # DARG-200) was used to measure the activity of arginase according to the manufacturer’s instructions. 40 μl of the cell lysate was co-incubated with 10 μl of 5 × L-arginine substrate buffer for 2 h at 37 °C. To stop the reaction and calculate the final urea concentrations, 200 μl of urea reagent was added and incubated for 60 min at RT. The OD was measured at 450 nm. The OD of 10 μl of 5 × L-arginine substrate buffer (OD_blank_), 40 μl of sample without substrate buffer (OD_sample_), 50 μl of H_2_O as a standard background (OD_background_) and 50 μl of 1 mM urea standard (OD_standard_) were obtained. The arginase activity (U/liter) was calculated as 1 arginase unit = OD_sample_ − OD_blank_/OD_standard_ − OD_background_ × [urea standard] × 50 × 10^3^/(40 × t). One unit of arginase converts 1 μmol of L-arginine to ornithine and urea per min. Each experimental condition was performed independently in triplicate.

### iNOS activity assay (measurement of NO production)

The concentrations of nitrite (NO_2_^−^) were measured using Griess reagent system (Promega Corporation, # TB229) according to the manufacturer’s instructions. Supernatants were collected at 1 d after SP or cytokine treatment. 50 μl of the sulfanilamide solution was added to 50 μl NO_2_^−^ standard and 50 μl of conditioned medium for 10 min at RT. Then, 50 μl of the NED was added and incubated for 10 min at RT. The OD was measured at 550 nm. Each experimental condition was performed independently in triplicate.

### Western blotting

The cells were lysed with lysis buffer (Cell Signaling Technology, #9803) containing 2 mM PMSF. Lysates were separated by SDS-PAGE and subjected to Western blotting using the following antibodies: Arginase-1 (M-20, Santa Cruz BioTechnology, sc-18355, 1:5000), IL-10 (R&D, AF519, 1:1000), CD206 (Abcam, ab64693, 1:2000), CD163 (AbD Serotec, MCA342R, 1:1000), CD68 (Millipore, MAB1435, 1:2000), CCR7 (Abcam, ab32527, 1:10000), MHCII (AbD Serotec, MCA46R, 1:2000), p-STAT1 (Tyr701, Cell Signaling Technology, #7649, 1:2000), p-STAT2 (Tyr690, Cell Signaling Technology, #4441, 1:2000), p-STAT3 (Tyr705, Cell Signaling Technology, #9145, 1:2000), STAT3 (Cell Signaling Technology, #9132, 1:2000), p-STAT5 (Tyr694, Cell Signaling Technology, #4322, 1:2000), p-STAT6 (Tyr641, Cell Signaling Technology, #9361, 1:2000), p-PI3K (p85;Tyr458/p55;Tyr199, Cell Signaling Technology, #4228, 1:2000), PI3K (p85, Cell Signaling Technology, #4292, 1:2000), p-Akt (Ser473, Cell Signaling Technology, #9271, 1:2000), Akt (Cell Signaling Technology, #9272, 1:2000), p-mTOR (Ser2448, Cell Signaling Technology, #2971 1:2000), mTOR (Cell Signaling Technology, #2972, 1:2000) and α-tubulin (Sigma-Aldrich, T-5168, 1:2000). Peroxidase activity on the membrane was visualized using a Davinch ChemiTM CAS-400 imaging system (Davinch K).

### Inhibitors

Pretreatment with an NK-1R antagonist (RP67580, 1 μM) and chemical inhibitors, a PI3K inhibitor (LY294002, 10 μM), an mTOR inhibitor (Rapamycin, 100 nM), an S6K1 inhibitor (PF4708673, 10 μM), and a MEK1/2 inhibitor (U0126, 10 μM) was performed before SP treatment. Functional blocking antibodies against IL-10 (1 μg ml^−1^) and IL-6 (0.2 μg ml^−1^) were also added before starting the incubation with SP.

### Isolation of PBMCs

8 ml of rat blood was collected from the abdominal aorta. The blood was diluted in 8 ml of PBS followed by 16 ml of Ficoll, and then centrifuged at 2200 rpm for 25 min at 4 °C. Subsequently, blood mononuclear cells (lymphocytes, monocytes, and thrombocytes among others) were collected. The cells were cultured in RPMI1640 complete medium.

### Proliferation ability and BrdU incorporation

Assessment of cell proliferation was performed by BrdU incorporation. The cells were treated with 20 mM BrdU in complete medium for 18 h and fixed in 3.7% formaldehyde after 3 d of culture with SP or cytokines. The cells were stained with BrdU antibody (BMC9318, Roche life science, #11-170-376-001, 1:50) and goat anti-mouse Alexa 488 secondary antibody. Each experimental condition was performed independently in triplicate. The BrdU-positive cells was counted (5 random fields/coverslip) at 100 × magnification (0.85 mm^2^) using the Adobe Photoshop CS6 program.

### Phagocytosis of *E. coli* and 25 μm microsphere

For *E. coli* phagocytosis, cells activated with various cytokines or SP for 3 d were incubated with fluorescein-conjugated *E. coli* BioParticles (25 μg ml^−1^, E-2861, Molecular Probes, Thermo Fisher Scientific Inc.) for 30 min at 37 °C. For 25 μm-microsphere phagocytosis, Fluoresbrite YG FITC microspheres (Polysciencesm Inc. #18241-2) were added at the beginning of the culture with SP or cytokines and incubated for 3 d. Cells were washed to remove non-specific attachment of *E. coli* or 25 μm microsphere then fixed in 3.7% formaldehyde. Actin staining was performed with TRITC-conjugated phalloidin (Sigma-Aldrich, 1:1000) for 30 min at RT to identify each engulfed cell. To evaluate the phagocytic efficiency, engulfing cells were counted. Each experimental condition was performed independently in triplicate. 10 random fields/coverslip at 200 × magnification (field = 2.74 mm^2^) under a Leica CTR 4000 fluorescence microscope were used for the quantitative analysis with the Adobe Photoshop CS6 program.

### Adhesion to HUVECs

Macrophages activated with SP or cytokines for 1 d were collected for PKH26 Red labeling (Sigma, MINI26), and 6 × 10^4^ cells were used for the HUVEC adhesion assay. HUVECs were prepared at a density of 3 × 10^4^ on transwell membrane at 1 d before addition of macrophages. After incubation with macrophages for 1 h at 37 °C, the transwell was fixed in 3.7% formaldehyde and subjected to immunostaining with VE-cadherin antibody (Cell Signaling Technology, #2500, 1:200). Each condition was performed in duplicate, and the HUVECs and macrophages were counted in 10 figures at 100 × magnification (0.85 mm^2^) using the Adobe Photoshop CS6 program.

### Cytokine array

Cytokine array (Raybio C-Series, Rat cytokine antibody array C2) was performed according to the manufacturer’s instructions. 1 ml of the 1-d cultured soup was added and incubated overnight at 4 °C. The membranes were washed and incubated with 1 ml of the prepared biotin-conjugated detection antibody cocktail for 2 h at RT, followed by a 30-min incubation with 2 ml HRP-conjugated streptavidin at RT. The membrane were developed using enhanced chemiluminescence-type solution and exposed to X-ray film. The array density was quantified using ImageJ software.

### ELISA

TGF-β1 ELISA (Quantikine ELISA, Mouse/RAT/Porcine/Canine TGF-β1 Immunoassay, #MB100B) was performed according to the manufacturer’s instructions. The supernatant was collected after culturing with SP or cytokines for 1 d. 50 μl of the activated samples was incubated in the TGF-β1 antibody-coated plate for 2 h. After washing, 100 μl of TGF-β1 conjugate was added for 2 h. 100 μl of the substrate solution was added for 30 min. Subsequently, 100 μl of stop solution was added, and the optical density was detected at 450 nm. Each experimental condition was performed independently in triplicate.

### MACS and FACS analysis

Cells from bone marrow, blood and spinal cord were collected and washed with MACS buffer (Miltenyi Biotec). The cells were incubated with CD14 (R&D Systems, MAB3822, 1:200) or CD11b (AbD serotec, MCA275R, 1:200) antibody for 15 min on ice. After washing, the cell suspension in 80 µl buffer was incubated with 20 µl of rat anti-mouse MicroBeads (Miltenyi Biotec) for 15 min on ice, followed by washing. The magnetically labeled cells were collected by flushing the column. For positive selection of CD206^+^ cells by FACS analysis, the total eluent from the MACS analysis was incubated with mouse CD206 antibody (Abcam, ab64693, 1:100) for 15 min on ice. After washing, cells in 80 µl buffer were incubated with 20 µl of rat anti-rabbit MicroBeads (Miltenyi Biotec) for 10 min on ice. After washing, cells were collected from the column. Fourteen rats were used for the CD14 MACS analysis, 8 rats for the CD11b MACS analysis and 8 rats for the CD11b MACS/CD206 FACS analysis.

### Spinal cord injury by contusion model

Rats were anesthetized with a mixture of ketamine (80 mg/kg; YuHan Corp.) and Rompun (7.4 mg/kg; Bayer) via intraperitoneal injection. The dorsal aspect of the spinal column was cut at the T9-T10 level to expose the cord without disrupting the dura. After stabilizing the spine between T8 and T10, the exposed cord was subjected to contusion injury using a New York University weight-drop device in which a 10 g weight impact rod was dropped from a height of 25 mm to produce a moderately contused SCI model, as described previously^32, 33^. Manual bladder emptying was performed two times daily until reflex bladder emptying was established.

### Macrophage adoptive therapy

Bone marrow cells were flushed from the femur and allowed to adhere for 1 h at 37 °C to collect monocytes. The cells were cultured for 5 d in complete medium and GM-CSF 10 ng ml^−1^, followed by incubation for 1 d with 100 nM SP. These cells were stained using the PKH67 Green Fluorescent Cell Linker Mini Kit for General Cell Membrane Labeling (Sigma, MINI67). 5 × 10^5^ cells/500 μl PBS intravenously injected at 1 d post-SCI. SP positive control group were injected with SP (5 nmol/kg) immediately after SCI; the control group received PBS at the same time points. Thirty-six rats were sacrificed to evaluate macrophage trafficking in the injured spinal cord at 2 d post-SCI.

### Tissue preparation and histological analysis

The animals were anesthetized and perfused with PBS and 3.7% formaldehyde for blood removal and fixation. The collected spinal cord was placed in 3.7% formaldehyde for 5 h and stored in 30% sucrose for 2 d. A 15-mm section of the spinal cord, centered at the injury site, was embedded in OCT compound (Sakura), and longitudinal sections were cut at 10 or 20 µm using a Leica Microtome for cryosectioning. Tissue sections were stained with H&E or immunofluorescence stained. The results were observed under a Leica CTR 4000 fluorescence microscope or a Zeiss LSM 510 META confocal microscope (Carl Zeiss). Every 2 stained tissue samples per rat were chosen at distances of 0.7 mm and 1.0 mm from the dorsal section, and the lesion cavity was analyzed with ImageJ software. Twenty-one rats were sacrificed for histological H&E staining and immunofluorescent staining at 14 d post-SCI.

### Basso, Besattie and Bresnahan (BBB) locomotor rating scale

Behavioral assessments were performed using the BBB locomotor rating scale, a 21-point scale (0–21) based on observations of the hind-limb movements. The BBB score was evaluated every week after injury. During the evaluation, the animals were allowed to walk freely on the open-field surface for 5 min while being observed by three blinded persons. Twenty-three rats were sacrificed for the BBB test at 6 w post-SCI.

### Statistical analysis

All data are presented as the mean ± SEM values. Statistical analyses were conducted using Prism (GraphPad) software. The unpaired student’s t-test and one-way ANOVA were used to determine statistically significant differences between groups (*P-value < 0.05).

### Data availability

The authors declare that all data supporting the findings of this study are available within the article and its Supplementary Information files, or are available from the corresponding author upon request.

## Electronic supplementary material

supplementary information
