# Supplementary material for: A neuropeptide, Substance-P, directly induces tissue-repairing M2 like macrophages by activating the PI3K/Akt/mTOR pathway even in the presence of IFNγ
Source: Sci Rep. 2017 Aug 25;7:9417. doi: 10.1038/s41598-017-09639-7 (PMC5573373; doi:10.1038/s41598-017-09639-7)

A neuropeptide, Substance-P, directly induces tissue-repairing M2 like macrophages by activating the PI3K/Akt/mTOR pathway even in the presence of IFNγ

Ji Eun Lim[1], Eunkyung Chung[1]* and Youngsook Son[1, 2]*

[1]Department of Genetic Engineering, College of Life Science and Graduate School of Biotechnology, Kyung Hee University, Yong In, 17104, [2]Kyung Hee Institute of Regenerative Medicine, Kyung Hee University Hospital, Seoul, Republic of Korea

Corresponding authors: Prof. Youngsook Son and Dr. Eunkyung Chung

Department of Genetic Engineering, College of Life Science and Graduate School of Biotechnology, Kyung Hee University, Yong In, 17104, Republic of Korea

E-mail: ysson@khu.ac.kr, chungek@hanmail.net or jieunlim8575@gmail.com

Tel: +82-31-201-3822; Fax: +82-31-206-3829

Supplementary Table

Table 1. M2^{SP} classification based on typical M2 subtypes and M1 type macrophages

		M2^{SP}	M2a$^{IL-4/13}$	M2c^{IL-10}	M1$^{IFN\gamma}$	MΦ^{GM-CSF}
M2 phenotype	Arginase-1	O	O			
	CD163	O	O	O		
	CD206	O	O			
	MHC II		O			
M1 phenotype	iNOS				O	O
	CCR7				O	O
Proliferation		O	O	O	O	O
Formation of MGCs			O			
Phagocytosis	*E. coli*	O		O	O	N.D
	Dead cells	O	O	O	O	
	Large particles (25 μm)		O			
Adhesion to HUVECs		O		O		
Cytokines	CINC-2α				O	O
	IL-2				O	
	IL-4		O			
	IL-6	O	O		O	O
	MMP-8	O		O		
	MCP-1	O	O	O		O
	IL-10	O	O	O		O
	VEGF	O	O	O		
	Prolactin R	O	O	O		O
State		Tissue repairing	Alternative	Deactivated	Classical, inflammatory	Proliferating precursor

Supplementary Figure legend

Supplementary Figure 1. Characterization of Mo^{BM} and MΦ$^{GM\text{-}CSF}$.

(**a**) Immunofluorescence staining of Mo^{BM} and MΦ$^{GM\text{-}CSF}$ for the monocyte marker CD14, the monocyte macrophage marker CD11b, the activated macrophage marker CD68, the M1 marker CCR7, the M2 markers CD206 and CD163, and the APC marker MHC class II. NK-1R expression was detected in both Mo^{BM} and MΦ$^{GM\text{-}CSF}$, and actin staining was used to observe cell morphology (n=3). (**b**) The absence of CD45 and CD29 expression in both Mo^{BM} and MΦ$^{GM\text{-}CSF}$ indicates the lack of lymphocytes and bone marrow stromal cells, respectively, in MΦ$^{GM\text{-}CSF}$ (n=3). (**c**) TRAP and ALP staining was detected to identify OCs and OBs, respectively. OCs/OBs were not observed in MΦ$^{GM\text{-}CSF}$. As a positive control, bone marrow mononuclear cells were treated with 60 ng ml^{-1} M-CSF and 100 ng ml^{-1} RANKL for 3 d to induce OC differentiation (Pink=TRAP, Purple=ALP) (n=2). Scale bar = 100 μm (**a,b**), 10 μm (high-magnification inset images in **a**) and 50 μm (**c**).

Supplementary Figure 2. SP acts as a mitogen in macrophages.

(**a**) Fold change of BrdU incorporation in MΦ$^{GM\text{-}CSF}$. (**b-c**) Withdrawal of GM-CSF in MΦ$^{GM\text{-}CSF}$ reduced the cell number by half in 3 d. The number of DAPI-positive cells (5 random fields/coverslip) was counted at 100 × magnification (0.85 mm^2) and is represented as the fold change of DAPI-positive cells relative to the start of the incubation, cont (0 d) (n=3). Data represents mean ± SEM (*** $p < 0.001$, unpaired t test).

Supplementary Figure 3. IL-4/13 induced the formation of multinucleated giant cells.

(**a**) At 3 d of treatment, IL-4/13-induced MGCs were negative for ALP/TRAP staining for OCs/OB. The positive control is shown in Supplementary Figure S1c (Pink=TRAP, Green=nuclear) (n=2). (**b**) IL-4/13-induced MGCs were also maintained with an additional treatment of GM-CSF at 3 d (Pink=TRAP, Green=nuclear) (n=2). Scale bar = 50 μm (**a**), 10 μm (high-magnification inset images in **a**) and 100 μm (**b**).

Supplementary Figure 4. SP-treated macrophages exhibited an elongated morphology.

$M2^{SP}$ exhibited a more elongated morphology than $M2a^{IL\text{-}4/13}$, $M2c^{IL\text{-}10}$, $M1^{INF\gamma}$, and $M\Phi^{GM\text{-}CSF}$ at 3 d after treatment. Although addition of GM-CSF with IL-4/13, IL-10 or IFNγ induced round morphogenic transformation, co-treatment with SP elongated $M2a^{IL\text{-}4/13}$, $M2c^{IL\text{-}10}$, and $M1^{INF\gamma}$. (**a**) The cell length (μm) was analyzed (5 random fields/coverslip) at 100 × magnification (0.85 mm^2) by ImageJ (n=3). (**b**) Representative images (Green=CCR7, Red=CD163, Blue=DAPI) (n=3). (**c**) The fold change of cell length from control. (Brackets)*; Fold change of the cell length from treatment of IL-4/13, IL-10, and IFNγ, respectively. Data represents mean ± SEM (*$p < 0.05$, ** $p < 0.01$, *** $p < 0.001$, unpaired t test). Scale bar = 20 μm (**b**).

Supplementary Figure 5. $M2^{SP}$ reveal highly dead cell debris phagocytic activity to dead cells and debris.

(**a-b**) The phagocytic activities toward dead blood and brain cells was assessed after 24 h. Excluding MΦ$^{GM\text{-}CSF}$, all the macrophage types showed high scavenging activities (Green=dead blood cell particles, Red=dead brain cell particles, Blue=DAPI). The number of positive cells was counted (5 random fields/coverslip) at 100 × magnification (0.85 mm^2) (n=2). Data represent the mean ± SEM (** $p < 0.01$, *** $p < 0.001$, unpaired t test). Scale bar = 10 μm (high-magnification image inset in **a**).

Supplementary Figure 6. IL-4/13-induced MGCs engulfed the large microspheres

(**a**) Enlarged images of Figure 5c were presented (Green=25 μm Fluoresbrite microspheres, Red=Actin, Blue=DAPI). (**b**) Another experiment set was presented. 25 μm microspheres were inside of the cells (white arrows) (Green=25 μm Fluoresbrite microspheres, Red=Actin, Blue=Topro). Scale bar = 100 μm (**a,b**) 50 μm (high-magnification image inset in **a,b**).

Supplementary Figure 7. M2^{SP} reveals endothelial adhesive activity.

CD68 staining were performed to identify the macrophages. "Bottom" image confirms the VE-cadherin boundary of HUVECs and "Top" image confirms the attachment of macrophages. 3 D integrated image was acquired from Z stack microscopic images. Enlarged images shows the presence of $CD68^+$ macrophages. Black arrows represent the attached macrophages on HUVECs (Green=VE-cadherin, Red= CD68, Blue=DAPI). Scale bar = 100 μm.

Supplementary Figure 8. SP can activate bone marrow-derived monocytes to express an M2 phenotype.

(**a**) RT-PCR analysis showed that SP and IL-4/13 both increased the mRNA levels of M2 polarization markers, including Arginase-1, IL-10, PPAR-γ, CD206, CD163, VEGF, and TGF-β, as well as the M1 polarization markers iNOS and TNF-α at 6 h of treatment. The mRNA levels of IL-6 and IL-1β were not significantly altered by SP or IL-4/13 treatment (n=2). (**b, c**) Mo^{BM} differentiated into $CD68^{+}CD206^{+}$ and $CD163^{+}$ macrophages after treatment with SP and IL-4/13 at 3 d, respectively. IL-10 did not induce the formation of $CD68^{+}CD206^{+}CD163^{+}$ macrophages (**b**) SP-primed monocytes had an elongated morphology (Green=CD68, Red=CD206, Blue=DAPI) (n=3). (**c**) (Green=CD163, Blue=DAPI) (n=3). Scale bar = 100 μm (**b,c**) and 10 μm (high-magnification inset images in **c**).

Supplementary Figure 9. SP induces Arginase-1 expression in THP-1 monocytes and THP-1-derived macrophages.

(**a**) Arginase-1 expression in THP-1 monocytes was increased by SP in a dose-dependent manner at 6 h, and this induction was blocked by RP67580. The western blot results of Arginase-1/α-tubulin were detected in the same blot (n=2). (**b**) THP-1 monocytes were differentiated into M1-like macrophages after 1 d of PMA treatment. SP induced the expression of Arginase-1 in a dose-dependent manner at 6 h in these cells, and this expression was blocked by RP67580. The western blot results of Arginase-1/α-tubulin were detected in the same blot. (n=2).

Supplementary Figure 10. SP induces PI3K/Akt/mTOR activation in THP-1 monocytes.

(**a**) SP activated PI3K, Akt, and mTOR in THP-1 monocytes within 30 min. Vertically sliced images were selected from the (**b-d**) images to show the phosphorylation of specific protein clearly. The samples derived from the same experiment. (**b-d**) The western blot results of P-PI3k/PI3K, P-Akt/Akt, and P-mTOR/mTOR were detected in the same blot, separately. (n=2).

Supplementary Figure 11. SP induces PI3K/Akt/mTOR activation in THP-1-derived macrophages.

(**a**) SP activated PI3K, Akt, and mTOR in THP-1-derived macrophages within 30 min. Vertical sliced images were selected from the (**b-d**) images to show the phosphorylation of specific protein clearly. The samples derived from the same experiment. (**b-d**) The western blot results of P-PI3k/PI3K, P-Akt/Akt, and P-mTOR/mTOR were detected in the same blot, separately. (n=2).

Supplementary Figure 12. $M2^{SP}$ adoptive transfer increased $MAP2^{+}$ neuronal survival.

At 2 d post-SCI, immunofluorescence staining of $MAP2^{+}$ neurons and MBP^{+} oligodendrocytes were performed. Longitudinal tissue sections were chosen at a distance of ~ 800 μm from the dorsal surface of the injury site. Full spinal cord section and enlarged images were shown. Image #1 is the normal part and image #2-3 are the lesion part. $M2^{SP}$ adoptive transfer enhanced $MAP2^{+}$ neuronal survival in the lesion site similarly to that of SP injected group. White arrows present the $MAP2^{+}$ neurons (Green=MAP2, Red=MBP, Blue=DAPI) (n=3). Scale bar = 100 μm.

Supplementary Figure 13. M2^{SP} adoptive transfer increased NeuN^{+} TUNEL^{-} neuronal survival.

(**a**) At 2d post-SCI, TUNEL assay and NeuN immunofluorescent staining were performed. Longitudinal tissue sections were chosen at a distance of ~ 800 μm from the dorsal surface of the injury site. M2^{SP} adoptive transfer enhanced NeuN^{+} TUNEL^{-} survived neuronal cells in the lesion site similar to those of SP injected group. White arrows indicate the NeuN^{+} TUNEL^{-} neurons (Green=TUNEL, Red=NeuN, Blue=DAPI) (n=3). (**b**) Immunofluorescent staining of CD68^{+} macrophage/microglia was performed. CD68^{+} macrophage/microglia were not co-localized with TUNEL. (Green=TUNEL, Red=CD68, Blue=DAPI) (n=3). (**c**) Immunofluorescence staining of GFAP^{+} astrocyte was performed. GFAP^{+} astrocytes were not co-localized with TUNEL. (Green=TUNEL, Red=CD68, Blue=DAPI) (n=3). Scale bar = 200 μm.

Supplementary Figure 14. M2^{SP} adoptive transfer increases CD68^{+}CD206^{+} cells in the injured spinal cord.

At 14 d post-SCI, CD68^{+}CD206^{+} cells were still present. Longitudinal tissue sections were chosen at a distance of ~ 700 μm from the dorsal surface of the injury site. (n=3) (Green=CD68, Red=CD206, Blue=DAPI) (n=3). Scale bar = 100 μm.

Supplementary Figure 15. Histology analysis of the wound cavity

Histology analysis of the lesion cavity delineated by H&E stain at 14 d post-SCI. (PBS group, n=8; MΦ, n=8; $M2^{SP}$, n=8; SP, n=3).

Supplementary Figure 16. SP induces M2 polarization through early induction of Arginase-1.

(**a**) The increased expression of Arginase-1 was detected after 6 h of SP treatment in $MΦ^{GM-CSF}$. Full-length blots of Figure 1e are presented. The western blot results of Arginase-1/α-tubulin were detected in the same blot. (**b**) Dose-dependent increased activity and expression of Arginase-1 determined after 6 h of SP treatment. This was completely blocked by pretreatment with the NK-1R antagonist RP67580 (1 μM). Full-length blots of Figure 1g are presented. The western blot results of Arginase-1/α-tubulin were detected in the same blot.

Supplementary Figure 17. Identification of SP-induced macrophages as a distinct M2 subtype compared with IL-4/13- and IL-10-induced macrophages.

(**a-d**) After 3 d of SP treatment, CD68, CD206, and MHCII were examined by western blot analysis. Full-length blots of Figure 2e are presented. The samples derived from the same experiment. (**a**) CD206. (**b**) CD68. (**c**) MHCII. (**d**) α-tubulin. (**a,d**) The western blot results of CD206/α-tubulin were detected from one blot.

Supplementary Figure 18. Identification of SP-induced macrophages as a distinct M2 subtype compared with IL-4/13- and IL-10-induced macrophages.

(**a-d**) Neither STAT3 nor STAT6 was activated by SP treatment at 6 h, which are known to involved in the IL-10 and IL-4/13-induced signaling pathway, respectively. Full-length blots of Figure 2g are presented. The samples derived from the same experiment. (**a-c**) The western blot results of P-STAT3, STAT3, and P-STAT6 were detected in the same blot. (**d**) The western blot results of α-tubulin were detected in the same blot.

Supplementary Figure 19. SP activates the NK-1R/PI3K/Akt/mTOR signaling pathway.

(**a-b**) Western blot analysis showed that SP-activated PI3K and Akt were detected at 5 min and 20 min. The blockade of NK-1R by pretreatment with RP67580 led to inhibition of PI3K and Akt. Full-length blots of Figure 3a are presented. The samples derived from the same experiment. (**a**) The western blot results of P-PI3K/PI3K were detected in the same blot. (**b**) The western blot results of P-Akt/Akt were detected in the same blot.

Supplementary Figure 20. SP activates the NK-1R/PI3K/Akt/mTOR signaling pathway.

(**a-b**) Western blot analysis showed that SP-activated mTOR were detected at 5 min and 20 min. The blockade of NK-1R by pretreatment with RP67580 led to inhibition of mTOR. Full-length blots of Figure 3a are presented. The samples derived from the same experiment. (**a**) The western blot results of P-mTOR/mTOR were detected in the same blot. (**b**) The western blot results of α-tubulin were detected in the same blot.

Supplementary Figure 21. SP activates the NK-1R/PI3K/Akt/mTOR/S6K signaling

pathway, leading to the induction of Arginase-1.

(**a-b**) All inhibitors, such as the NK-1R antagonist RP67580, PI3K inhibitor LY294002, mTOR inhibitor rapamycin, and S6K inhibitor PF4708671, completely blocked the elevated SP-induced Arginase-1 expression at 6 h. Full-length blots of Figure 3f are presented. The western blot results of Arginase-1/α-tubulin were detected in the same blot.

Supplementary Figure 22. SP induces the Arginase-1 and CD163 expression through the NK-1R signaling in PBMCs.

(**a-b**) Rat PBMCs also showed increased expression of Arginase-1 and CD163 at 6 h and 1 d after SP treatment, respectively. Pretreatment with RP67580 blocked the increased SP-induced expression of Arginase-1 and CD163, as demonstrated by western blot analysis. Full-length blots of Figure 3h are presented. (**a**) The western blot results of Arginase-1/α-tubulin were detected in the same blot. (**b-c**) The western blot results of Arginase-1/α-tubulin were detected from one blot. (**c-d**) The western blot results of α-tubulin were detected in the same sample.

Supplementary Figure 23. Identification of SP-induced macrophages as a distinct M2 subtype compared with IL-4/13-, IL-10- IFNγ- and GM-CSF- induced macrophages.

(**a-f**) SP did not activate STAT1, STAT3, STAT5, or STAT6, and SP co-treatment did not affect specific cytokine-mediated STAT expression such as STAT6 by IL-4/13, STAT3 by IL-10, STAT1 by IFNγ, and STAT5 by GM-CSF at 6 h. Full-length blots of Figure 4g are presented. The samples derived from the same experiment. (**a-b**) The western blot results of P-STAT6 and

P-STAT1 were detected in the same blot. (**c-d**) The western blot results of P-STAT3 and P-STAT5 were detected in the same blot. (**e-f**) The western blot results of P-STAT2 and α-tubulin were detected in the same blot.

Supplementary Figure 24. SP can activate bone marrow-derived monocytes to express an M2 phenotype.

(**a-e**) Western blot analysis showed that SP increased the expression of Arginase-1 and IL-10 but not the expression of iNOS or TNF-α in Mo^{BM} after 6 h of treatment. Full-length blots of Figure 6a are presented. The samples derived from the same experiment. (**a-b**) The western blot results of Arginase-1 and IL-10 were detected in the same blot. (**c**) iNOS. (**d-e**) The western blot results of TNF-α and α-tubulin were detected in the same blot

Supplementary Figure 25. SP can activate bone marrow-derived monocytes to express an M2 phenotype.

(**a**) SP and IL-4/13 increased the expression of CD206 and CD163 but not the expression of CD68 or CCR7 after 1 d of treatment. Only IL-4/13 Strongly increased the expression of MHCII. Full-length blots of Figure 6b are presented. The samples derived from the same experiment. (**a-b**) The western blot results of CD206 and CD163 were detected in the same blot. (**c**) CD68. (**d**) CCR7. (e) MHCII. (f) α-tubulin.

Supplementary data methods

Cell immunofluorescent staining

Bone marrow-derived mononuclear cells obtained from rats were prepared for characterization of Mo^{BM} and $M\Phi^{GM\text{-}CSF}$ by immunofluorescent staining. Cells that were prepared following the procedures described above were immediately fixed in 3.7% formaldehyde and blocked with 5% skim milk in PBS containing 0.2% Triton X-100 (Sigma-Aldrich). They were exposed to the following primary antibodies at 4 °C overnight: CD14 (R&D Systems, MAB3822, 1:200), CD11b (AbD Serotec, MCA275R, 1:200), CD68 (Millipore, MAB1435, 1:200), CCR7 (Abcam, ab32527, 1:1000), CD206 (Abcam, ab64693, 1:200), CD163 (AbD Serotec, MCA342R, 1:100), MHCII (AbD Serotec, MCA46R, 1:200), NK-1R (Novus Biologicals, NB300-101, 1:50), CD45 (MEM56, Millipore, #05-1413, 1:200), and CD29 (Chemicon, MAB1981, 1:200). The secondary antibodies were goat anti-rabbit Alexa 488 (Jackson ImmunoResearch, 1:500), goat anti-mouse Alexa 488 (Jackson ImmunoResearch, 1:500), goat anti-rabbit Cy3 (Jackson ImmunoResearch, 1:500), and goat anti-mouse Cy3 (Jackson ImmunoResearch, 1:500). The cells were mounted with Vectashield mounting medium containing DAPI (Vector Laboratories), and the results were observed under a Leica CTR 4000 fluorescence microscope (Leica Microsystems) or a Zeiss LSM 510 META confocal microscope (Carl Zeiss). Each experimental condition was performed independently in triplicate. The number of positive cells was counted (5 random fields/coverslip) at 100 × magnification (0.85 mm^2) using the Adobe Photoshop CS6 program.

TRAP and ALP double staining

Tartrate-resistant acid phosphatase (TRAP) and alkaline phosphatase (ALP) are enzyme markers of osteoclasts and osteoblasts, respectively. As a positive control for osteoclasts and osteoblasts, bone marrow mononuclear cells were treated with M-CSF 60 ng ml^{-1} and RANKL 100 ng ml^{-1} for 3 d. Osteoblast and osteoclast double staining (TRAP & ALP double-stain kit, TaKaRa, # MK300) was performed according to the manufacturer's protocol. The cells were fixed in 60% acetone and 10% methanol and incubated with TRAP substrate solution for 30 min at 37 °C, and the solution was then removed. Another substrate solution for ALP was added, followed by incubation for 30 min at 37 °C and three washes with sterilize distilled water. Finally, the cell nuclei were stained with methyl green for 10 min. Each experimental condition was performed independently in duplicate.

Phagocytosis of dead cells

To assess the ability to remove dead cells and cell debris, cells activated with various cytokines and SP were incubated with PKH-labeled cells isolated from PBMCs and brain tissue. A total of 4×10^5 cells from PBMCs and brain were labeled with PKH67 Green (Sigma, MINI67) and PKH26 Red (Sigma, MINI26) according to the manufacturers' instructions. Dead cells were prepared as previously reported[49]. Incubation with 100 μM and 500 μM H_2O_2 for 4 h was performed to induce apoptosis and necrosis, respectively. Combined apoptotic and necrotic dead cells were used for the phagocytosis assay. $M\Phi^{GM\text{-}CSF}$ activated with SP and various cytokines were incubated with labeled blood and brain cells for 24 h, followed by fixation in 3.7% formaldehyde. Each experimental condition was performed independently in duplicate.

The number of positive cells was counted (5 random fields/coverslip) at 100 × magnification (0.85 mm^2) using the Adobe Photoshop CS6 program.

Proliferation ability

Cell proliferation was assessed by 5-bromodeoxyuridine (BrdU) incorporation into Mo^{BM} and $M\Phi^{GM\text{-}CSF}$ cultures. The numbers of DAPI-positive cells were counted (5 random fields/coverslip) at 100 × magnification (0.85 mm^2) using the Adobe Photoshop CS6 program. Each experimental condition was performed independently in triplicate.

RNA isolation and RT-PCR

RT-PCR was performed after isolation of RNA from cells using TRIzol reagent (Invitrogen). The cDNA was generated using a Random Primer DNA Labeling kit (Takara) according to the manufacturer's instructions and then used as a template for reverse-transcription PCR amplification using TAKARA Ex TaqTM (Takara Bio Inc.). The primers were synthesized (Cosmo Genetech) as follows: iNOS (forward primer, 5′-CTA CCT ACC TGG GGA ACA CCT GGG-3′; reverse primer, 5′-GGA GGA GCT GAT GGA GTA GTA GCG G-3′), Arginase-1 (forward primer, 5′-TGT GAA GAA CCC ACG GTC TG-3′; reverse primer, 5′- ATG GGC CTT TTC TTC CTT CCC-3′) CD206 (forward primer, 5′-AGT CTG CCT TAA CCT GGC AC-3′; reverse primer, 5′-AGG CAC ATC ACT TTC CGA GG-3′), CD163 (forward primer,

5′-TTA CTT GCT CAG GCC ACA GG-3′; reverse primer, 5′-CAG TGC AGT GGA ACT TGT GC-3′), PPARγ (forward primer, 5′-TCC CGT TCA CAA GAG CTG AC-3′; reverse primer, 5′-TGG CAC CCT TGA AAA ATG CG-3′), IL-10 (forward primer, 5′-GCT CAG CAC TGC TAT GTT GC-3′; reverse primer, 5′-AAT CGA TGA CAG CGT CGC A-3′), TNF-α (forward primer, 5′-GAA CTC AGC GAG GAC ACC AA-3′; reverse primer, 5′-TAG ACA ACT GGC TGC ATG GG-3′), TGF-β1 (forward primer, 5′-CTT TGT ACA ACA GCA CCC GC-3′; reverse primer, 5′-TCG ACG TTT GGG ACT GAT CC-3′), VEGF (forward primer, 5′-TGT ACA AGT GCC AGC TAA GGA A-3′; reverse primer, 5′-CAC ACG TAG TTT GCT GGA CAA G-3′), IL-1β (forward primer, 5′-CAC TTC ACA AGT CGG AGG CT-3′; reverse primer, 5′-AGC ACA CTA GGT TTG CCG AG-3′), IL-6 (forward primer, 5′-CAC TTC ACA AGT CGG AGG CT-3′; reverse primer, 5′-AGC ACA CTA GGT TTG CCG AG-3′), GAPDH (forward primer, 5′-GCT GAG AAT GGG AAG CTG GT-3′; reverse primer, 5′-TAA GCA GTT GGT GGT GCA GG-3′). Amplification was performed using a Bio-Rad C1000TM Thermal Cycler system (Bio-Rad) with an initial incubation at 95°C for 2 min, followed by 35 cycles at 98°C for 10 sec, 60°C (for IL-1β, IL-6, iNOS, PPARγ, TNF-α, CD206)/ 58.1°C (for VEGF)/ 61.7°C (for Arginase-1)/ 57.5°C (for CD163)/ 55.4°C (for IL-10)/ 59°C (for TGF-β1) for 30 sec, and 72°C for 1 min. Each experimental condition was performed independently in

duplicate.

THP-1 cell line culture

To examine the effect of SP on M2 polarization in human monocytes and macrophages, the human acute monocytic leukemia THP-1 cell line was used (Korean Cell Line Bank, #40202, human: male, 1-year old, negative Mycoplasma test, positive QC test). This cell line was obtained from the Korean Cell Line Bank via import from the American Type Culture Collection (ATCC). Frozen medium consisted of 52.5% RPMI1640, 40% FBS and 7.5% DMSO. KCLB medium consisted of 90% RPMI1640 with L-glutamine (300 mg/L), 25 mM HEPES, 25 mM NaHCO3 and 10% heat-inactivated FBS. Complete medium consisted of 90% RPMI1640 and 10% heat-inactivated FBS. The differentiation of THP-1 monocytes into macrophages (THP-1 MΦ) was conducted using a phorbol 12-myristate 13-acetate (PMA) concentration of 10 ng ml^{-1} for 24 h, followed by three washes with PBS and culturing in completed medium for another 48 h for deactivation. The cells were then treated with SP and RP67580. Each experimental condition was performed independently in duplicate.

Tissue immunofluorescent staining

The animals were anesthetized with a mixture of ketamine and Rompun, and perfused with PBS and 3.7% paraformaldehyde for blood removal and fixation. The spinal cord was collected and placed in 3.7% formaldehyde for 5 h. Then, the cord was transferred to 30% sucrose in

PBS and stored for 2 d. A 15-mm section of the spinal cord, centered at the injury site, was embedded in OCT compound (Sakura), and longitudinal sections were cut at 10 or 20 μm. Tissue was blocked with 5% skim milk in PBS containing 0.2% Triton X-100 (Sigma-Aldrich). They were exposed to the following primary antibodies at 4 °C overnight: MAP2 (Millipore, MAB3418, 1:200) and MBP (Santa Cruz BioTechnology, sc-13914, 1:200). The secondary antibodies were Rabbit anti-mouse Alexa 488 (Jackson ImmunoResearch, 1:500), rabbit anti-goat Cy3 (Jackson ImmunoResearch, 1:500). The tissues were mounted with Vectashield mounting medium containing DAPI (Vector Laboratories), and the results were observed under a Leica CTR 4000 fluorescence microscope (Leica Microsystems) or a Zeiss LSM 510 META confocal microscope (Carl Zeiss).

TUNEL assay

TUNEL assay was performed according to the manufacturer's protocol (In situ Cell Death Detection kit, Fluorescein, Roche, #11 684 795 910). Tissue was blocked with 5% skim milk in PBS containing 0.2% Triton X-100 (Sigma-Aldrich). They were exposed to the following primary antibodies at 4 °C overnight: NeuN (Millipore, MAB377, 1:300), GFAP (Abcam, ab7260, 1:500) or CD68 (abcam, ab125212, 1:200). After washing 3 times, tissue was fixed with fixation solution (aceton: ethanol=1:2). 50 μl TUNEL reaction mixture was treated for 1 h at RT. After washing 3 times, the secondary antibodies, Goat anti-mouse Cy3 (Jackson ImmunoResearch, 1:500) or Goat anti-rabbit Cy3 (Jackson ImmunoResearch, 1:500) was treated for 1 h and then mounted with Vectashield mounting medium containing DAPI (Vector Laboratories). The results were observed under a Zeiss LSM 510 META confocal microscope (Carl Zeiss).

Supplementary Figure 1

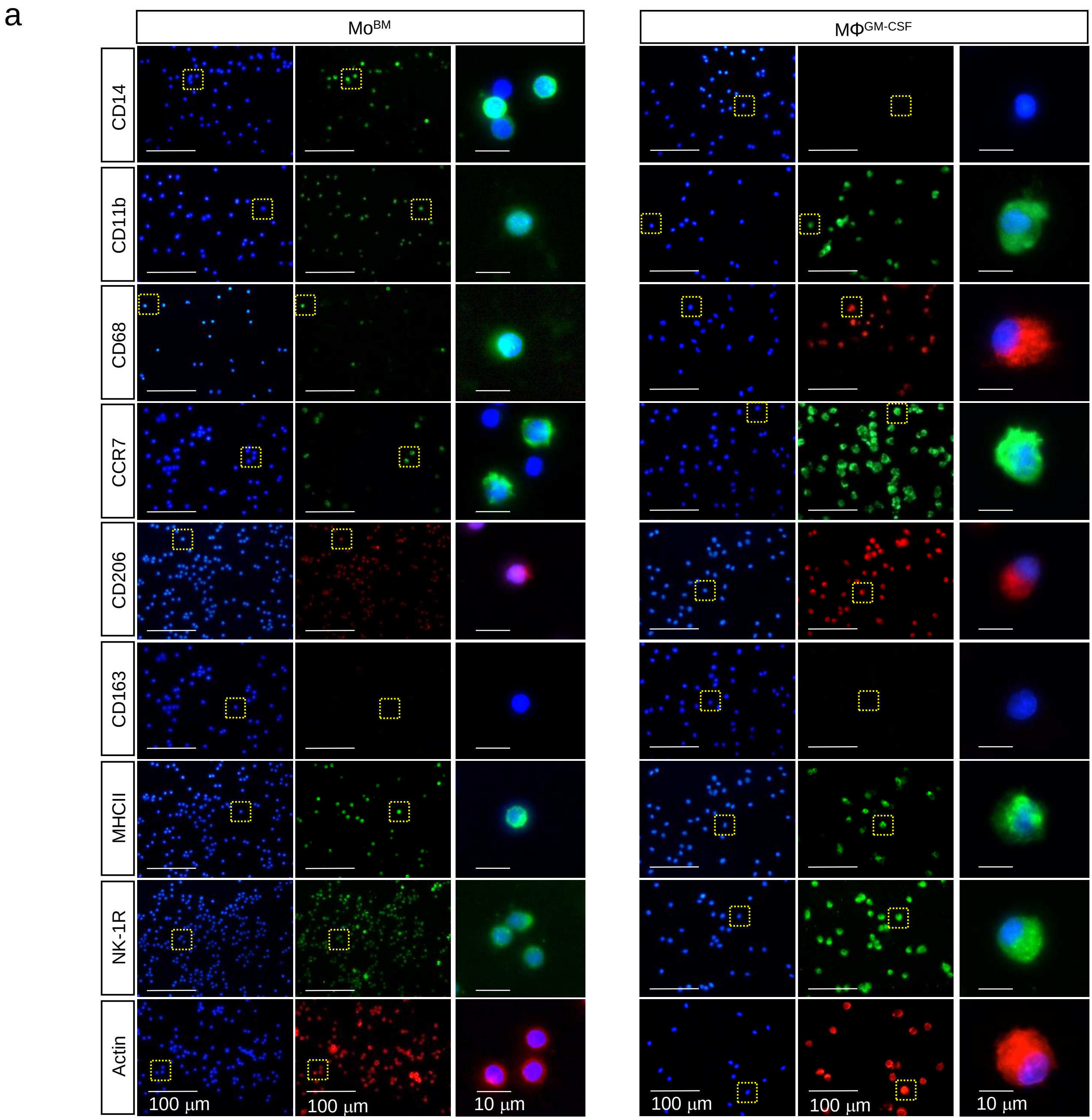

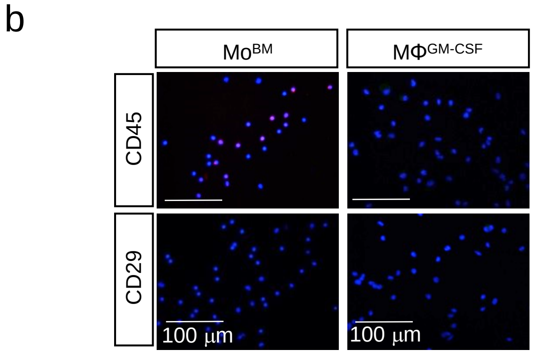

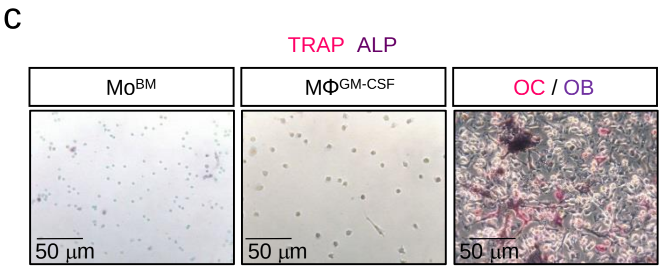

Supplementary Figure 2

a Fold change of BrdU incorporation in MΦ$^{GM\text{-}CSF}$

	Cont	SP	IL-4/13	IL-10	IFNγ	GM-CSF
	1	4.7	4.9	7.5	2.4	25.6
+ SP			20.3	5.9	10.9	24.4
+ GM-CSF			25.0	31.7	24.7	

b

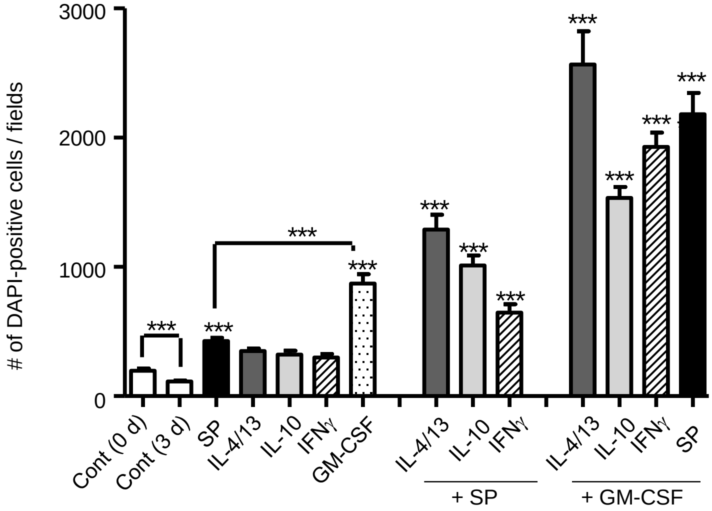

c Fold change of total cells in MΦ$^{GM\text{-}CSF}$

	Cont (0 d)	Cont (3 d)	SP	IL-4/13	IL-10	IFNγ	GM-CSF
	1	0.6	3.9	3.2	2.9	3.0	8.8
+ SP				12.0	9.6	5.8	20.2
+ GM-CSF				20.7	13.5	18.7	

Supplementary Figure 3

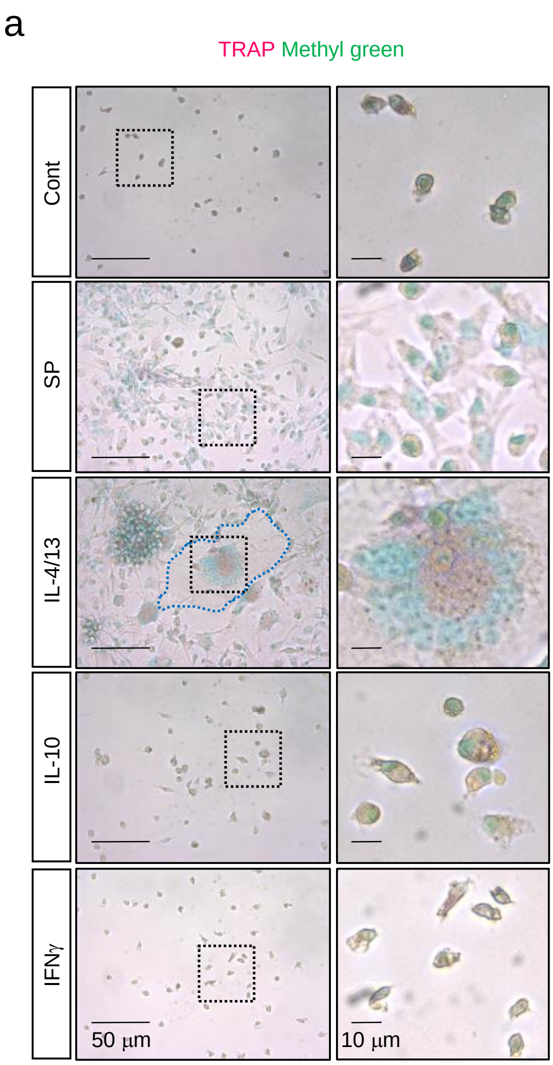

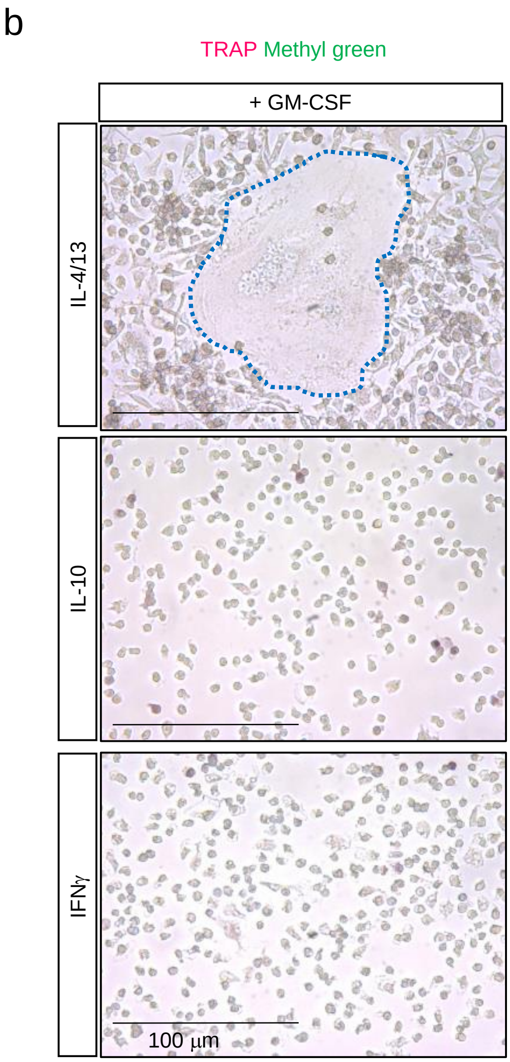

Supplementary Figure 4

a

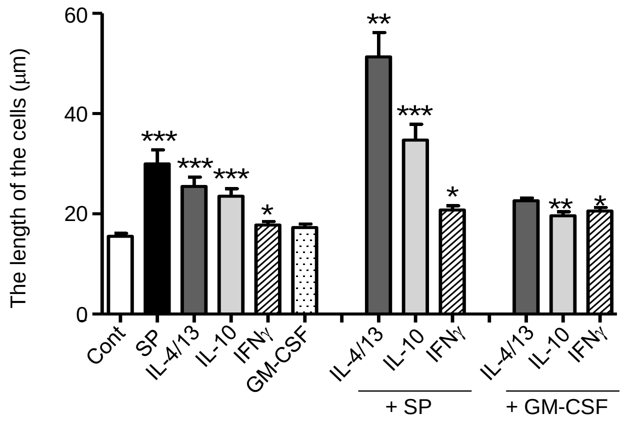

b

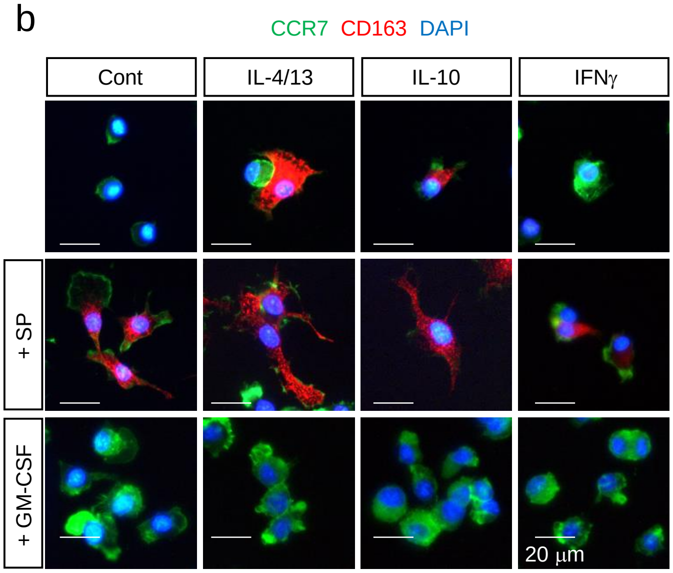

c

Fold change of the cell length

	Cont	SP	IL-4/13	IL-10	IFNγ	GM-CSF
	1	1.9	1.6 (1)*	1.5 (1)*	1.1 (1)*	1.1
+ SP			3.3 (2.0)*	2.2 (1.5)*	1.3 (1.2)*	
+ GM-CSF			1.5 (0.9)*	1.3 (0.8)*	1.3 (1.2)	

* Fold change of the cell length from treatment of IL-4/13, IL-10, and IFNγ, respectively

Supplementary Figure 5

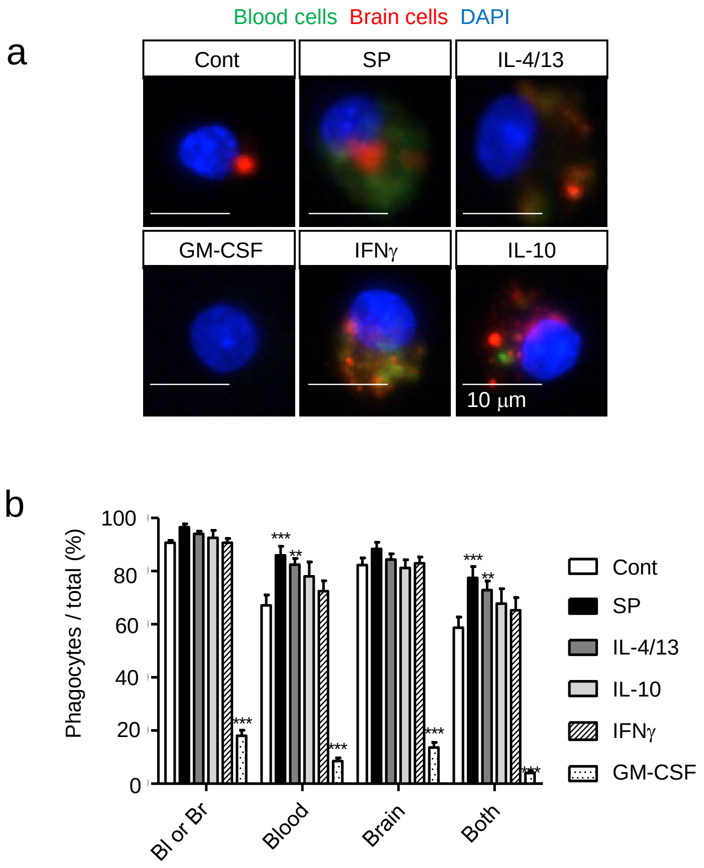

Supplementary Figure 6

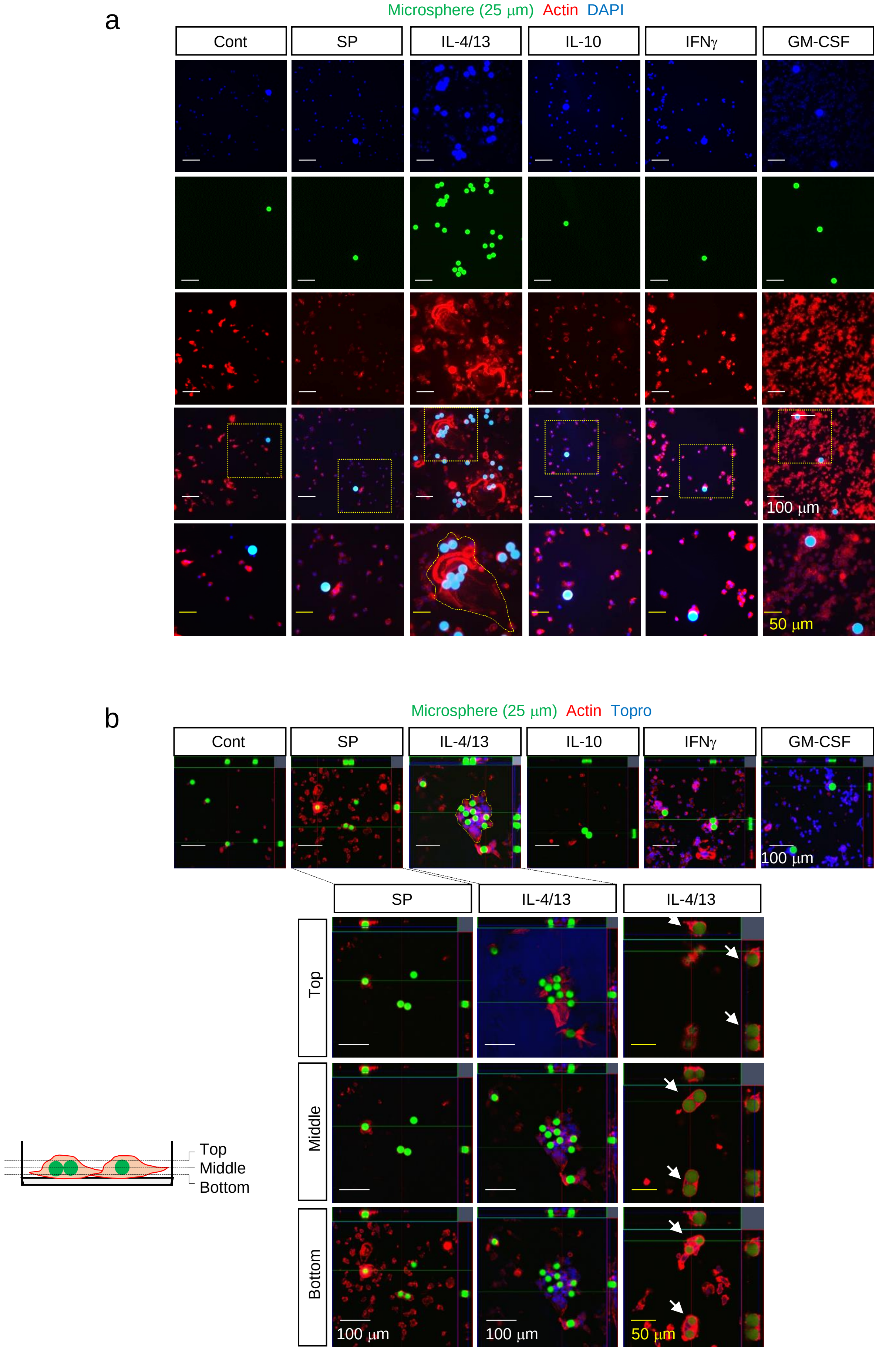

Supplementary Figure 7

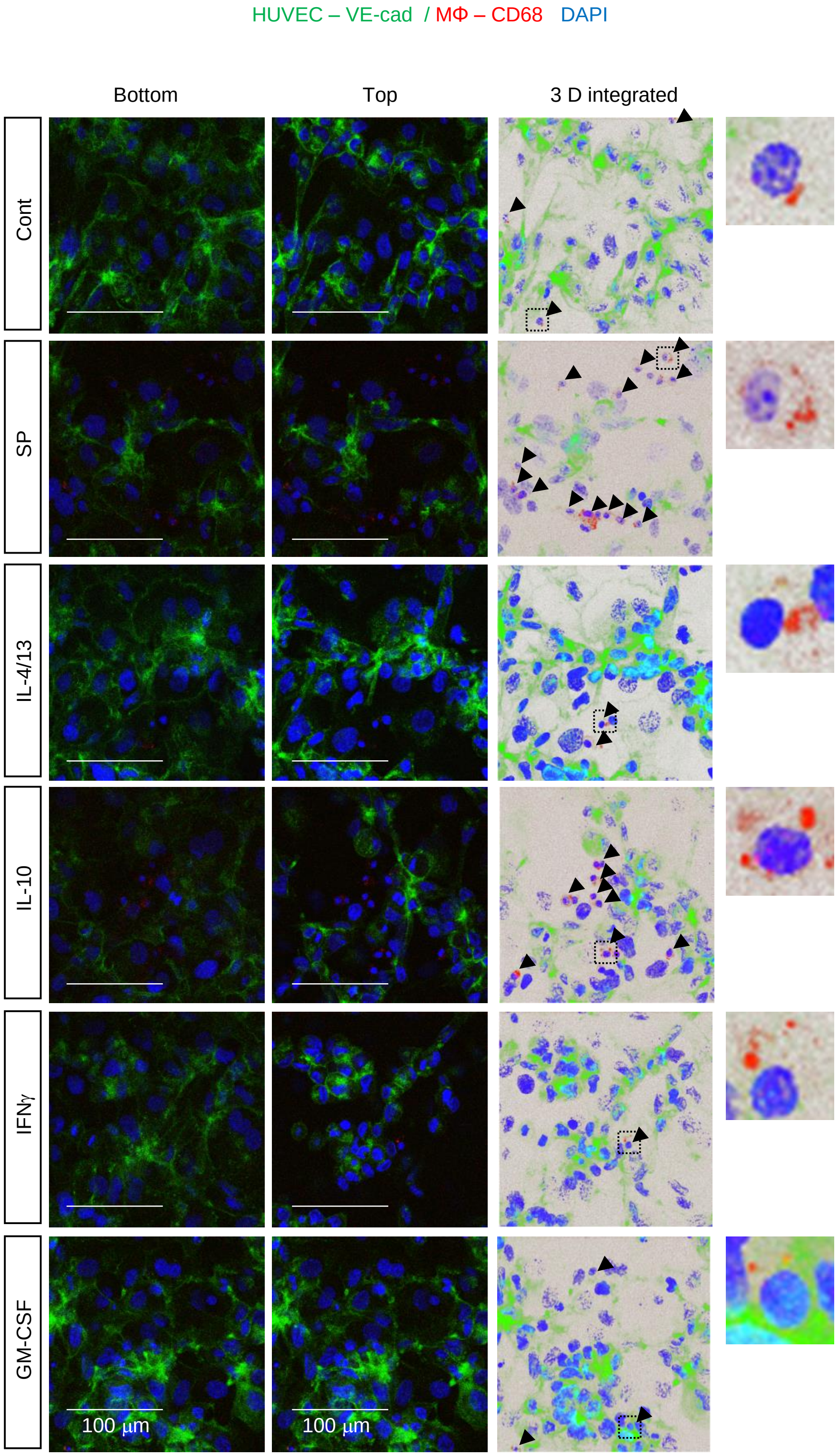

Supplementary Figure 8

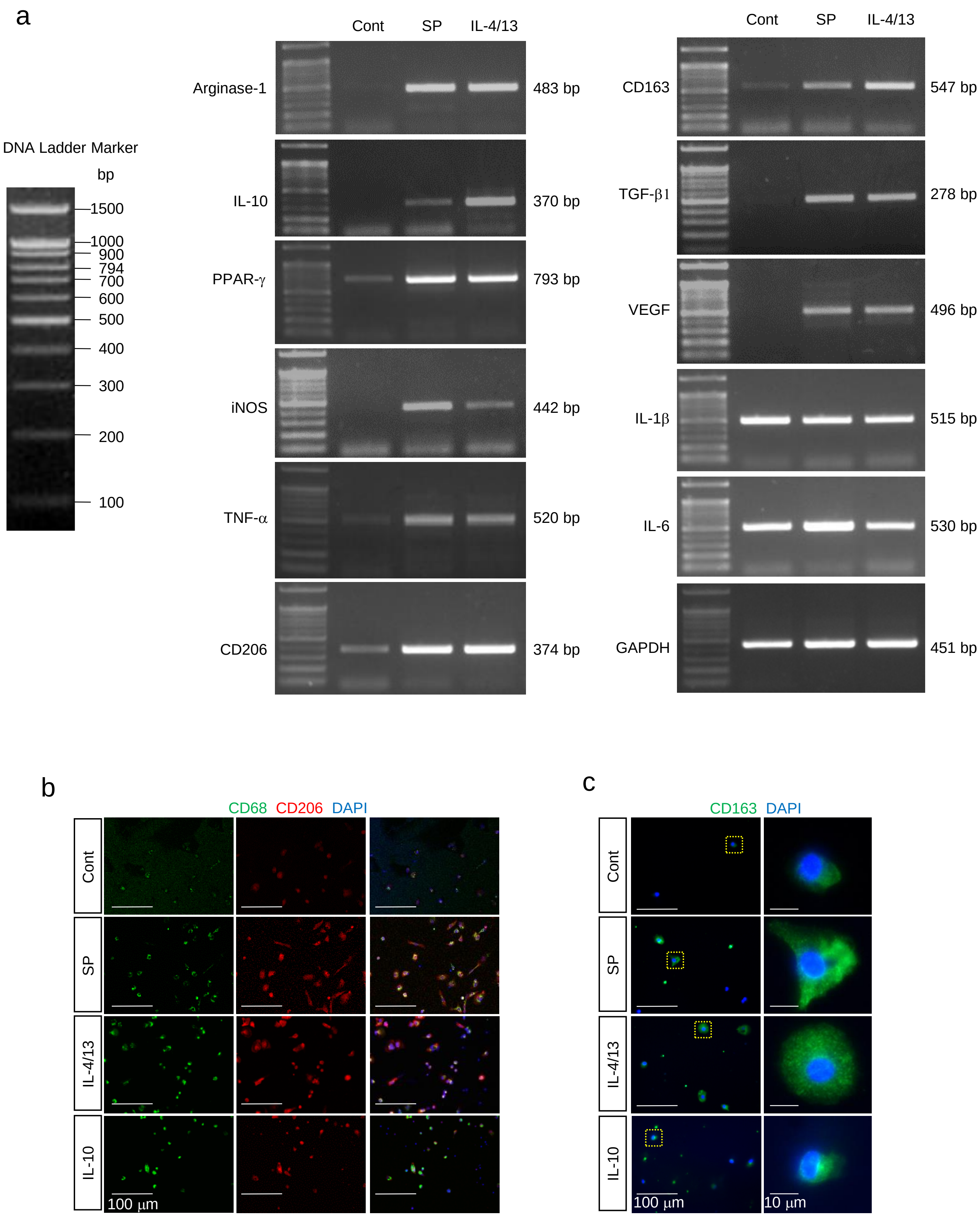

Supplementary Figure 9

a THP-1

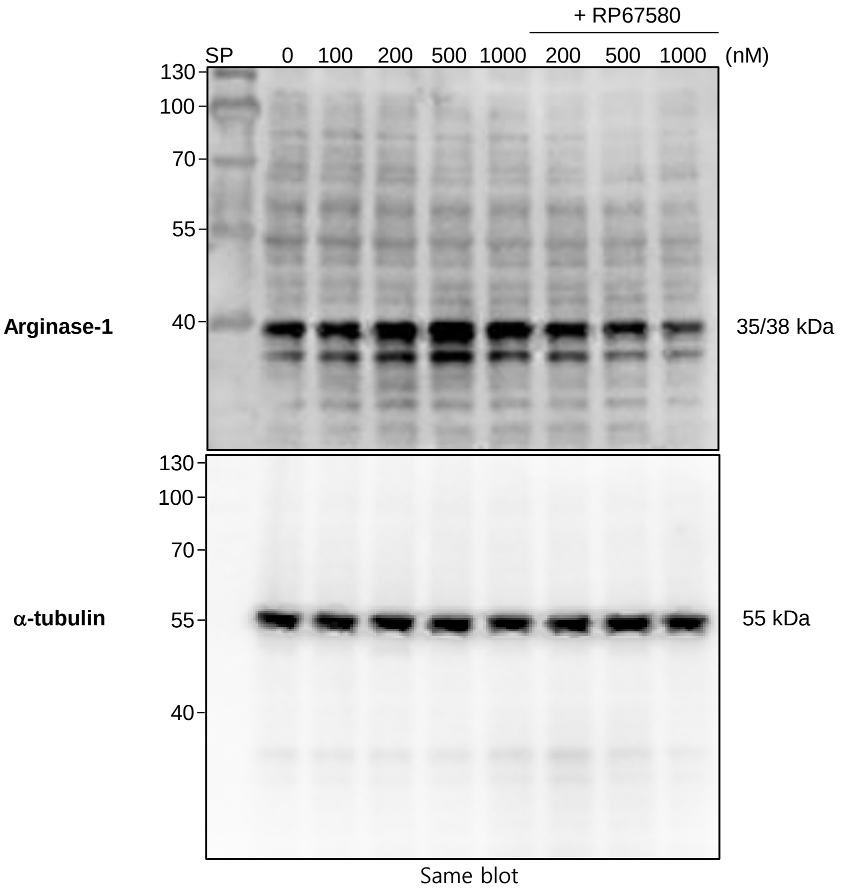

b THP-1 MΦ

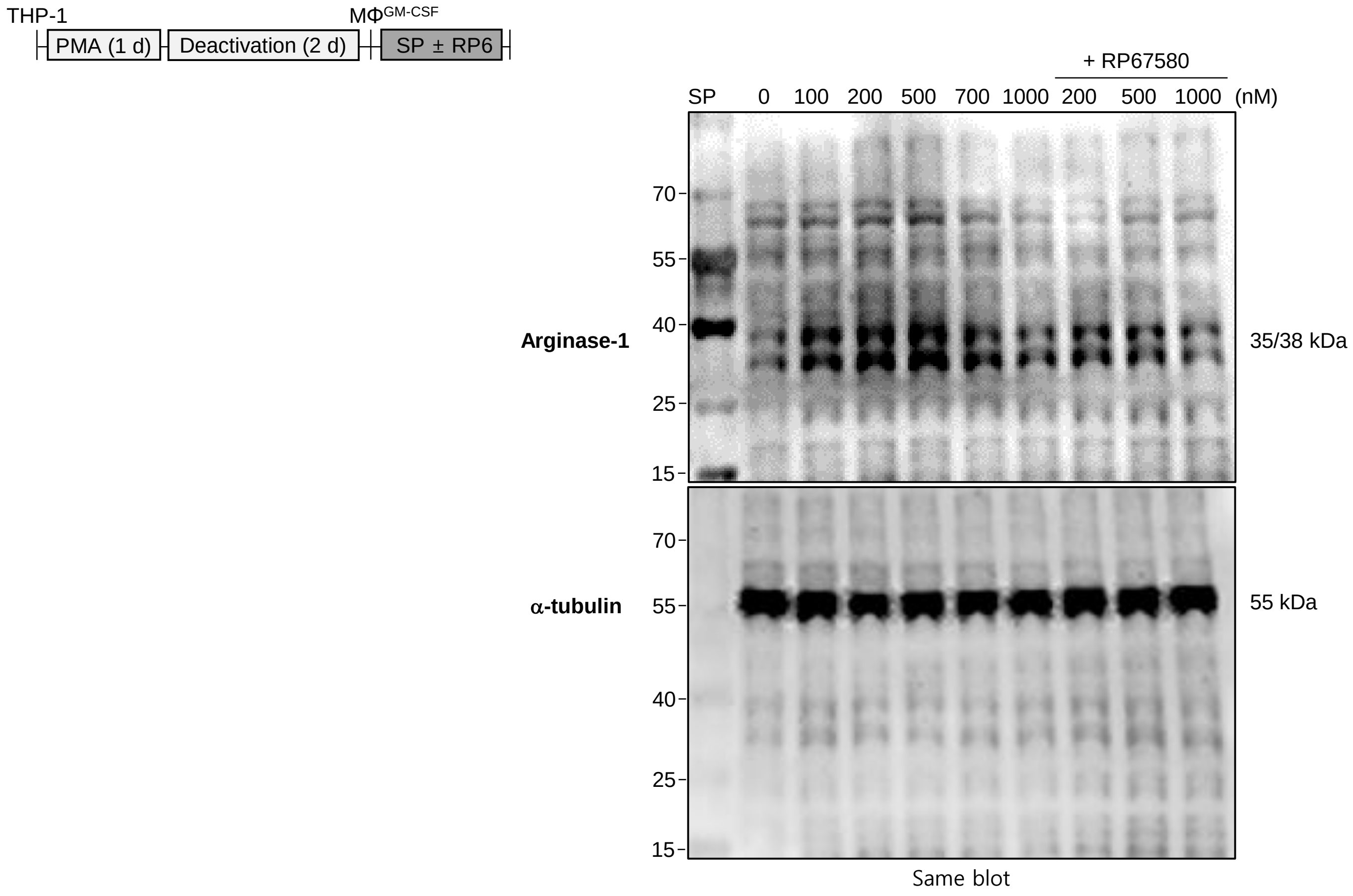

Supplementary Figure 10

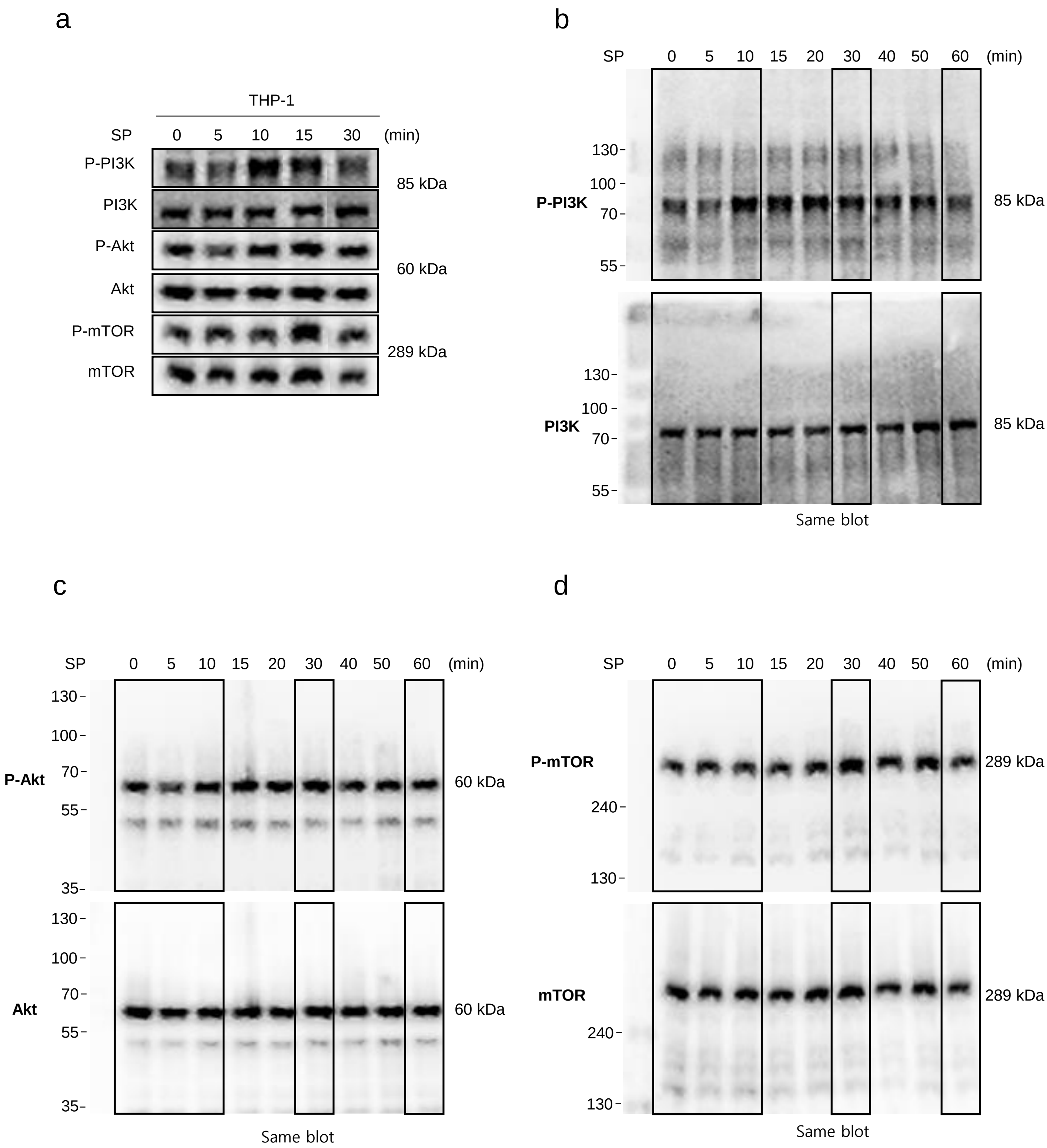

Supplementary Figure 11

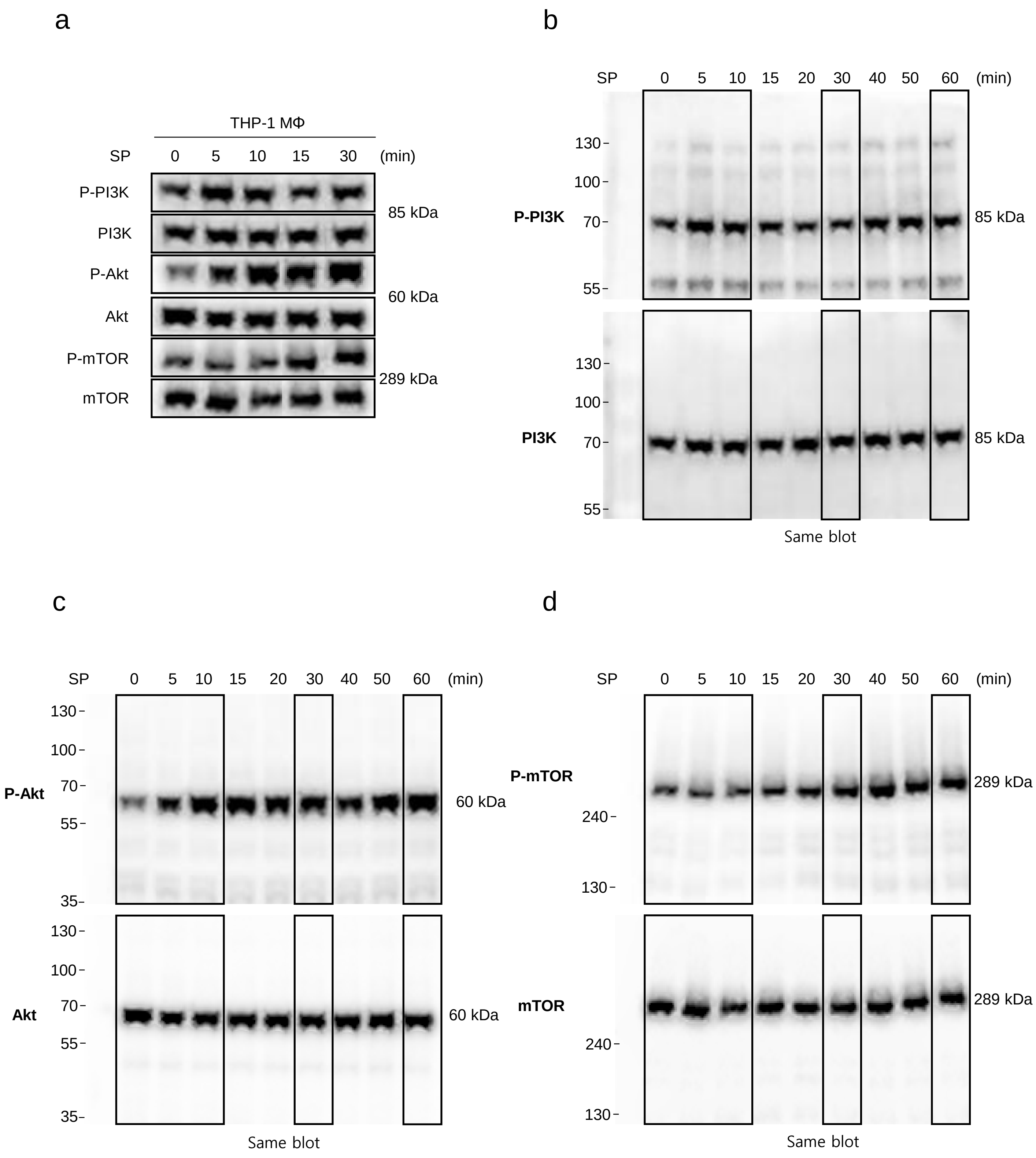

Supplementary Figure 12

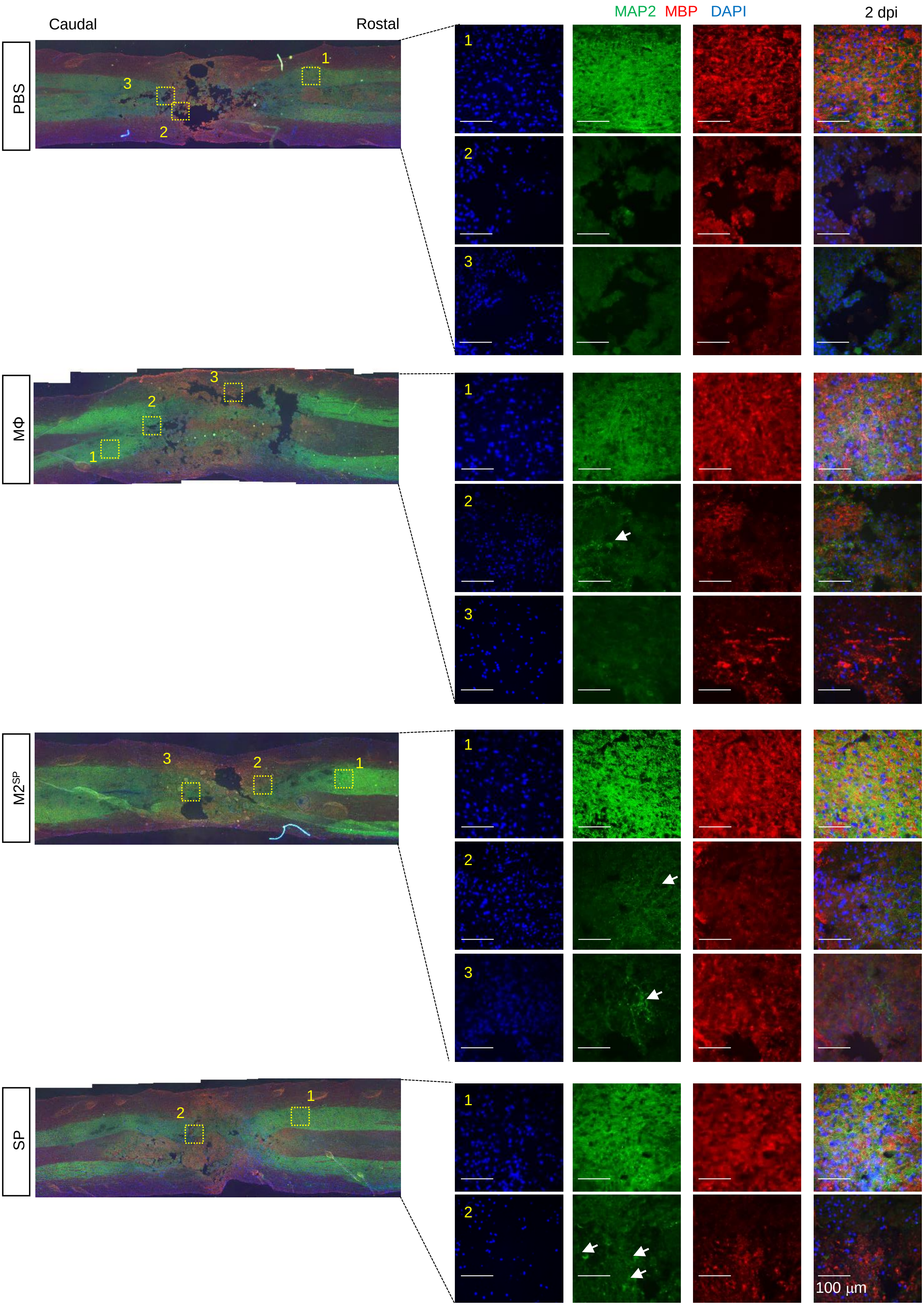

Supplementary Figure 13

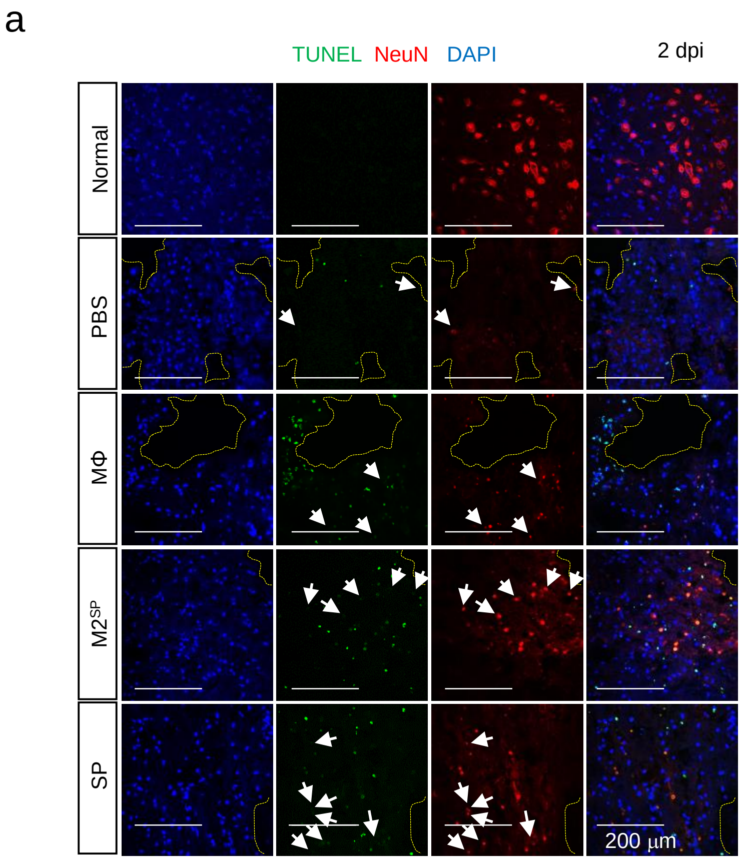

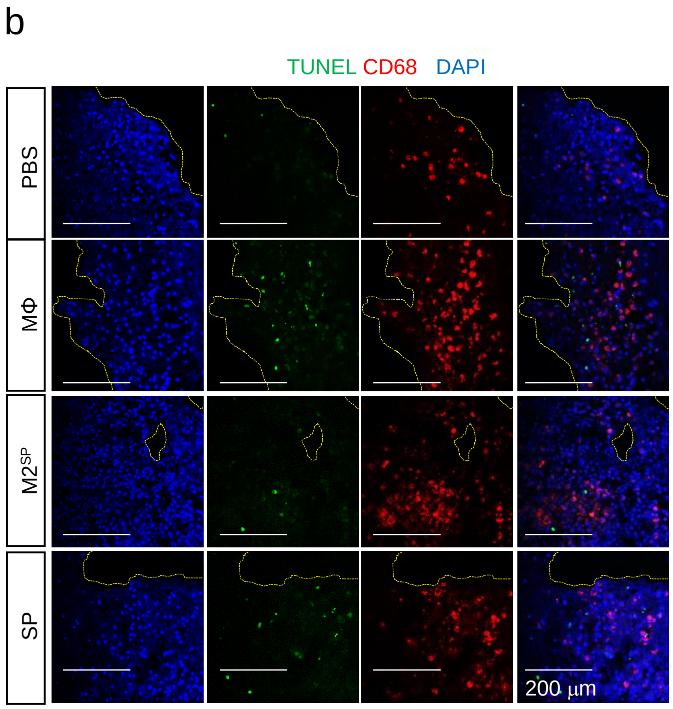

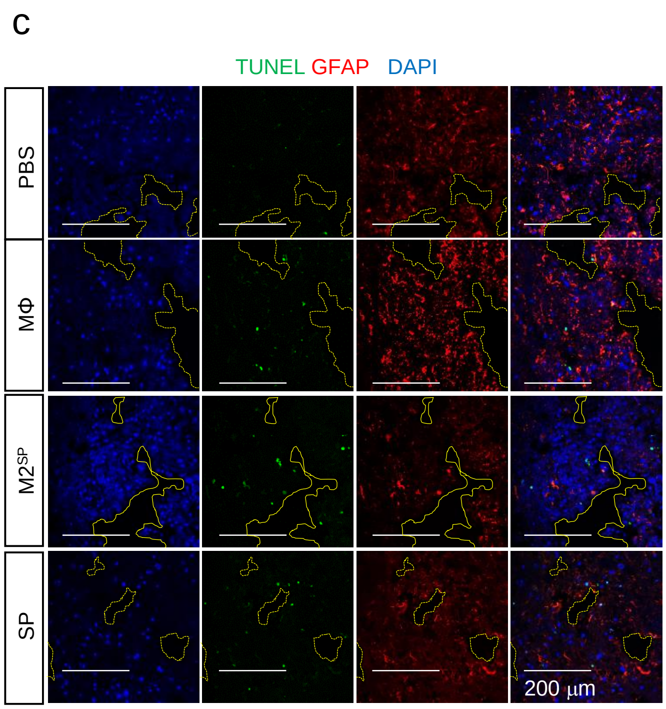

Supplementary Figure 14

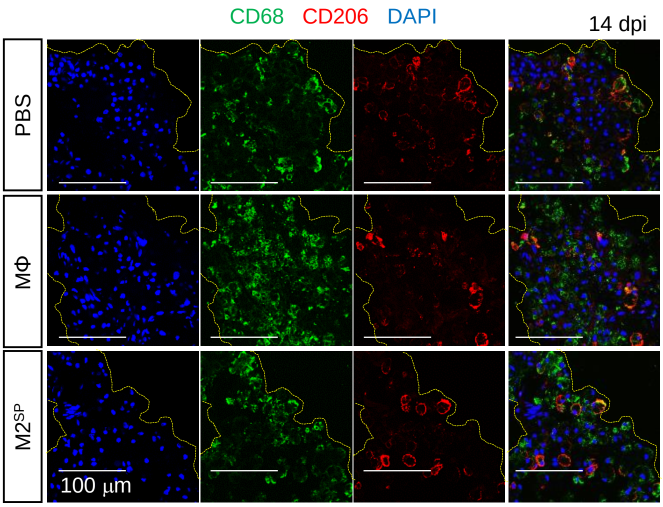

Supplementary Figure 15

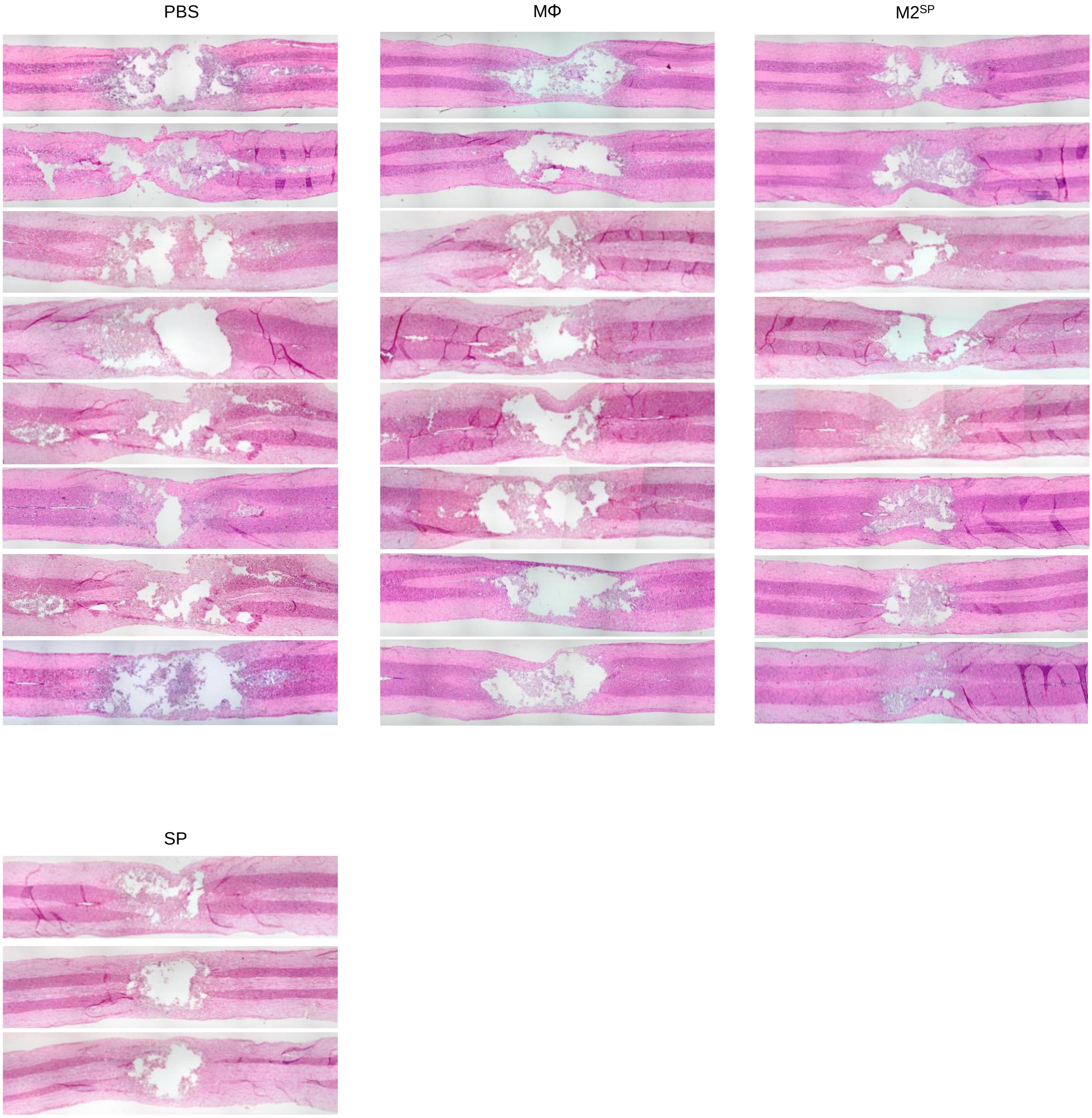

Supplementary Figure 16

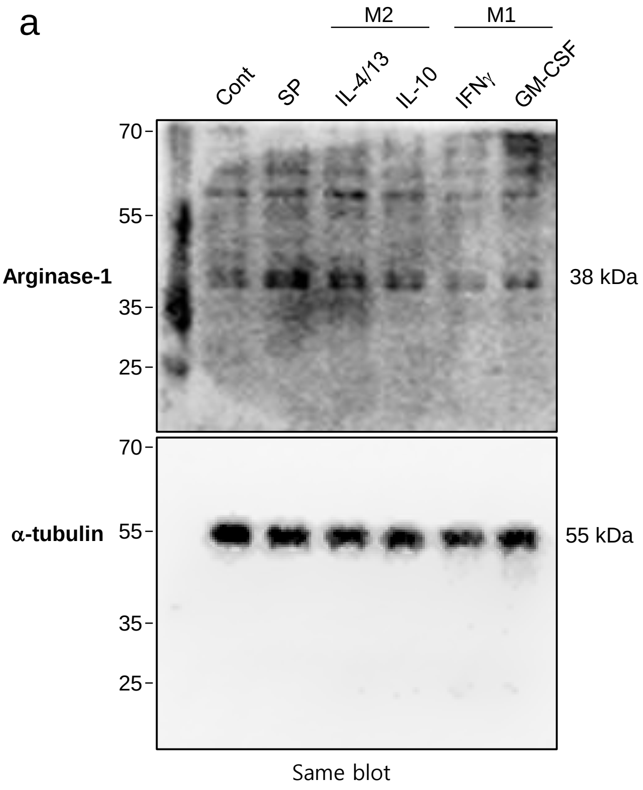

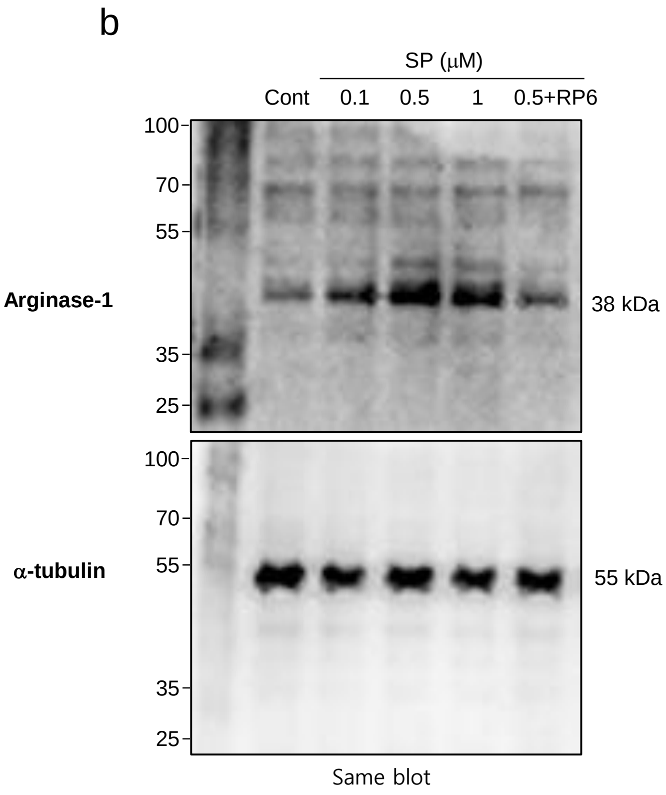

Supplementary Figure 17

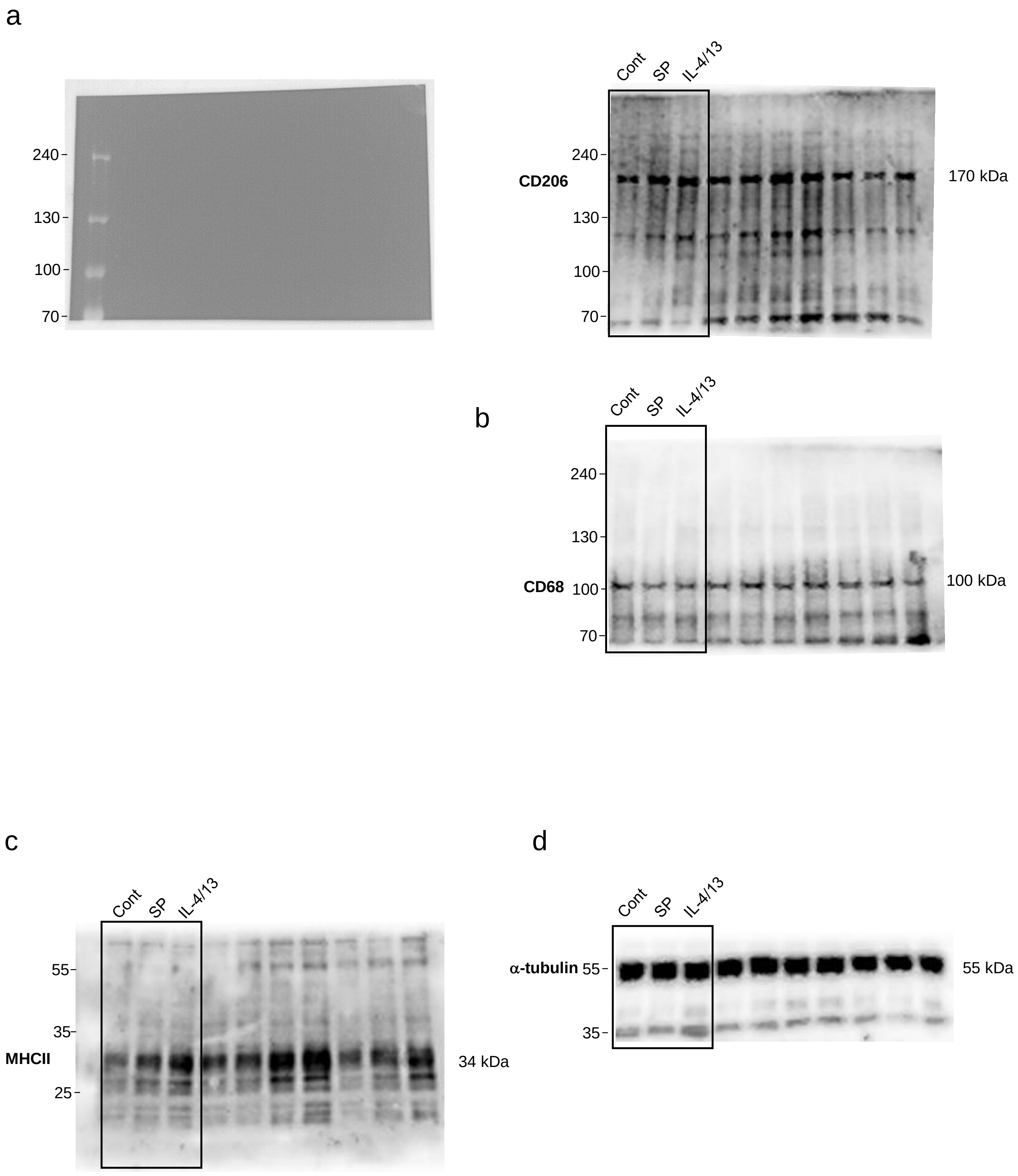

Supplementary Figure 18

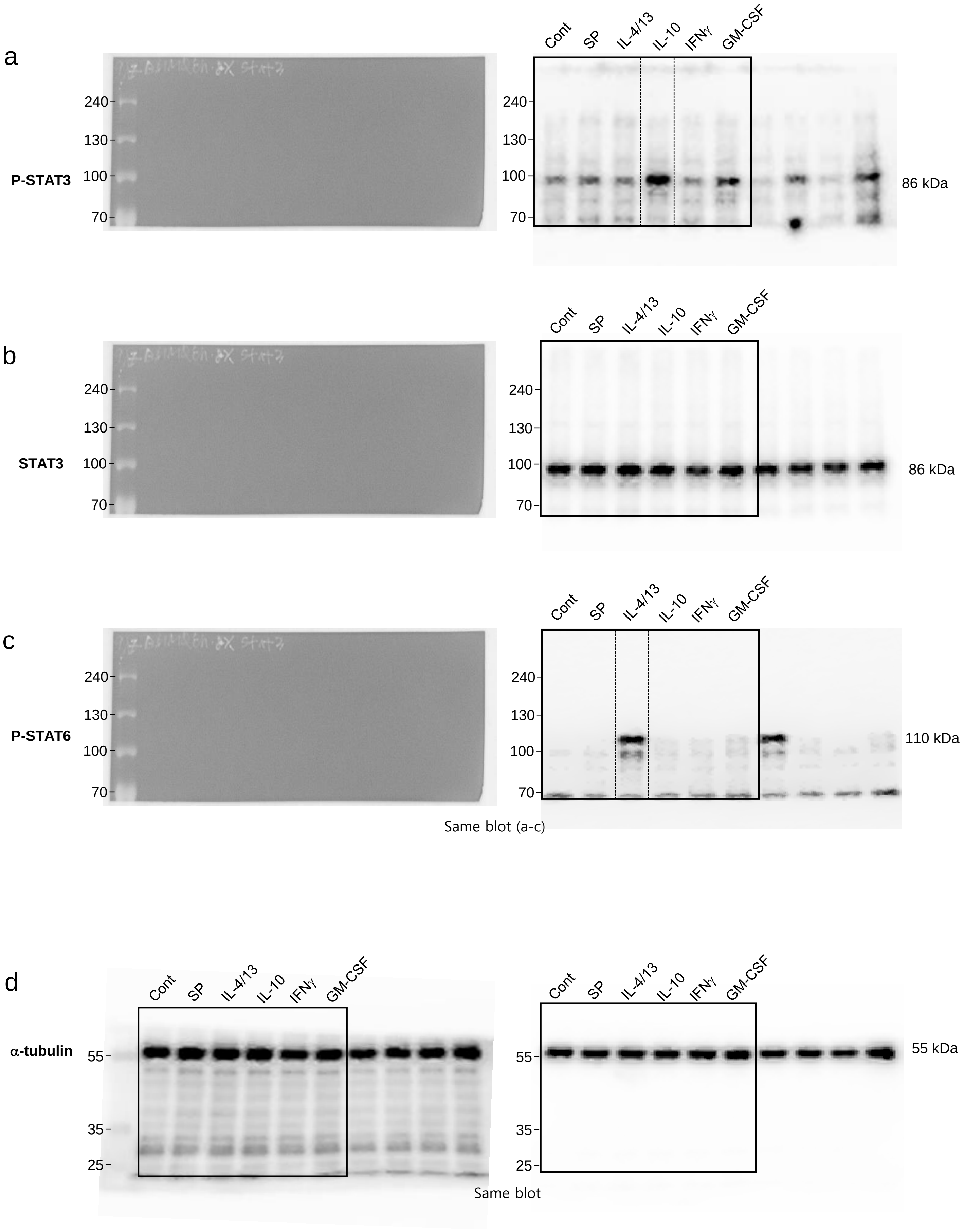

Supplementary Figure 19

a

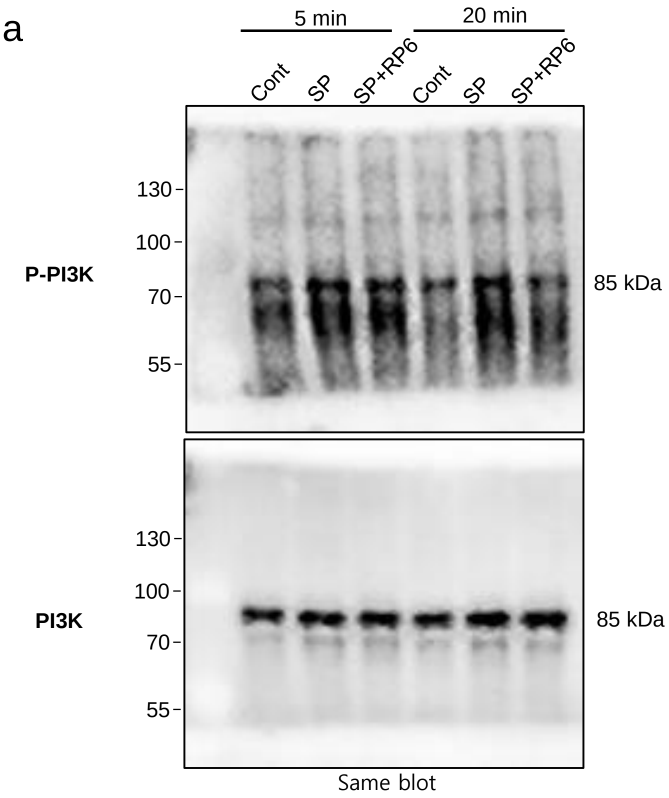

b

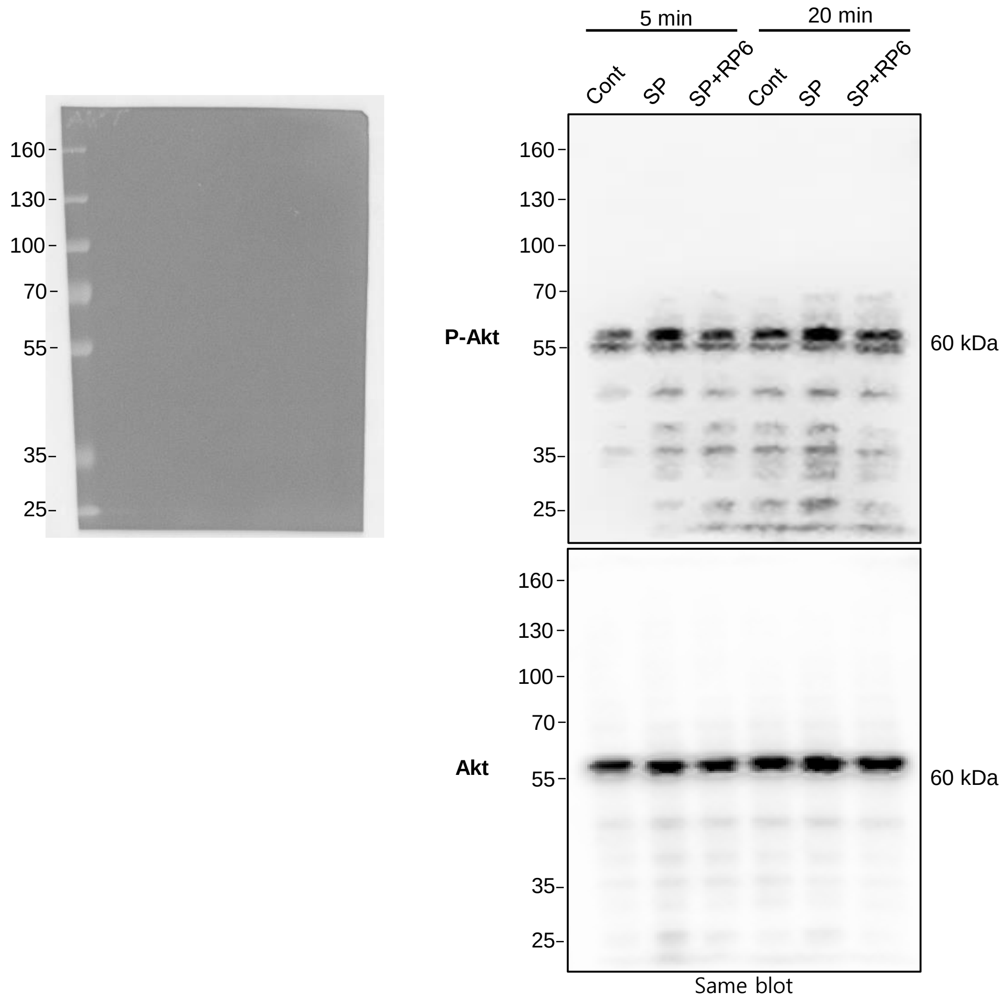

Supplementary Figure 20

a

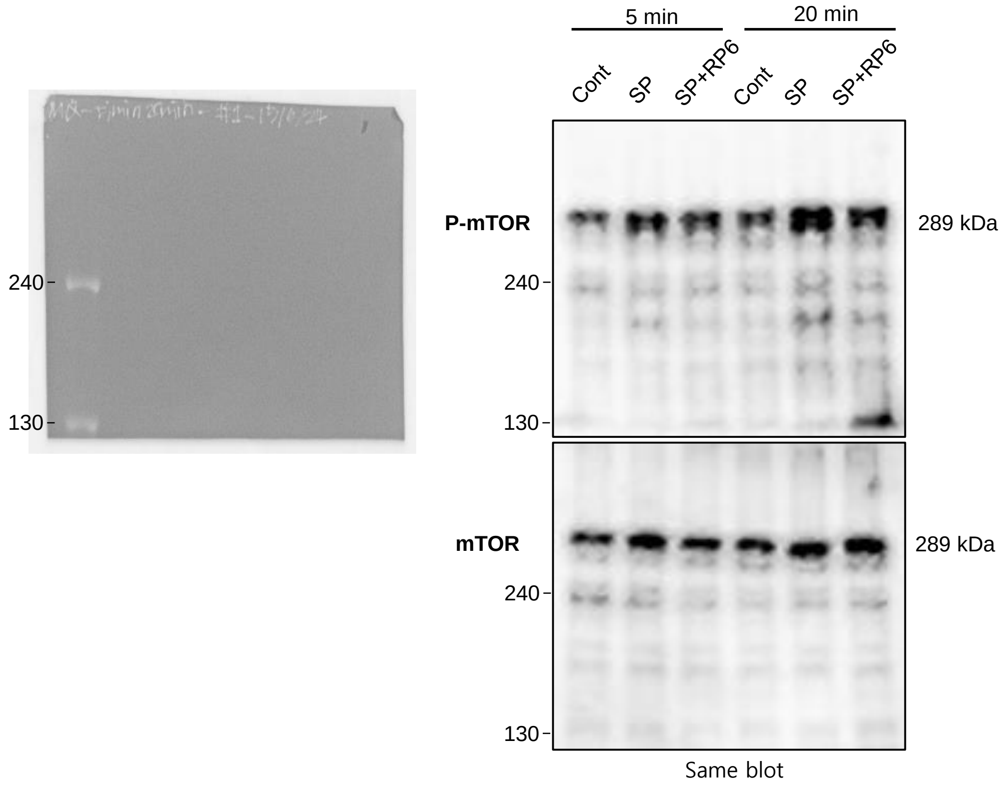

b

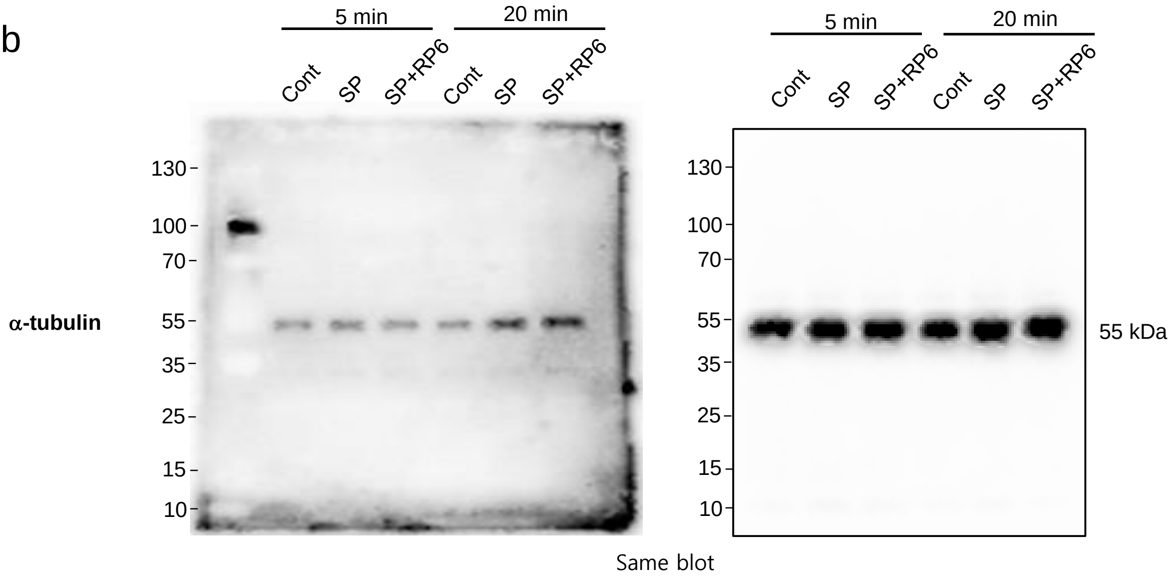

Supplementary Figure 21

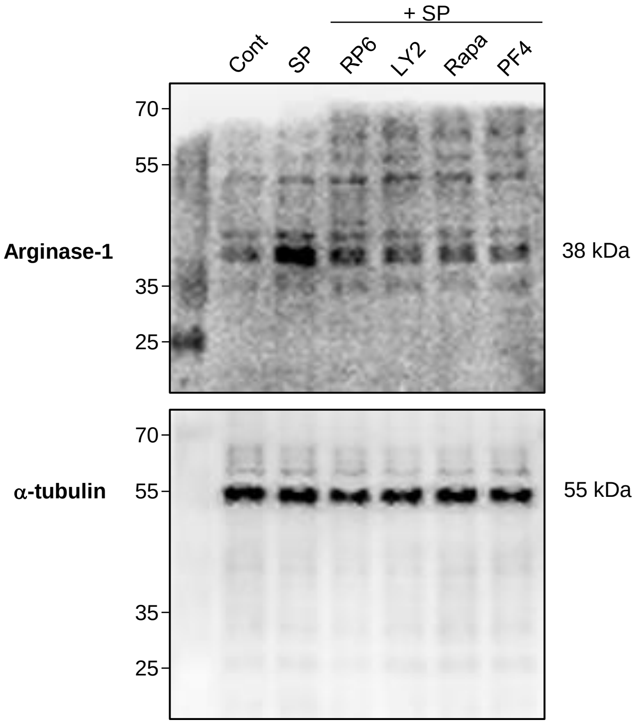

Same blot

Supplementary Figure 22

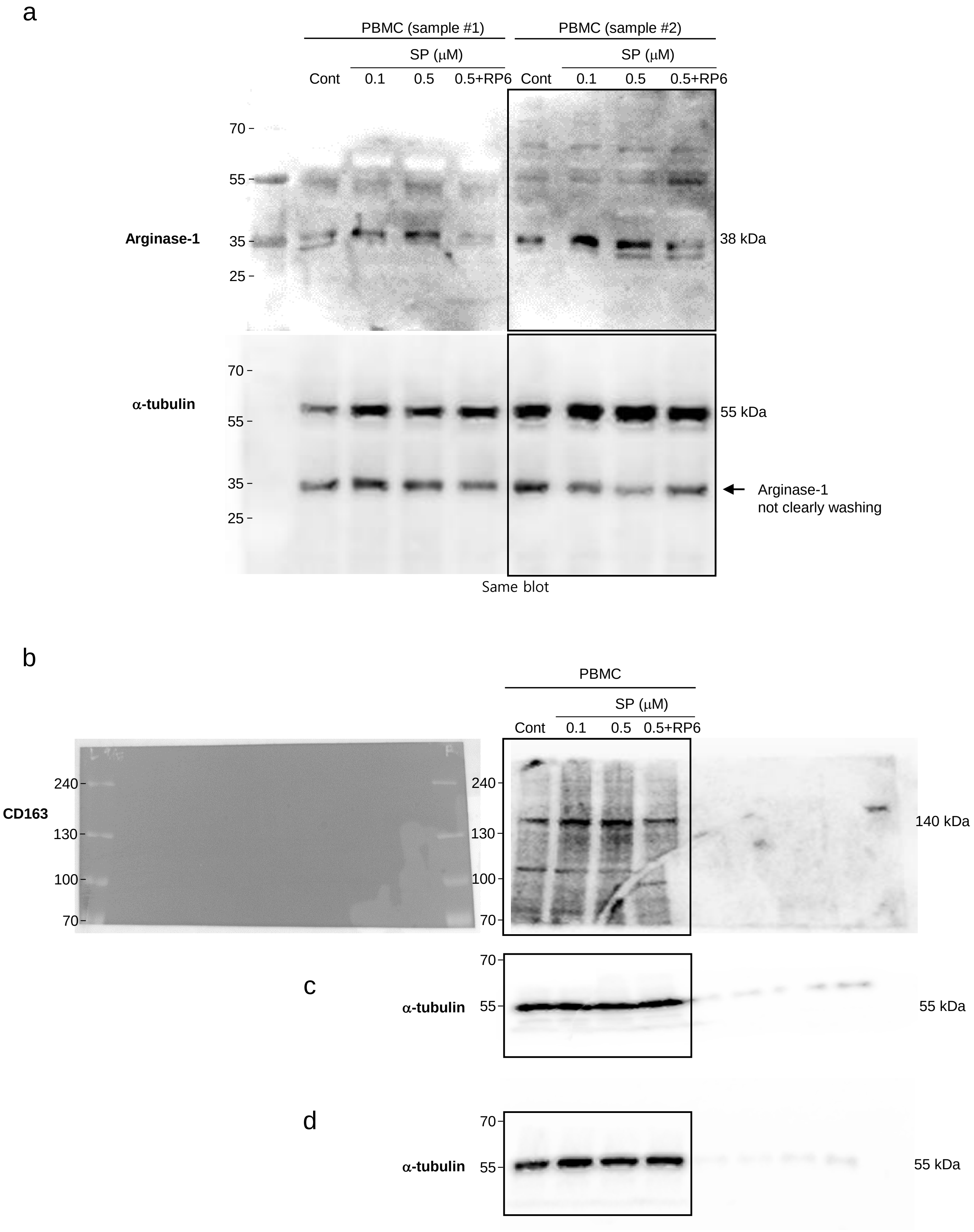

Supplementary Figure 23

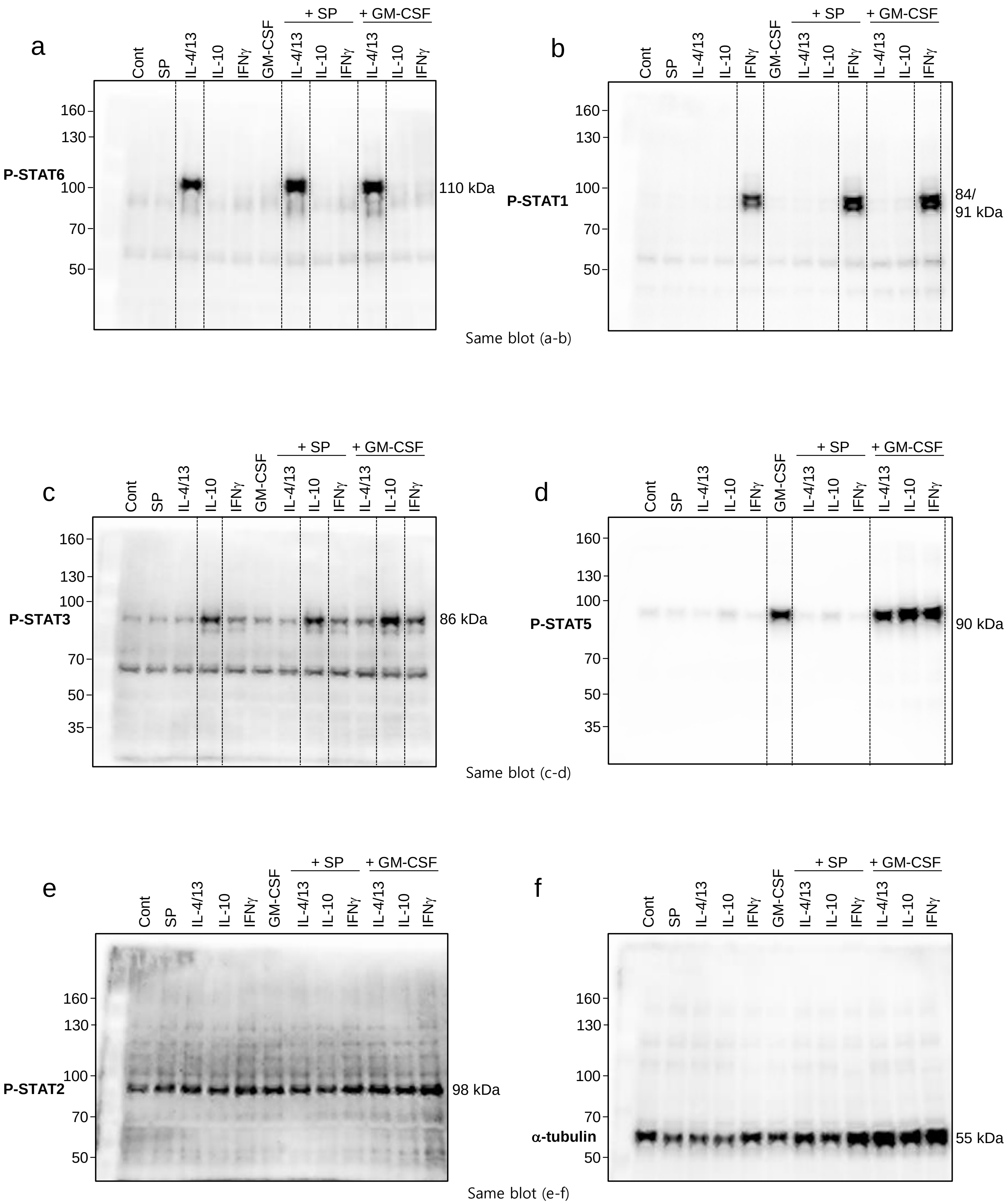

Supplementary Figure 24

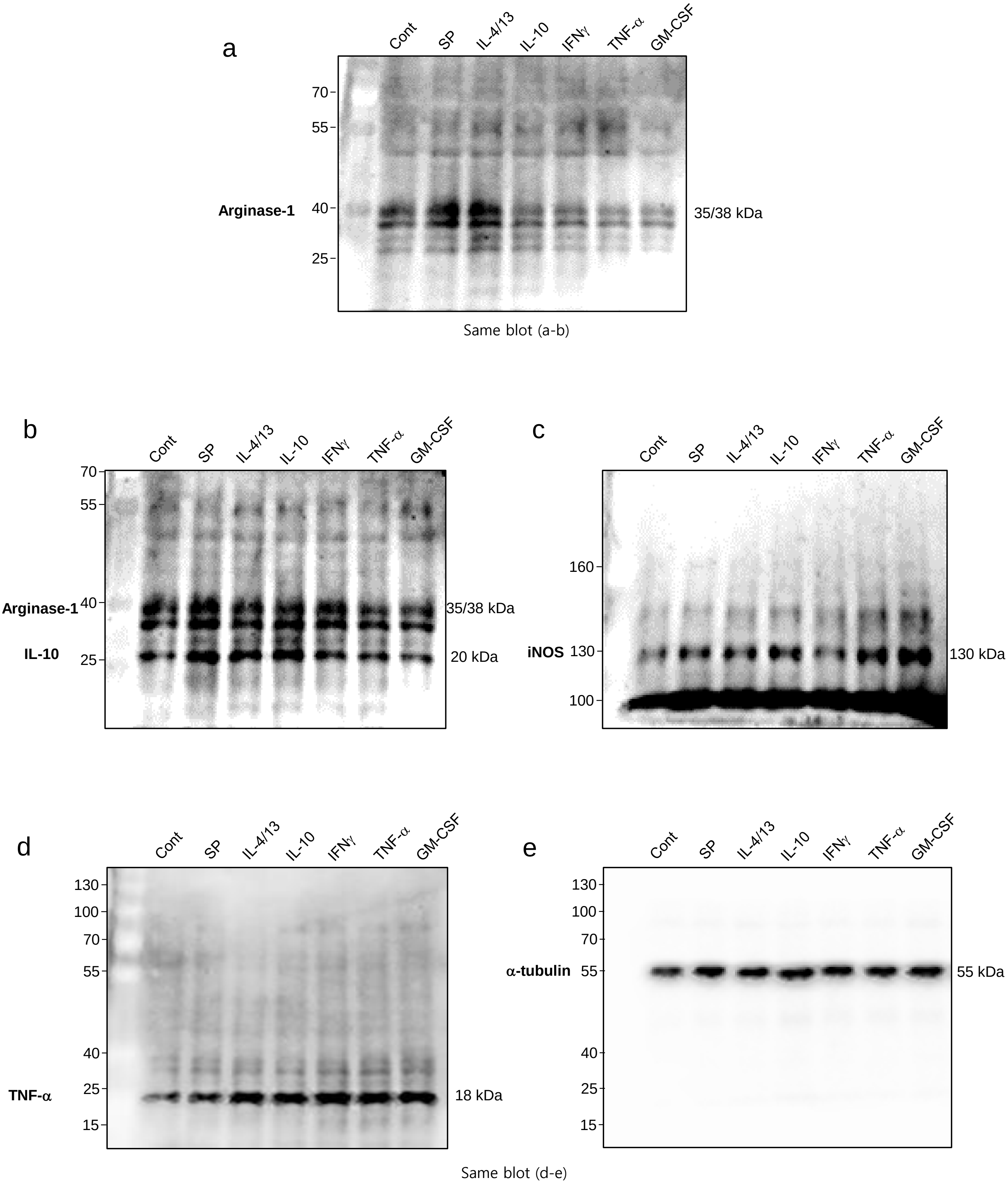

Supplementary Figure 25

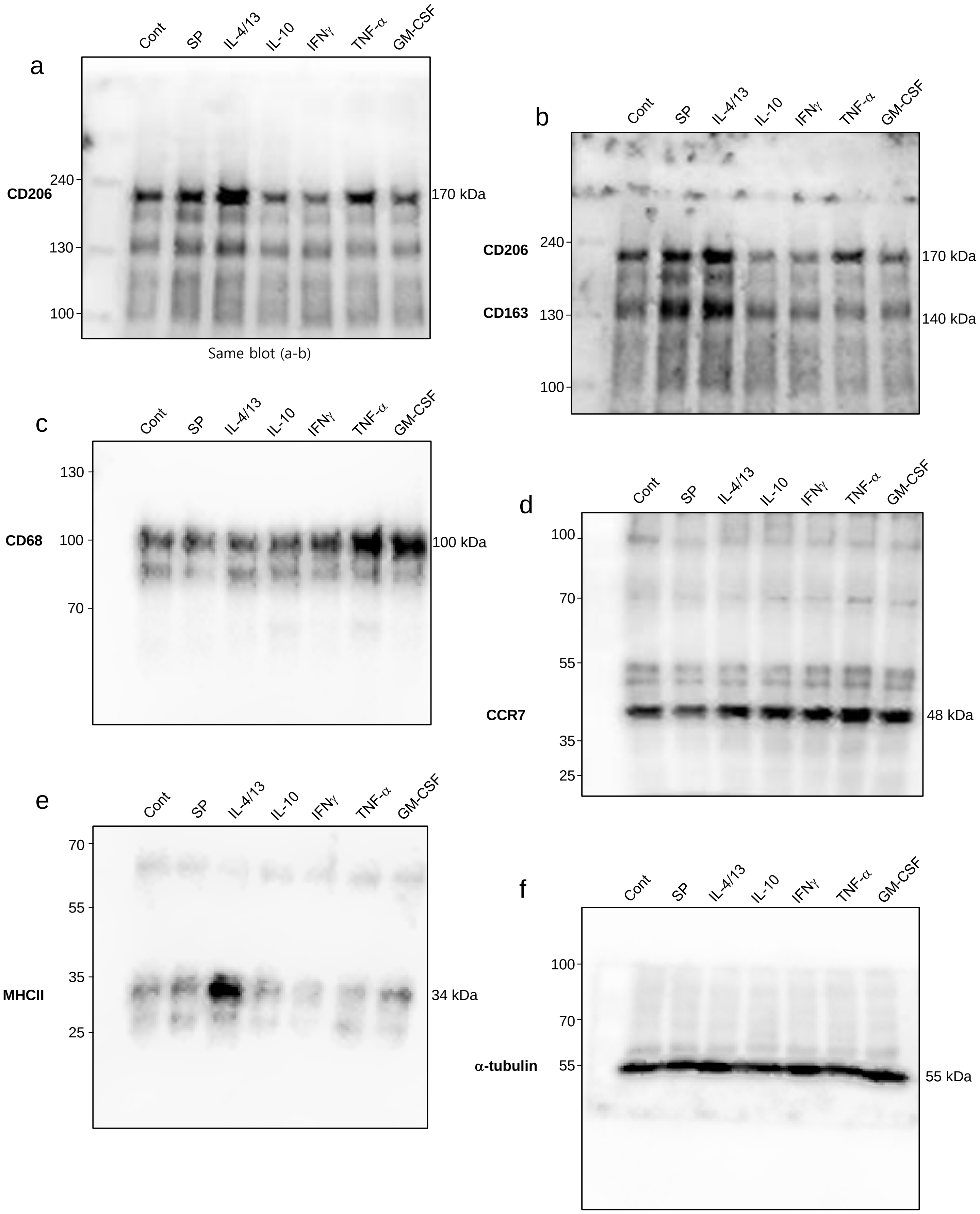

Supplement: Supplementary file 1 — supplementary information [file 41598_2017_9639_MOESM1_ESM.pdf]
